# Supplementary material for: Bacterial protoplast-derived nanovesicles carrying CRISPR-Cas9 tools re-educate tumor-associated macrophages for enhanced cancer immunotherapy
Source: Nat Commun. 2024 Jan 31;15:950. doi: 10.1038/s41467-024-44941-9 (PMC10830495; doi:10.1038/s41467-024-44941-9)
Supplement: Supplementary file 1 — Supplementary Information [file 41467_2024_44941_MOESM1_ESM.pdf]

Bacterial Protoplast-Derived Nanovesicles Carrying CRISPR Cas9 Tools Re-educate Tumor-Associated Macrophages for Enhanced Cancer Immunotherapy

Mingming Zhao[1#], Xiaohui Cheng[1#], Pingwen Shao[1], Yao Dong[1], Yongjie Wu[1], Lin Xiao[1], Zhiying Cui[1], Xuedi Sun[1], Chuancheng Gao[1], Jiangning Chen[1,2]*, Zhen Huang[1]*, Junfeng Zhang[1]*

[1]State Key Laboratory of Pharmaceutical Biotechnology, School of Life Sciences, Nanjing University, Nanjing, Jiangsu 210023, China

[2]State Key Laboratory of Analytical Chemistry for Life Sciences, Nanjing University, Nanjing, Jiangsu 210023, China

***Correspondence:** Jiangning Chen (jnchen@nju.edu.cn), Zhen Huang (zhenhuang@nju.edu.cn) or Junfeng Zhang (jfzhang@nju.edu.cn)

#The authors contributed equally: Mingming Zhao and Xiaohui Cheng

Supplementary methods

Synthesis of DSPE-hydrazone-mPEG$_{2000}$ (DHP)

For the synthesis of DHP, mPEG$_{2000}$-Ph-CHO was synthesized according to a previous report (yield ratio: 90%)[1]. The reaction intermediate (oxalic hydrazide) required for DHP synthesis was obtained as previously described[2]. Briefly, 445 mg oxamic acid which was dissolved in 30 mL ddH_2O, and 2.5 mL 10% hydrazine hydrate was stirred overnight at room temperature, and then the solvent was evaporated under reduced pressure to obtain oxalic hydrazide (yield ratio: 91%).

1 g mPEG$_{2000}$-Ph-CHO and oxalic hydrazide (48.4 mg) were dissolved in 10 mL ethanol and stirred for 6 h and then the solvent was removed by rotary evaporation under reduced pressure. The sediment was dissolved in 10 mL distilled water and then filtrated. The filtrate was extracted with dichloromethane (DCM) (10 mL×3), concentrated under reduced pressure, and poured into the ice-cold ether for precipitation. mPEG$_{2000}$-hydrazone-COOH was obtained by filtration and further dried under vacuum for 24 h (yield ratio: 90%). Then, 200 mg mPEG$_{2000}$-hydrazone-COOH was dissolved in 5 mL dimethylformamide (DMF). 2 mL chloroform containing 1,2-distearoyl-sn-glycero-3-phosphorylethanolamine (DSPE) (66.28 mg), 1-ethyl-3-(3-dimethylaminopropyl) carbodiimide (EDC) (27.57 mg), and 4-(dimethylamino) pyridine (DMAP) (1.0 mg) was added and reacted at 40°C for 12 h and then concentrated under reduced pressure and poured into ice ether for precipitation. The precipitate was first vacuum-dried and then redissolved in ethanol. It was subsequently dialyzed in double-distilled water using a membrane with a 2kDa molecular weight cutoff for 48 h. The sample underwent lyophilization afterwards, resulting in a 92% yield ratio of DHP. To determine its structure, DHP was analyzed using a ^{1}HNMR spectrometer (Bruker AVANCE III HD, Billerica, MA, USA). The ^{1}H NMR spectrum of DHP (400 MHz, DMSO) revealed the following chemical shifts: δ 11.37 (br, 1H), 8.44 (br, 1H), 7.98 (m, 3H), 7.76 (m, 2H), 5.60 (m, 1H), 4.74 (m, 2H), 4.55 (m, 2H), 4.30 (m, 2H), 4.00 (m, 2H), 3.68 (m, 2H), 3.51 (m, –CH_2 of PEG), 3.42 (m, 2H), 3.36 (m, 2H), 3.24 (s, 3H), 1.40 (m, 4H), 1.23 (m, –CH_2 of DSPE), 0.85 (t, 6H). ^{13}C NMR ($CDCl_3$, 101 MHz) δ 173.51, 173.13, 173.05, 171.54, 144.67, 135.92, 128.71, 127.94, 127.12, 72.72, 71.96, 70.59, 70.27, 64.16, 62.53, 61.72, 59.10, 45.78, 34.32, 34.14, 31.98, 29.77, 29.72, 29.58, 29.42, 29.20, 24.92, 22.75, 14.19. In the FT-IR spectrum, the bound assignments of DHP were described below: 1103 cm^{-1}, C-O of PEG$_{2000}$, stretching vibration; 1464 cm^{-1}, CH_2 bending vibration; 1647 cm^{-1}, C=O, stretching

vibration (amide); 1736 cm^{-1}, C=O, stretching vibration (ester); 2916 cm^{-1},2882 cm^{-1}, C-H, stretching vibration; 3433 cm^{-1}, N-H stretching vibration.

Rhodamine B labeled DHP (RhB-DHP) was synthesized as following described. 3 g Boc-NH-PEG_{2000}-NH_2 was dissolved in 10 mL DMF, and then Rhodamine B (0.68 g), EDC (0.44 g) and DMAP (0.017 g) were added. The mixture was stirred overnight at room temperature, concentrated by rotary evaporation and precipitated with ice-cold ether. RhB-PEG_{2000}-NH-Boc was collected via filtration and further dried under vacuum for 24 h (yield ratio: 90%). 6 mL DCM containing 2 g RhB-PEG_{2000}-NH-Boc and 2 mL trifluoroacetic acid was mixed. The reaction was kept in an ice bath with magnetic stirring for 1 h. The reactant was washed with distilled water for 3 times and dried with anhydrous sodium sulfate. The separation steps of RhB-PEG_{2000}-NH_2 from reactant were the same as the previous steps of RhB-PEG2000-NH-Boc. The product yield of the reaction is 92 %. 2 g RhB-PEG_{2000}-NH_2 dissolved in 10 mL DCM, and 2 mL DMF solution containing 1,4-carboxybenzaldehyde (0.14 g), N, N'-dicyclohexylcarbodiimide (DCC) (0.33 g), and DMAP (0.098 g) was added and reacted at room temperature for 24 h. The solvent was removed by rotary evaporation under reduced pressure. RhB-PEG_{2000}-Ph-CHO was obtained from the sediment according to the separation protocols of mPEG_{2000}-hydrazone-COOH. The product yield of the reaction is 90%. The following procedure to obtain the final product RhB-PEG_{2000}-hydrazone-DSPE referred to the synthesis protocols of mPEG_{2000}-hyd-DSPE. The fluorescence spectra of RhB-labeled DHP were scanned by a micro plate reader (Thermo Fisher, Waltham, MA, USA) to ensure the successful conjugation of the fluorescent probe to DHP.

Synthesis of DSPE-galactose (DGA)

DGA was synthesized via one-step reaction[3]. Typically, 200 mg of 1,2-distearoyl-sn-glycero-3-phosphoethanolamine-N-Succinimidyl Ester was dissolved in 10 mL chloroform. Subsequently, a solution of 5 mL DMSO, containing 35.82 mg of galactosamine and 20.25 mg of triethylamine, was added. The mixture was stirred at room temperature for 6 h, after which it was poured into acetone to induce precipitation. The resulting precipitate was collected, vacuum-dried, and then redissolved in DMSO. It was then dialyzed against double distilled water for 48 h using a membrane with a 500 Da molecular weight cutoff, followed by lyophilization to obtain DGA with a 90% yield ratio. The ^{1}H NMR spectrum of DGA (400 MHz, $CDCl_3$) showed chemical shifts at δ 7.58 (br, 2H), 5.26 (br, 4H), 4.70 (br, 1H), 4.41 (m, 2H), 4.18 (m, 2H), 4.02 (br,

3H), 3.76 (m, 4H), 3.50 (m, 4H), 2.30 (m, 8H), 1.63 (m, 8H), 1.29 (m, -CH_2 of N-succinimidyl DSPE), 0.92 (t, 6H). ^{13}C NMR (DMSO, 101 MHz) δ 173.26, 172.75, 91.64, 70.86, 70.13, 68.95, 67.41, 63.78, 63.50, 63.26, 62.84, 61.31, 50.96, 35.81, 34.11, 33.95, 31.84, 29.60, 29.27, 29.02, 28.69, 25.60, 24.96, 22.64, 14.44. In the FT-IR spectrum, the bound assignments of DGA were described below: 723 cm^{-1}, -$(CH_2)_n$- of DSPE; 1051 cm^{-1}, C-O stretching vibration; 1466 cm^{-1}, CH_2 bending vibration; 1645 cm^{-1}, C=O, stretching vibration (amide); 1742 cm^{-1}, C=O, stretching vibration (ester); 2920 cm^{-1}, 2851 cm^{-1}, C-H, stretching vibration; 3314 cm^{-1}, O-H, stretching vibration.

The Cy5.5-labeled DGA was synthesized according to following described. Briefly, 200 mg galactosamine was dissolved in 3 mL DMSO. Then, Cy5.5-NHS (1.0 g), and triethylamine (0.28 g) were added and reacted at room temperature for 5 h. The reaction solution was poured into ice ether for precipitation. The precipitate was collected by centrifugation, then acetone was used to wash it for 3 times, and Cy5.5-galactose was obtained by vacuum drying. The product yield was 85%. Then, 2 mL DMSO containing 100 mg Cy5.5-galactose, 12.0 mg succinic anhydride and 20.2 mg triethylamine was stirred at room temperature for 8 h. Then, Cy5.5-galactose-COOH was obtained from the reactant according to the separation protocols of Cy5.5-galactose (yield ratio: 89%). Finally, 50 mg DSPE was dissolved in 3 mL chloroform, and DMSO solution containing COOH-galactose-Cy5.5 (80.31 mg), EDC (15.63 mg), and DMAP (0.8 mg) was added and reacted at 40 ºC for 8 h. The chloroform was removed by rotary evaporation under reduced pressure, then the sediment was dialyzed (molecular weight cut-off: 500) for 24 h in distilled water and lyophilized to obtain DSPE-galactose-Cy5.5 (yield ratio: 90%).

The FITC-labeled DGA was synthesized according to following described. Initially, 100 mg of lactose was dissolved in 5 mL of dimethylformamide (DMF). Then, Fmoc-L-Lys(Boc)-OH (0.2601 g), PyBOP (0.3466 g), and triethylamine (0.1123 g) were added and reacted at room temperature for 0.5 h. The reaction mixture was concentrated under reduced pressure. The reaction solution was poured into ice ether for precipitation. The precipitate was collected by centrifugation, then acetone was used to wash it for 3 times and Fmoc-L-Lys (Boc)-lactose was obtained via vacuum drying. Subsequently, 100 mg of Fmoc-L-Lys (Boc)-lactose was weighed and dissolved in a methanol-hydrochloric acid mixture (volume ratio 1:5). After a 0.5-hour reaction at room temperature, the solvent was removed under reduced pressure to yield Fmoc-L-Lys (NH2)-lactose. This product was then dissolved in 5 mL of DMF, and FITC (0.0705 g)

and triethylamine (0.0367 g) were added. The reaction was allowed to proceed for 0.5 h at room temperature. Following this, 2 mL of pyridine were introduced, and the mixture was stirred at room temperature for 1 hour. Subsequently, the reaction mixture was concentrated under reduced pressure. Ice-cold ether was used for precipitation, and the product was collected through filtration with a sand core funnel. The final step involved vacuum drying to obtain NH2-L-Lys (FITC)-lactose. 100 mg of NH2-L-Lys (FITC)-lactose was weighed and dissolved in 5 mL of DMF. DSPE-COOH (0.1189 g), PyBOP (0.0876 g), and triethylamine (0.0284 g) were then added until complete dissolution was achieved. After a 0.5-hour reaction period at room temperature, the reaction mixture was concentrated under reduced pressure. Ice-cold ether was used for precipitation, and the product was collected through filtration with a sand core funnel. The final step involved vacuum drying to obtain DSPE-L-Lys (FITC)-lactose. The fluorescence spectra of Cy5.5 or FITC-labeled DGA were scanned by a micro plate reader (Thermo Fisher) to ensure the successful conjugation of the fluorescent probe to DGA.

Differential Scanning Calorimetry (DSC) analysis

7×10^{10} sg*Pik3cg*-NV and 7×10^{10} sg*Pik3cg*-DHP/DGA-NV were resuspended in 50 μL PBS. DHP or DGA was suspended in PBS to achieve a final concentration of 35 mg/mL and 20 mg/mL, respectively. 30 μL of above-mentioned sample was transferred into a 40 μL DSC aluminum pan and subjected to DSC analysis using a DSC1 (Mettler Toledo, Zurich, Switzerland). The scan rate employed was 5℃/min and the reference pan was filled with 30 μL PBS.

FRET

Fluorescence resonance energy transfer (FRET) study was used to conduct the DHP and DGA decoration and pH-responsive bone cleavage of DHP. FITC-DGA and RhB-DHP were applied to prepare RhB-DHP/FITC-DGA-NV, RhB-DHP/DGA-NV and DHP/FITC-DGA-NV. The fluorescence spectra of different types of NV were screened by a microplate reader (Thermo Fisher). (Em:488 nm; Ex:500-700nm) for DHP/FITC-DGA-NV and RhB-DHP/FITC-DGA-NV. (Em:520 nm; Ex:550-700nm) for RhB-DHP/DGA-NV.

Primary cell isolation and cell culture

Bone marrow-derived macrophages (BMDM) were induced as previously reported[4]. Briefly, bone marrow cells were rinsed from the tibia and femora of C57BL/6J mice

and cultured in Dulbecco's modified Eagle medium (DMEM) (Life Technology, Grand Island, NY, USA) medium with 10% fetal bovine serum (FBS) (Life Technology) and 20 ng/mL macrophage colony-stimulating factor (M-CSF, PeproTech, Cranbury, NJ, USA) for 7 days. Then, BMDMs were treated with 40 ng/mL IL-4 (PeproTech) for 2 days for M2 polarization.

Tumor leukocytes were isolated as following described. Tumor tissue was first minced in cold PBS and resuspended in DMEM containing 0.1% collagenase IV (weight per volume) and 0.1% DNase I (weight per volume) for 15 min at 37 °C, smashed with gentle MACS Dissociator (Miltenyi, Bergisch-Gladbach, Germany), lysed with red blood cell lysis buffer and filtered through 70 μm Nylon cell strainers (Falcon, New York, NJ, USA) to obtain single-cell suspensions. Leukocytes were further isolated via gradient centrifugation using Mouse Tumor infiltrating Leukocyte Isolation Kit (Solarbio, Beijing, China). Splenic macrophages, tumor-associated macrophages (TAMs) and neutrophils were isolated from the tumor leukocytes using F4/80 micro beads and neutrophil isolation kit (Miltenyi). The remaining immune cell populations after F4/80 micro beads were also collected for T7E1 analysis. Tumor fibroblast, endothelial cells and tumor cells were isolated according to a previous report[5]. Hepatocytes, endothelial cells, macrophages and other immune cells from mouse liver were isolated as previously described[6]. Peripheral blood neutrophils were also sorted using magnetic beads (Miltenyi) and the remaining immune cells populations were also collected. 293T cells and RAW 264.7 cells were cultured in DMEM containing 10% FBS. 4T1 and MC38 cells were cultured in Roswell Park Memorial Institute medium (RPMI)-1640 containing 10% FBS. All cells were cultured in an incubator with a humidified atmosphere of 5% CO_2 at 37°C.

Plasmid Construction

sgRNAs against *Pik3cg* gene were designed according to the online tools https://zlab.bio/guide-design-resources. Synthesized sgRNAs (Sangon Biotech, Shanghai, China) were cloned into the plasmid vector (pSpCas9(BB)-2A-GFP, PX458, #48138, Addgene, Watertown, MA, USA). To screen the gene editing efficiency, plasmid vectors carrying different sgRNA sequences were transfected into RAW 264.7 cells using Lipofectamine 2000 (Life Technology) and genomic DNA was extracted for T7 Endonuclease I (T7E1) assay. Then, the selected sgRNA sequence-1# with the highest editing efficiency against *Pik3cg* gene or corresponding control sgRNA sequence was inserted into bacterial expression vector BPK764 (#65767, Addgene).

Component analyze of *E. coli* protoplast-derived nanovesicles (NVs)

To analyze the nucleotide content in NVs, DNA and RNA were isolated from *E. coli* and NVs using a Genomic DNA Extraction kit (TIANGEN, Beijing, China) and TRIzol reagent (Thermo Fisher) according to the manufacturer's instructions, respectively. Then, the DNA and RNA were examined by LabChip GX (PerkinElmer, Waltham, MA, USA) and Agilent 2100 Bioanalyzer (Santa Clara, CA, USA).

Total protein of *E. coli*, *E. coli* protoplast, outer membrane vesicles (OMVs) and *E. coli* protoplast-derived NVs was extracted using RIPA Lysis Buffer containing 1% protease inhibitor cocktail, followed by ultrasound sonication on ice. Each sample, containing 100 μg of protein, was then subjected to LC-MS analysis on a Shimadzu UFLC 20ADXR HPLC system, connected in line with an AB Sciex 5600 Triple TOF mass spectrometer (AB SCIEX, Waltham, MA, USA). The obtained data were processed with MaxQuant (v. 1.6.7.0) and searched using the integrated Andromeda search engine against the UniProt database for *E. coli* BL21 (DE3) (https://www.uniprot.org/uniprotkb?facets=reviewed%3Afalse&query=BL21%28DE3%29&view=cards, downloaded on 23 September 2022). The iBAQ protein intensity, as outlined in the literature[7, 8], was used to rank the abundance of distinct proteins within group. The top 200 proteins with the highest iBAQ values were classified by subcellular localization using Psortdb (https://db.psort.org/). COG analysis was conducted on the top 200 abundant proteins of sg*Pik3cg*-DHP/DGA-NV to determine their functional categories (http://eggnog-mapper.embl.de/). The comparison of outer membrane and periplasmic proteins between *E.coli*-OMV and sg*Pik3cg*-DHP/DGA-NV was performed by taking the logarithm of protein LFQ intensity values, represented by a normalized intensity profile generated using a specific algorithm[9]. Protein LFQ intensities were log2-transformed before heat mapping to reduce distributional skew and to give approximate normality, as previously reported[10].

The screen and identification of NVs encapsulated CpG-rich DNA sequences

Bioinformatics analysis was performed to screen the CpG-rich sequences in *E. coli* genome via the cpgplot program of EMBOSS based on the following criterion: the length was more than 800 base pairs and the ratio of C/G base was higher than 60%[11]. Genomic DNA was extracted from *E. coli* and nanovesicles by using a Genomic DNA Extraction Kit (TIANGEN) and used for PCR amplification of two CpG-rich DNA sequences which contain most type 1 motifs (CpG-rich sequence-9# and CpG-rich sequence-13#). The immunostimulatory effects of the PCR amplicons of CpG-rich

sequences localized in *E. coli* DNA, *E. coli* DNA, *E. coli* protoplast-derived NVs encapsulated DNA were evaluated in BMDMs by examining TNF-α levels.

ELISA assays.

Whole MC38 and 4T1 tumors were homogenized in cold PBS using a rotor stator (5 mm beads, 60 s × 2 replications at 60 Hz, Tissuelyser-24, Jingxin Industrial Development Co., Ltd., Shanghai, China) and then centrifuged at 12,000 rpm for 10 minutes at 4 °C to obtain the supernatants. TAMs isolated from MC38 and 4T1 tumors were lysed and the protein concentrations of lysate were determined using a BCA protein assay (Sangon Biotech). The supernatant of 4T1 cell and BMDMs with different treatments were collected. The levels of cytokines were determined using ELISA kits (Thermo Fisher) following the manufacturer's instructions and further normalized to total volume (supernatants) or mg total protein (lysates).

Tissue distribution assay of NVs in 4T1 tumor bearing mice

For a single administration of 1×10^{10} Cy5-labeled NVs, the plasma from mice after NVs injection were collected at indicated time points (1 min, 3 min, 30 min, 1 h, 3 h and 6 h). Tumor tissue and different organs were harvested. 100 mg of different tissue was mixed with 500 μL of PBS, used for the preparation of tissue homogenate, followed by centrifugation at 12,000 rpm for 10 minutes. The plasma and the supernatant were collected for the measurement of fluorescence intensity (Em: 644 nm; Ex: 665 nm). For repeated administration of NVs, mice were given with 1×10^{10} Cy5-labeled sg*Pik3cg*-DHP/DGA-NVs every two days via tail vein injection and were photographed by the IVIS spectrum system (PerkinElmer) and quantified. Blood samples were collected at indicated time points (1 h, 3 h, 6 h, 12h, 48 h) of each dosing cycle. The tumor tissues and plasma were used to determine fluorescence intensity. TAMs were also isolated for flow cytometry and gene-editing efficiency by T7E1 assay.

Luciferase reporter assay

Luciferase reporter assay was applied to check whether the immunostimulatory effect of NVs was dependent on the TLR9 signaling pathway. TLR9 overexpression plasmid (pECMV-Tlr9-m-FLAG), NF-κB reporter plasmid (pNFκB-TA-luc) and β-gal reference plasmid (pCMV-β-gal) were simultaneously transfected into 293T cells via Lipofectamine 2000 (Thermo Fisher). 24 h after transfection, 293T cells were treated with NVs for another 6 h. Under some circumstances, ODN2088 (TLR9 inhibitor, final concentration: 10 μM) and ODN1826 (TLR9 agonist, final concentration**:** 10 μM) were also added before NVs incubation or alone, respectively. Then, cells were lysed to

examine luciferase activity 24 h post to the addition of NVs. The β-gal assay was conducted for normalization.

PI3 kinase assay

sg*Pik3cg*-DHP/DGA-NV treated BMDMs or isolated TAMs were lysed in 100 μL lysis buffer (50 mM Tris pH 7.4, 40 mM NaCl, 1 mM EDTA, 0.5% Triton, 1.5 mM Na3VO4, 50 mM NaF, 10mM sodium pyrophosphate) with proteinase inhibitor (Roche, Vienna, Austria) on ice for 10 min. The protein concentration of lysis determined by BCA method. PI3 kinase activity was measured using PI3 kinase activity ELISA kit (K-1000s, Echelon Biosciences, Salt Lake City, UT, USA) according to the manufacture's protocol.

T7E1 assay and next-generation sequencing (NGS) for indels

Genomic DNA from BMDMs, TAMs and other cell populations from different organs with *E. coli* protoplast-derived NVs treatment were extracted following the manufacturer's instruction (TIANGEN). The genomic regions covering the nuclease target sites were amplified with specific primers**.** To examine the off-target effect of NVs, CRISPOR program (http://crispor.tefor.net/) was applied to screen nine potential sites matching the on-target gene in high similarity which may induce off-target effects, corresponding primers were also used for PCR amplification. PCR products of *Pik3cg* locus purified by gel extraction were further used for NGS by Sangon Biotech and T7E1 assay. 500 ng amplicons were denatured by heating and annealed to form heteroduplex DNA. The samples were further incubated with T7E1 (NEB, Ipswich, MA, USA) for 30 min at 37°C and analyzed by 2% agarose gel electrophoresis to determine the efficiency of genome targeting. Mutation frequencies were calculated based on the band intensities using Image J software and the following equation: mutation frequency (%) $=100\times [1-(1-\text{fraction cleaved})^{1/2}]$.

PCR assay

Total RNA from *E. coli*, protoplast-derived NVs, BMDMs, and primary TAMs was isolated using TRIzol Reagent and reverse-transcribed into cDNA using the cDNA reverse transcription kit (Vazyme Biotech, Nanjing, China). The relative mRNA levels of genes were examined by qRT-PCR assay by StepOne™ Real-Time PCR System (Thermo Fisher). β-actin was used as an internal control. To examine the nuclear-entering efficiency of sgRNA, nuclear components from BMDMs treated with NVs were extracted with a nuclear extraction kit (Solarbio) as the manufacturer suggested. The existence of sgRNA in the total cell and nucleus in macrophages was determined by PCR and following agarose gel electrophoresis. β-actin was used as an internal

control for total RNA and U6 was used as the internal control for nuclear RNA. For the detection of sgRNA and CpG-rich sequences in *E. coli* and protoplast-derived NVs, PCR and agarose gel electrophoresis were performed and 16sRNA was used as the internal control. The absolute quantitative of sgRNA in *E. coli* and *E. coli* protoplast-derived NVs were analyzed by qRT-PCR. Standard curves were determined from the CT values of serial diluted sgRNA, which was synthesized by Genepharma (Shanghai, China). According to the standard curves, the absolute copy numbers of sgRNA were calculated. The primer sequences are shown in Supplementary Data 10.

Transcriptome sequencing and bioinformatic analysis

Total RNA was extracted from the 4T1 tumor tissues of mice with sg*Pik3cg*-DHP/DGA-NVs or PBS treatment using TRIzol reagent according to the manufacturer's protocol. To avoid the contamination of genomic DNA, RNA samples were treated with RNase-free DNase. The following cDNA library construction and high-throughput sequencing was performed by Novogene (Beijing, China). Differentially expressed genes (DEGs) were identified by the following criteria: fold change ≥ 1.5 and P value < 0.05.

To exploring the main biological functions of DEGs from sg*Pik3cg*-DHP/DGA-NVs treated tumor**,** Kyoto Encyclopedia of Genes and Genomes (KEGG) pathways was analyzed by KOBAS 3.0 software (http://kobas.cbi.pku.edu.cn/kobas3/)**.** And P value < 0.05 was considered significant. Gene set enrichment analysis (GSEA) software was provided from the institute of Massachusetts Technology, normalized enrichment score (NES) was to quantify enrichment magnitude and false discovery rate (FDR) was to quantify statistical significance.

Flow Cytometry analysis

Cell suspensions from the blood, spleen, liver, tumor tissues or cell lines were filtered through Nylon cell strainers (70 μm, Falcon, USA), and red blood cells were lysed for primary cells suspensions. The cells were rinsed with PBS and further blocked with Fc-antibody diluting in PBS containing 1% BSA on ice for 10 min. For cell surface molecules staining, 1×10^6 cells were incubated with corresponding fluorescence-labeled antibodies for 30 min on ice and then washed with 1% BSA. 7-AAD staining was used for distinguishing live and dead cells. For intracellular staining, tumor leukocytes were first blocked with Fc-antibody, stained with antibodies against with cell surface molecules, fixated and permeabilized with BD Cytofix/Cytoperm solution (BD Bioscience, San Jose, NJ, USA), and then stained with antibodies against IFN-γ,

ki67 and Granzyme B. For IFN-γ staining, tumor leukocytes were firstly treated with Cell Activation Cocktail (with Brefeldin A) (Biolegend, San Diego, CA, USA) for 4 h before surface molecules staining. The Zombie Violet™ Fixable Viability Kit (Biolegend) was applied to distinguish live cells from dead cells. Flow cytometry was performed on an Attune NxT device (Thermo Fisher), and the data were analyzed with FlowJo v10.0 software (BD Biosciences, Ashland, OR, USA). The gating strategies used for flow cytometry analysis were shown in Supplementary Fig. 36-41. And the information of antibodies used was shown in Supplementary Data 11.

Western blotting assay

E. coli protoplast-derived NVs, BMDMs, and primary TAMs were lysed in RIPA Lysis Buffer containing protease inhibitor cocktail (Sigma, St. Louis, MO, USA) at 1:100 dilution and phosphatase inhibitors (Beyotime Biotechnology, Shanghai, China) at 1:50 dilution on ice for 30 min. Additionally, nuclear proteins from BMDMs treated with NVs were extracted with a nuclear protein extraction kit (Beyotime Biotechnology, Shanghai, China) as the manufacturer suggested. For Cas9, the total protein was isolated by 8% glycine SDS-PAGE, and the other proteins were isolated by 10% glycine SDS-PAGE and then transferred to the PVDF membrane. Proteins were detected by incubation with primary antibodies, washed and incubated with goat anti-rabbit HRP secondary antibodies (Jackson ImmunoResearch, West Grove, PA, USA, 1:2000). Primary antibodies directed against Akt, p-Akt, p65, p-p65, IRAK4, C/EBPβ, p-CEBPβ, STAT1, p-STAT1, STAT3, p-STAT3, p-TAK1, PI3Kγ and TAK1 were from Cell Signaling Technology (Boston, MA, USA), p-IRAK4 was from Abcam (Cambridge, UK). Normalization was performed by blotting the same membranes with an antibody against GAPDH for the total protein, histone (H3) for a nuclear component in mammalian cells, or RecA for *E. coli* and NVs. The information of antibodies used was shown in Supplementary Data 11.

Immunopathological staining

To observe the levels of macrophage galactose-type C-type lectin (MGL) expression and Cas9 protein nuclear import, M2-BMDMs and other cell lines were first fixed with 4% paraformaldehyde for 15 min, then treated with 0.3% Triton X-100 for 10 min (only for intracellular staining), blocked with PBS containing 3% BSA for 1 h and incubated with antibodies (MGL, F4/80 and Cas9) at 4 °C overnight. Corresponding fluorescence-labeled secondary antibodies were applied at room temperature for 45 min, followed by nuclear DAPI staining.

To examine MGL expression and phenotype identification of TAMs, TAM targeting ability of sg*Pik3cg*-DHP/DGA-NVs, and the phenotype identification of TAMs, cells or frozen tumor sections were stained with primary antibodies (F4/80, MGL, CD86, CD206) at 4 °C for 12 h followed with fluorescence-labeled secondary antibodies and DAPI nuclear staining. Cells and sections were imaged via a confocal microscope (LSM980, ZEISS, Baden-Württemberg, Germany).

To detect the IL-12/TNF-α/IL-10/TGF-β1 levels in tumor tissues, paraffin tumor sections were deparaffinized and hydrated, then sections were boiled in citrate buffer for 10 min for epitope retrieval and blocked with 5% BSA for 1 h. Then, tumor slides were incubated with primary antibodies at 4 °C overnight followed by biotinylated-conjugated corresponding secondary antibodies at room temperature for 45 min, washed with PBS, reacted with Peroxidase-labeled Streptavidin and finally re-dyed with hematoxylin. Immunohistochemical sections were photographed using a forward microscope (Nikon DS-Ri2, Tokyo, Japan). All staining experiments were performed three times with different samples. To exclude the possibility of background staining, samples treated with secondary antibodies alone were used as negative controls. IHC staining images were scored using the semiquantitative immunoreactive score (IRS) system by ImageJ image analysis software. The intensity of immunostaining was given a score of 0–3 (0, negative; 1, weak; 2, moderate; 3, strong), and the cell percentage with positive staining cells was given a score of 1–4 (1, 0–25%; 2, 26–50%; 3, 51–75%; and 4, 76–100%). Multiplication of the intensity and the percentage of marker-positive cells resulted in an IRS ranging from 0 to 12 for each sample. The information of antibodies used was shown in Supplementary Data 11.

The bio-safety assay of NVs

The determination of endotoxin content, CCK8, and hemolysis assay were performed to examine the toxicity of NVs. NVs were lysed by ultrasound sonication and the lysate were used to examine the endotoxin content (LPS) by ELISA kits (Fine Biotech, Wuhan, China) according to the manufacturer’s instruction. The cell viability of BMDMs treated with different amounts of NVs for 24 h was examined by CCK8 (DOJINDO, Kumamoto, Japan).

The hemolysis assay was performed as following described. Briefly, the freshly obtained mouse red blood cells (w/v=2%) were incubated with different amounts of NVs at 37°C for 4 h. The red blood cells incubated in double distilled water and PBS were designated as the positive and negative control, respectively. Then, the supernatant

was collected by centrifugation (300×g, 10 min) and the absorbance was measured at 540 nm by the micro plate reader (Thermo Fisher). The following formula was used to calculate the level of hemolysis: hemolysis ratio (%) = $(A_{sample}-A_{PBS}) / (A_{water}-A_{PBS}) \times 100\%$.

To examine the immunostimulatory effect of NVs, 1×10^{10} different types of NVs (sg*Pik3cg*-NVs, sg*Pik3cg*-DHP/DGA-NVs, sgControl-DHP/DGA-NVs or *E. coli* derived OMVs) were intravenously injected into 4T1 tumor-bearing mice. The serum was collected at 2 h and 24 h after NVs injection for ELISA assay (IL-6 and TNF-α). The whole blood was collected at 2 h and 24 h after NVs injection and the complete blood count test was conducted. Liver and spleen were also isolated, processed into cell suspensions, and analyzed using flow cytometry to assess changes in immune cell populations. Moreover, the survival rate of tumor bearing mice after NVs injection were calculated.

To examine the *in vivo* safety of NVs, the mice with NVs treatment were weighed every two days. The serum was collected for the measurement of ALT, AST, BUN, CR, and LDH by commercial kits (Jiancheng Bioengineering, Nanjing, China). Other organs (heart, liver, spleen, lung, and kidney) were harvested, sectioned, and stained with H&E for histological examination.

Supplementary Figures

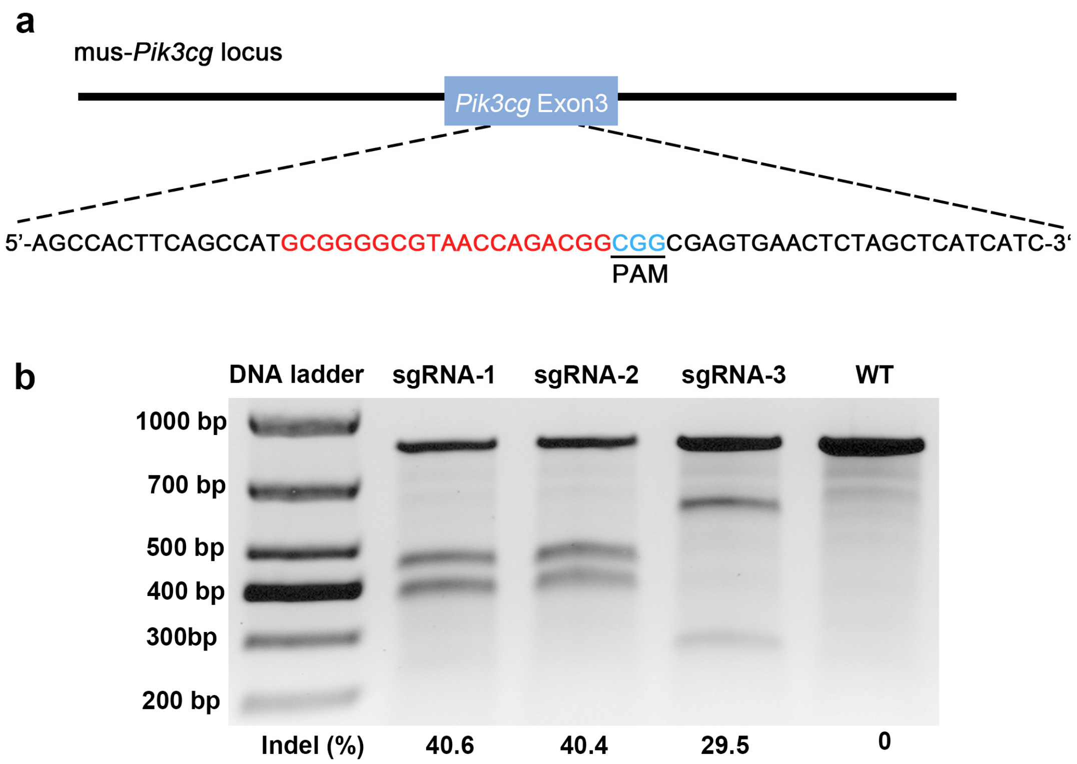

Name	Sequence
sgRNA-1	GCGGGGCGTAACCAGACGG(-)
sgRNA-2	GAGGTTTGGACGTTACCCAA(-)
sgRNA-3	GAGCCCTGGCCAGATCCACG(+)

Supplementary Fig. 1 Efficiency of CRISPR-Cas9 genome editing system for genome editing of *Pik3cg* examined by T7E1 analysis. a Schematic representation of the sgRNA targeting site in *Pik3cg*-locus. **b** A total of 5×10^5 RAW 264.7 cells were seeded in 6-well plates and transfected with 5 μg plasmid vectors carrying potential sgRNA sequences. After 48 hours, T7E1 analysis was performed to assess indel formation in RAW 264.7 cells. (+) and (-) represent the sense strand and antisense strand of genomic DNA. The experiment was repeated three times independently with similar results. Source data are provided as a Source Data file.

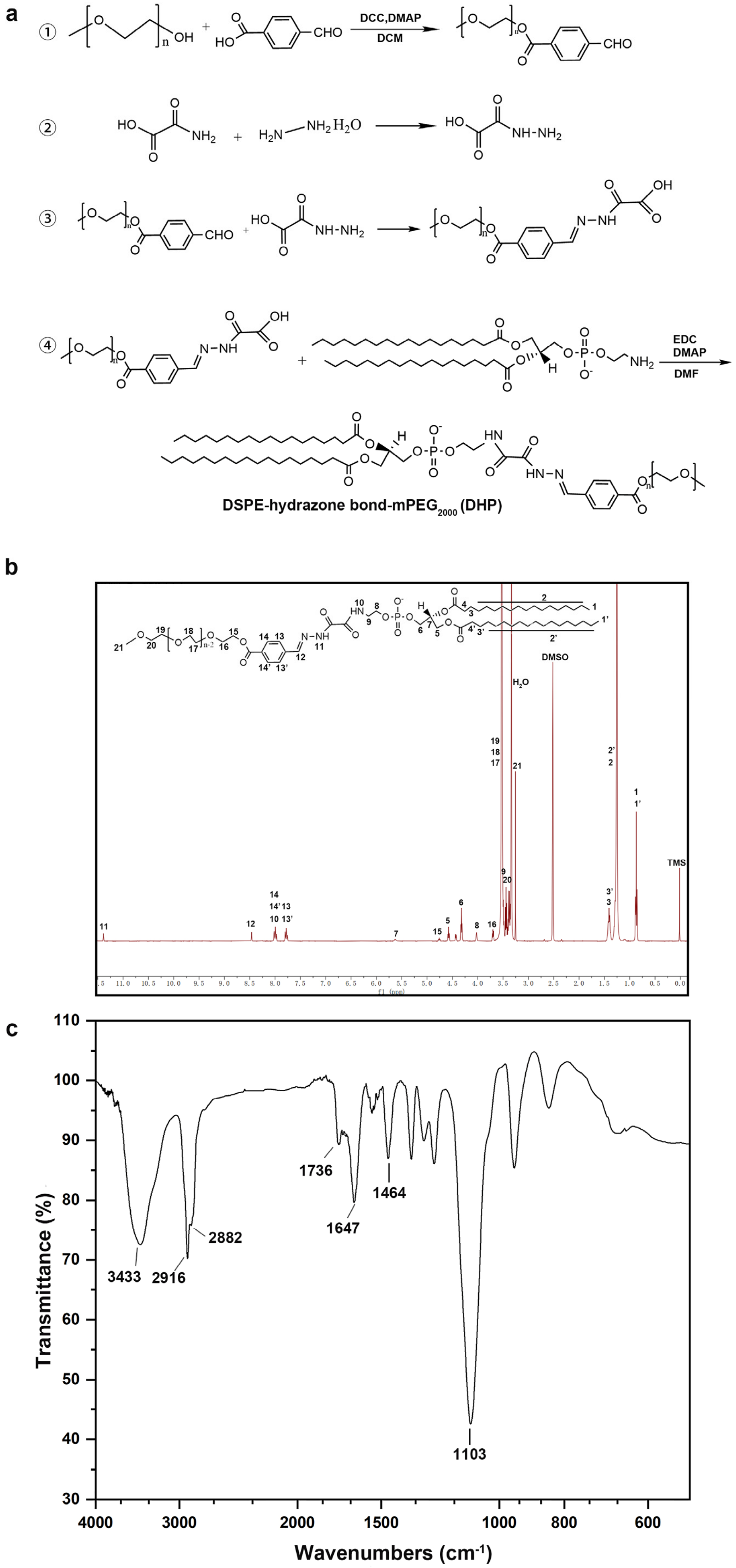

a
DCC,DMAP
DCM
EDC
DMAP
DMF
DSPE-hydrazone bond-mPEG2000 (DHP)
b
DMSO
H2O
TMS
c
Transmittance (%)
Wavenumbers (cm-1)
3433
2916
2882
1736
1647
1464
1103

Supplementary Fig. 2 Synthesis and characterization of DSPE-hydrazone bond-PEG_{2000} (DHP). a Schematic synthesis route of DHP. **b** ^{1}H NMR spectrum of DHP (400 MHz, DMSO). **c** FT-IR spectrum of DHP.

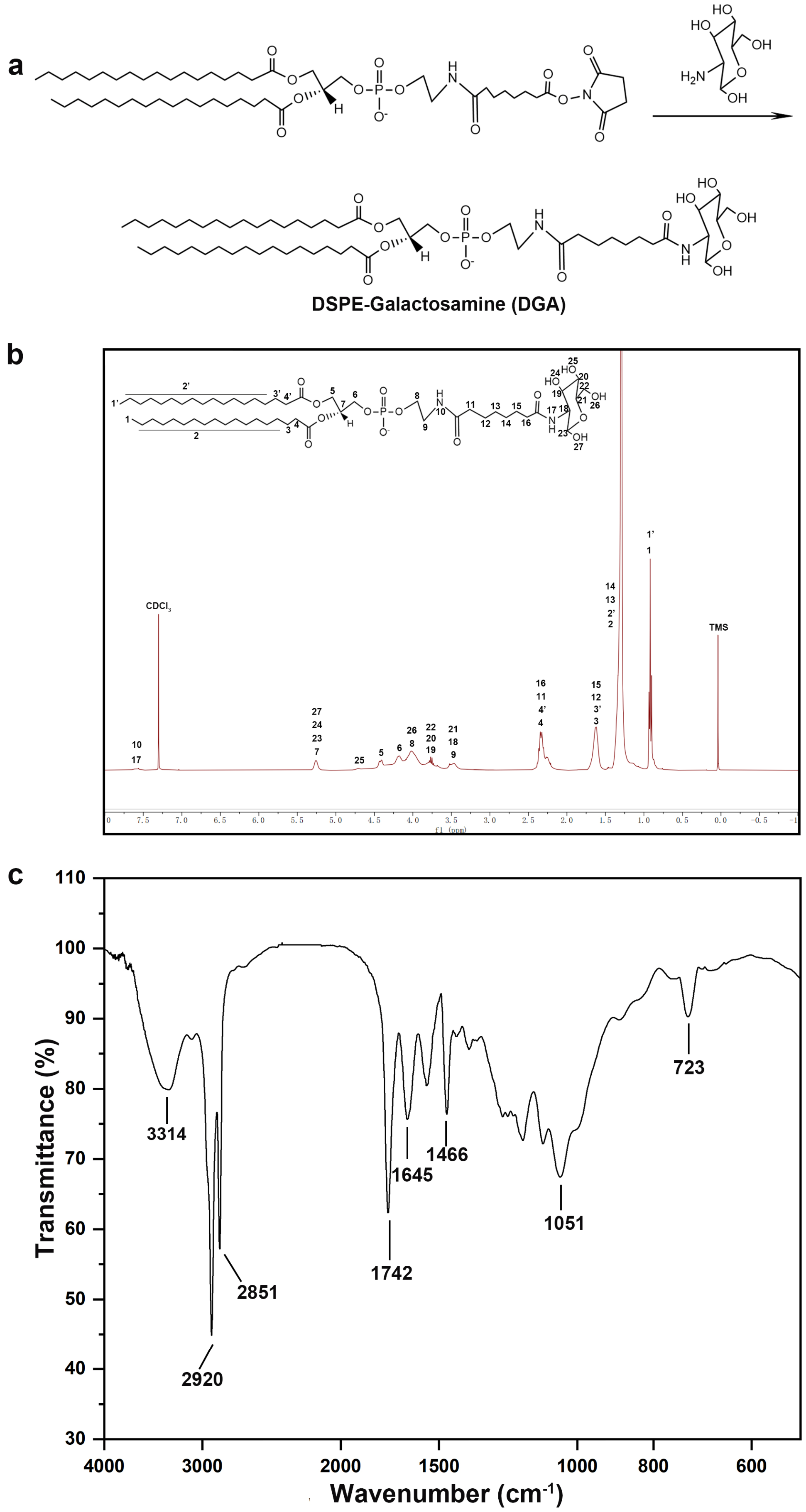
a
b
c
DSPE-Galactosamine (DGA)
CDCl3
TMS
f1 (ppm)
3314
2920
2851
1742
1645
1466
1051
723
Transmittance (%)
Wavenumber (cm-1)

Supplementary Fig. 3 Synthesis and characterization of DSPE-galactosamine (DGA). **a** Schematic synthesis route of DGA. **b** ^{1}H NMR spectrum of DGA (400 MHz, CDCl3). **c** FT-IR spectrum of DGA.

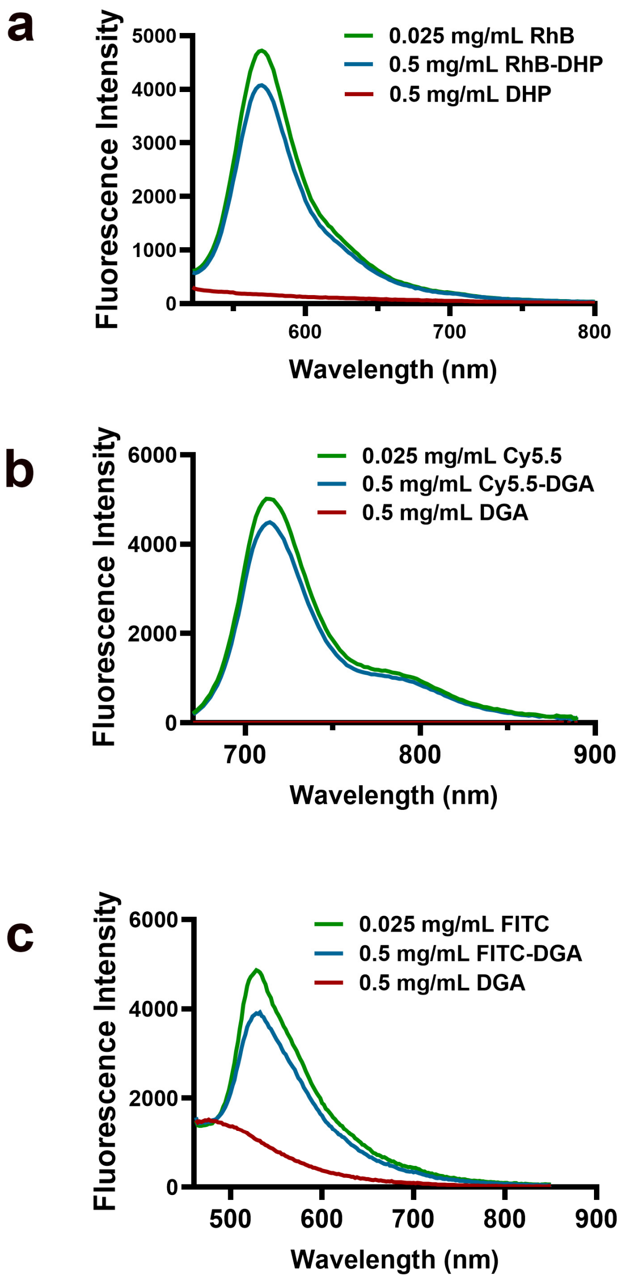

Supplementary Fig. 4 Fluorescence spectra of fluorescence-labeled DHP and DGA. **a** Fluorescence spectra of 50 μg of Rhodamine B (RhB)-labeled DHP in 100 μL of $CHCl_3$ with an emission wavelength (Em) of 480 nm and an excitation range (Ex) of 518-800 nm. **b** Fluorescence spectra of 50 μg of Cy 5.5-labeled DGA in 100 μL of $CHCl_3$, featuring an Em of 626 nm and an Ex range of 660-890 nm. **c** Fluorescence spectra of 50 μg of FITC-labeled DGA in 100 μL of $CHCl_3$, with an Em of 420 nm and an Ex range of 462-850 nm. The experiments for panels (**a**-**c**) were repeated three times independently with similar results. Source data are provided as a Source Data file.

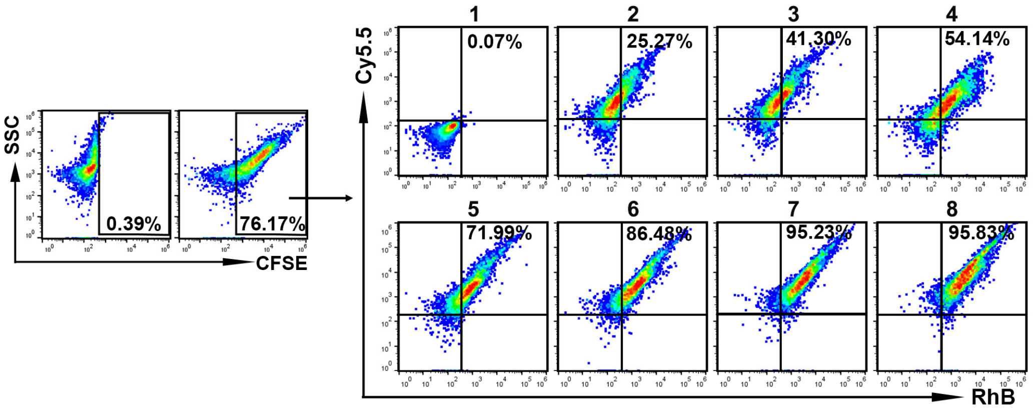

	RhB-DHP amount (μmol) per 1×10⁹ CFU *E.coli* derived protoplast	Cy5.5-DGA amount (μmol) per 1×10⁹ CFU *E.coli* derived protoplast
1	0	0
2	4.22	4.22
3	8.44	8.44
4	16.88	16.88
5	33.75	33.75
6	67.5	67.5
7	101.25	101.25
8	135	135

Supplementary Fig. 5 Decoration efficiency of RhB-DHP and Cy5.5-DGA for NVs. A total of 1×10^9 CFU *E. coli* derived protoplast was added with specified amounts of RhB-DHP and Cy5.5-DGA for physical extrusion. Flow cytometry scatter plots depicting $RhB^+Cy5.5^+$ NVs were shown. RhB-DHP and Cy5.5-DGA were added during the NVs preparation process. n = 3 biologically independent samples.

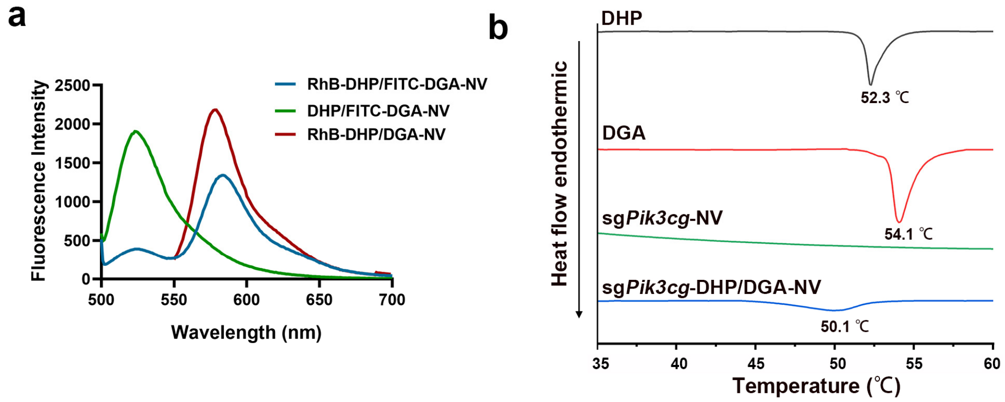

Supplementary Fig. 6 Floursence spectrum of sg*Pik3cg*-RhB-DHP/FITC-DGA-NVs and differential scanning calorimetry (DSC) spectrograms of protoplast-derived NVs decorated with DHP and DGA. **a** The fluorescence spectrum was obtained from 100 μL of PBS containing 5×10^{10} either RhB-DHP/FITC-DGA-NVs, RhB-DHP/DGA-NVs, or DHP/FITC-DGA-NVs. For DHP/FITC-DGA-NVs and RhB-DHP/FITC-DGA-NVs, the emission wavelength (Em) was set at 488 nm and the excitation (Ex) range was 500-700 nm. For RhB-DHP/DGA-NVs, the Em was 520 nm with an Ex range of 550-700 nm. **b** Effects of DHP and DGA on the phase transition temperature of NVs bilayer membranes. The experiments for panels (**a**-**b**) were repeated three times independently with similar results. Source data are provided as a Source Data file.

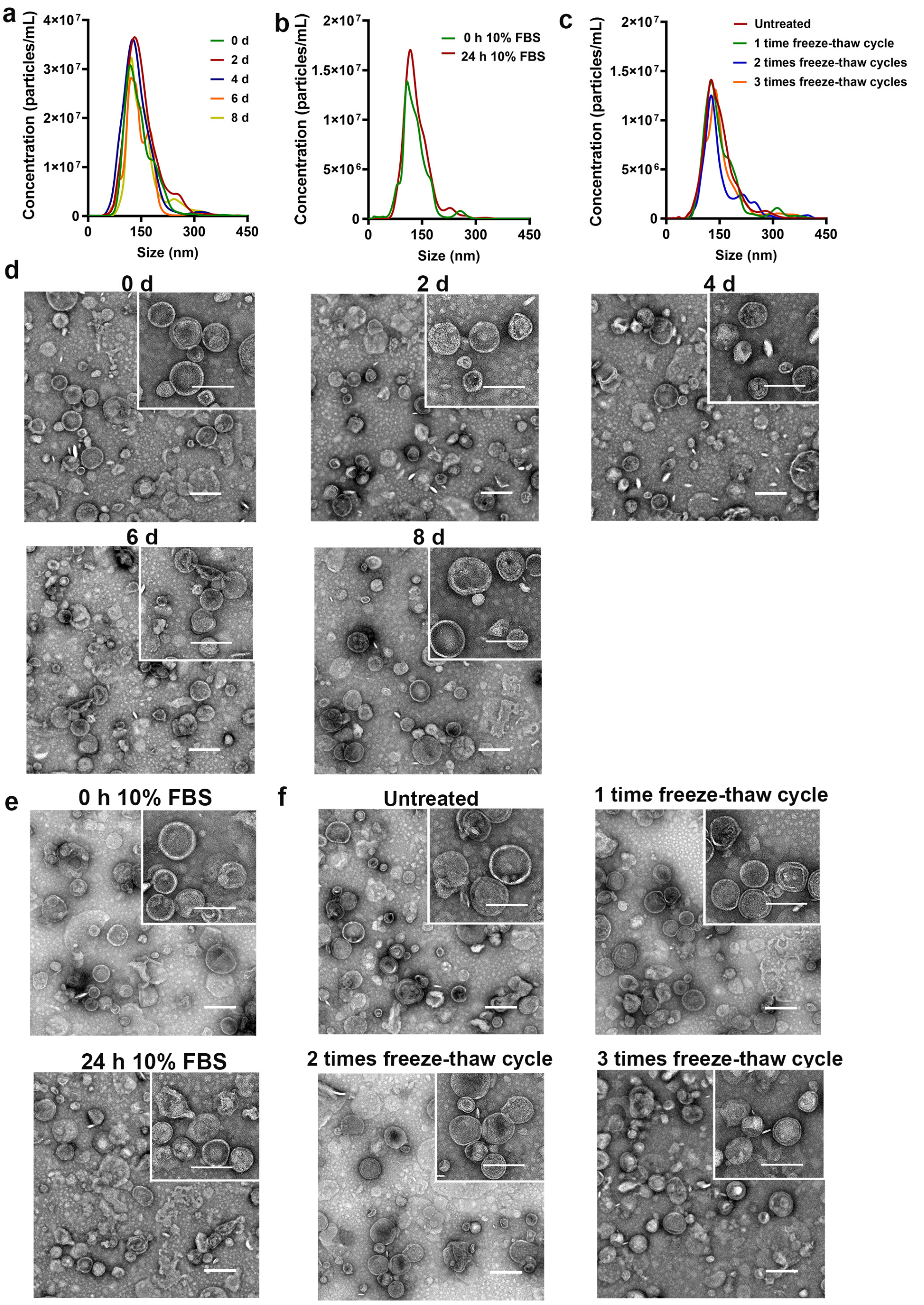

Supplementary Fig. 7 Characterization of sg*Pik3cg*-DHP/DGA-NVs under different treatments. a-c The average diameter of 1×10^{10} NVs in a 100 μL volume with different treatments (eight-day incubation in PBS with for s sampling every two, 10% FBS incubation, and multiple freeze-thaw cycles) was examined using Nanosight analysis. **d-f** The morphology of NVs was characterized using transmission electron microscopy (TEM). Scale bar = 200 nm. The experiments for panels (**a-f**) were repeated three times independently with similar results. Source data are provided as a Source Data file.

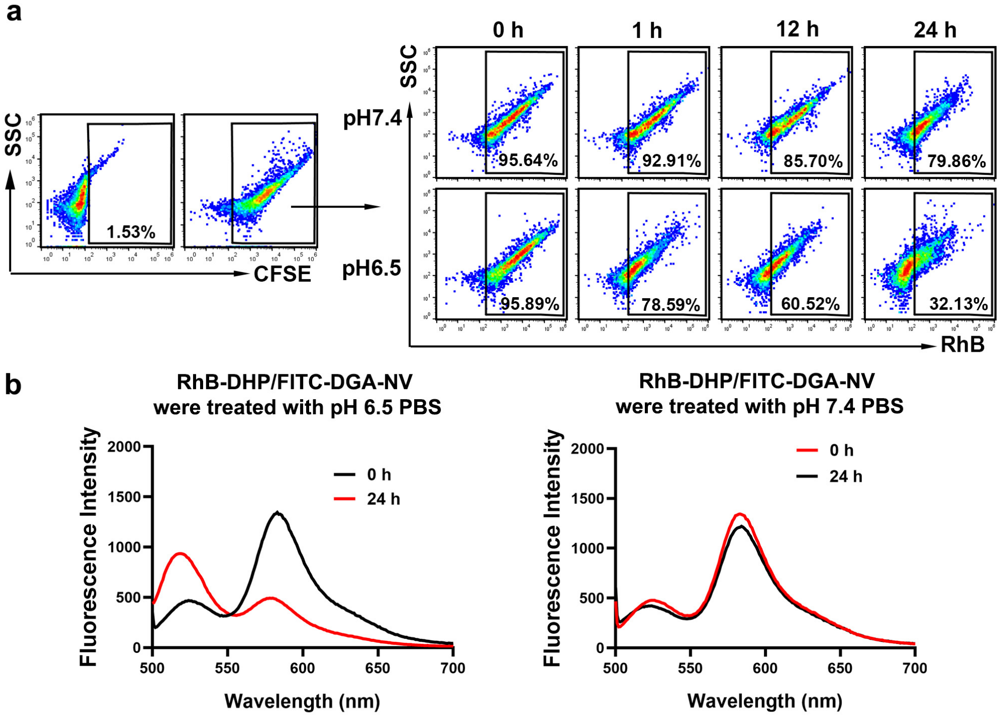

Supplementary Fig. 8 *In vitro* test of hydrazone linkage-breaking efficiency of DHP under the acidic environment. a Flow cytometry analysis was performed on sg*Pik3cg*-RhB-DHP/Cy5.5-DGA-NVs treated with PBS of different pH values at indicated time points. The representative images presented are from a sample size of n = 3. **b** The fluorescence spectrum of 5×10^{10} RhB-DHP/FITC-DGA-NVs was measured after treatment with 100 μL of PBS at different pH values. The excitation wavelength was set at 488 nm, with an emission scan ranges from 500 to 700 nm. The experiments were repeated three times independently with similar results. Source data are provided as a Source Data file.

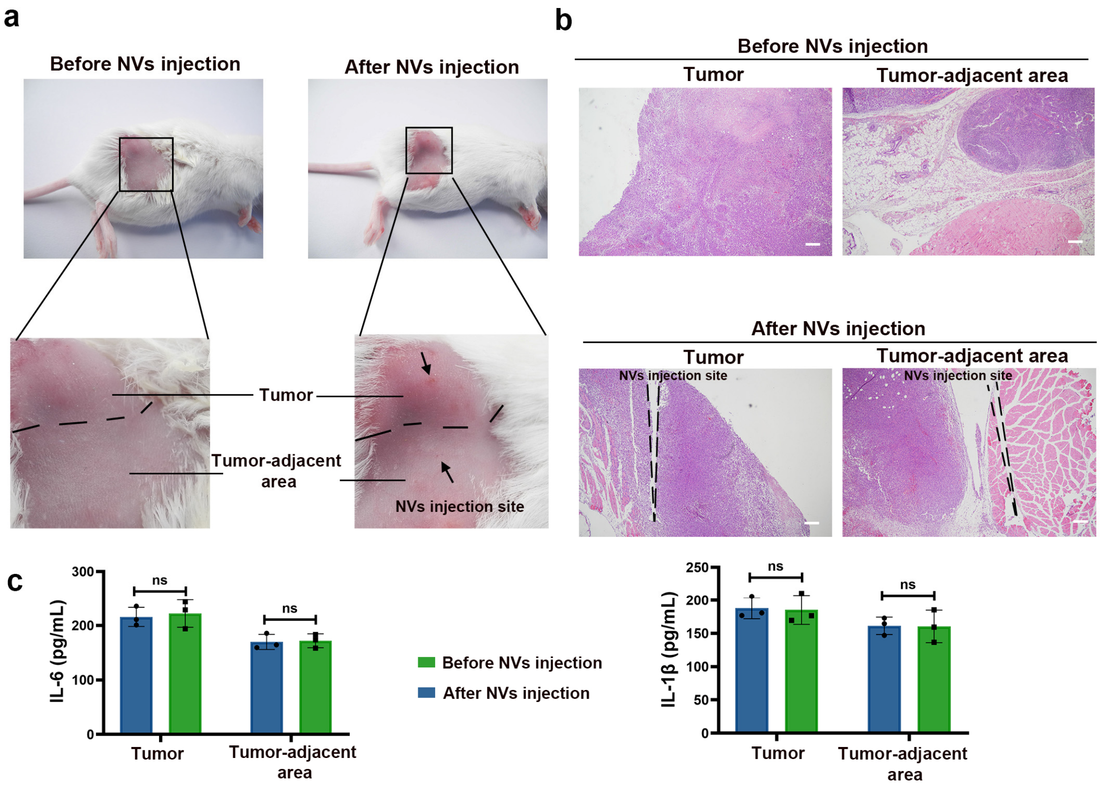

Supplementary Fig. 9 The evaluation of tissue damage and inflammation caused by *in situ* injection of sg*Pik3cg*-DHP/DGA-NVs. a The image of tumor area and tumor-adjacent area before and after *in situ* injection of 50 μL PBS containing 5×10^9 sg*Pik3cg*-DHP/DGA-NVs. The representative images presented are from a sample size of n = 3 mice. **b-c** H&E staining and cytokines detection of the injection site. Representative images are shown. Scale bar = 200 μm. n = 3 mice per group. Data are represented as mean ± SD. ns, no significant change. Statistical analyses were performed using unpaired student's t-test (two-tailed). The exact *P*-value and source data are provided as a Source Data file.

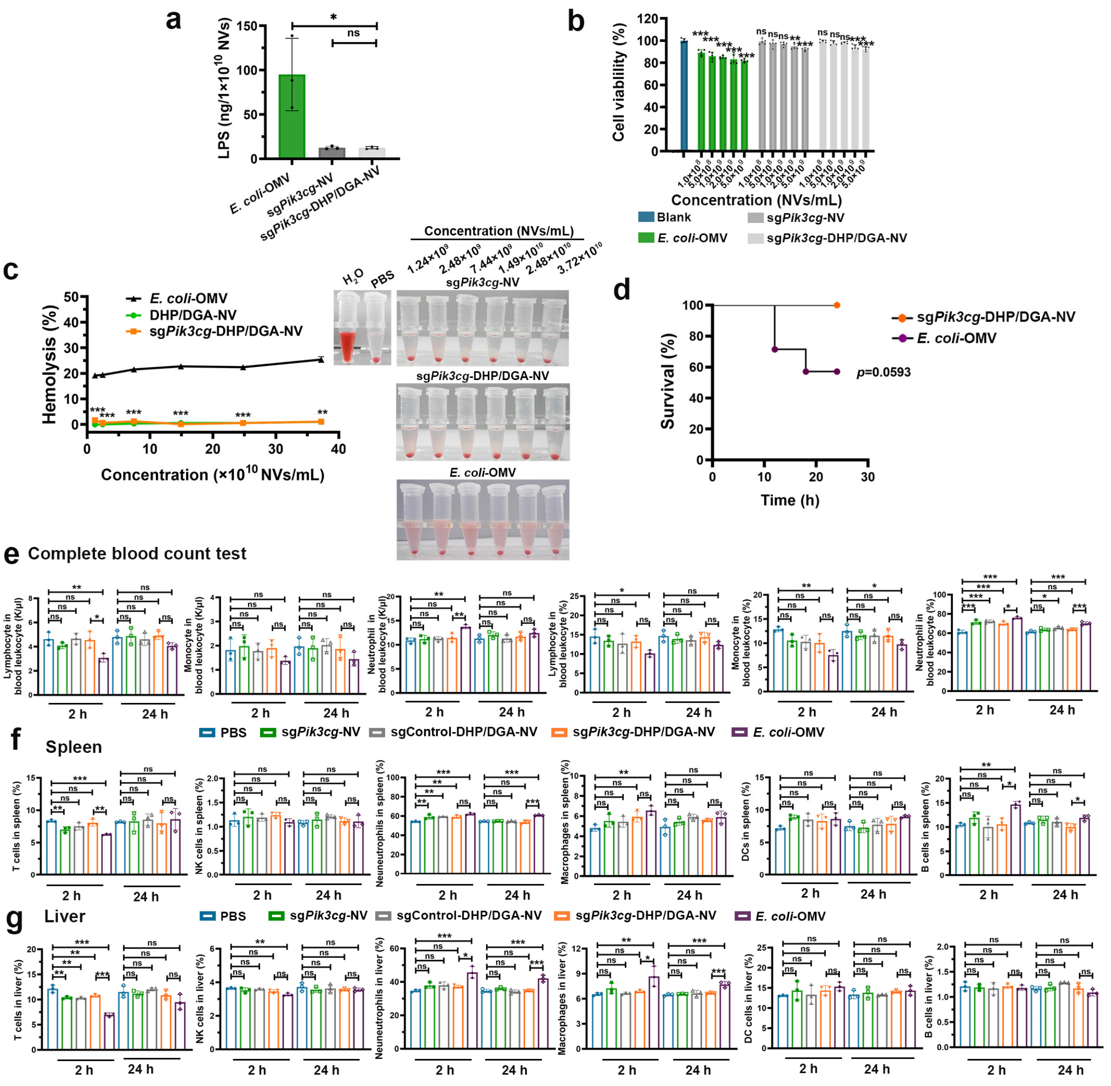

Supplementary Fig. 10 Bio-safety assessments after treatment with different NVs. **a** The LPS contents were measured in the lysate of 1×10^{10} *E. coli* protoplast derived-NVs or OMVs. n = 3 biologically independent samples. **b** A total of 5×10^{3} bone marrow-derived macrophages (BMDMs) in 96-well plates were treated with different types of NVs at the indicated concentrations for 24 h and the viability of BMDMs was determined using the CCK-8 assay. n = 5 biologically independent samples. $^{*}P < 0.05$ compared with blank group. **c** The hemolytic effects of *E. coli*-OMVs and the corresponding amount of NVs were examined using PBS containing 2% (w/v) mouse red blood cells. The statistical analysis was conducted between *E. coli*-OMVs and sg*Pik3cg*-DHP/DGA-NVs. n =3 biologically independent samples. **d** Survival rate of 4T1-bearing mice after in vein injections of 100 μL PBS containing 1×10^{10} different types of NVs (corresponding total protein amount: protoplast-derived NVs (94 μg in total protein amount); *E. coli*-OMVs (10 μg in total protein amount)). n = 7 mice per group. **e** The complete blood count test of 4T1-bearing mice at 2 h and 24 h after above mentioned NVs treatments. n = 3 mice per group. **f-g** Flow cytomety analysis of spleen and liver immune cell subsets in 4T1-bearing mice at 2 h and 24 h after above

mentioned NVs treatments. n = 3 mice per group. Data are represented as mean ± SD. Statistical analysis in panel **c** was performed using two-way ANOVA with Dunnett's multiple comparison test. The Log-rank (Mantel–Cox) test was used in panel **d**. Other statistical analyses were performed using one-way ANOVA with Dunnett's multiple comparison test except the FACS analysis of DC (24 h) and B cell (24 h) in panel **f** (Kruskal-Wallis test with Dunn's multiple comparisons test). *$P < 0.05$, **$P < 0.01$ and ***$P < 0.001$. ns, no significant change. The exact *P*-value and source data are provided as a Source Data file.

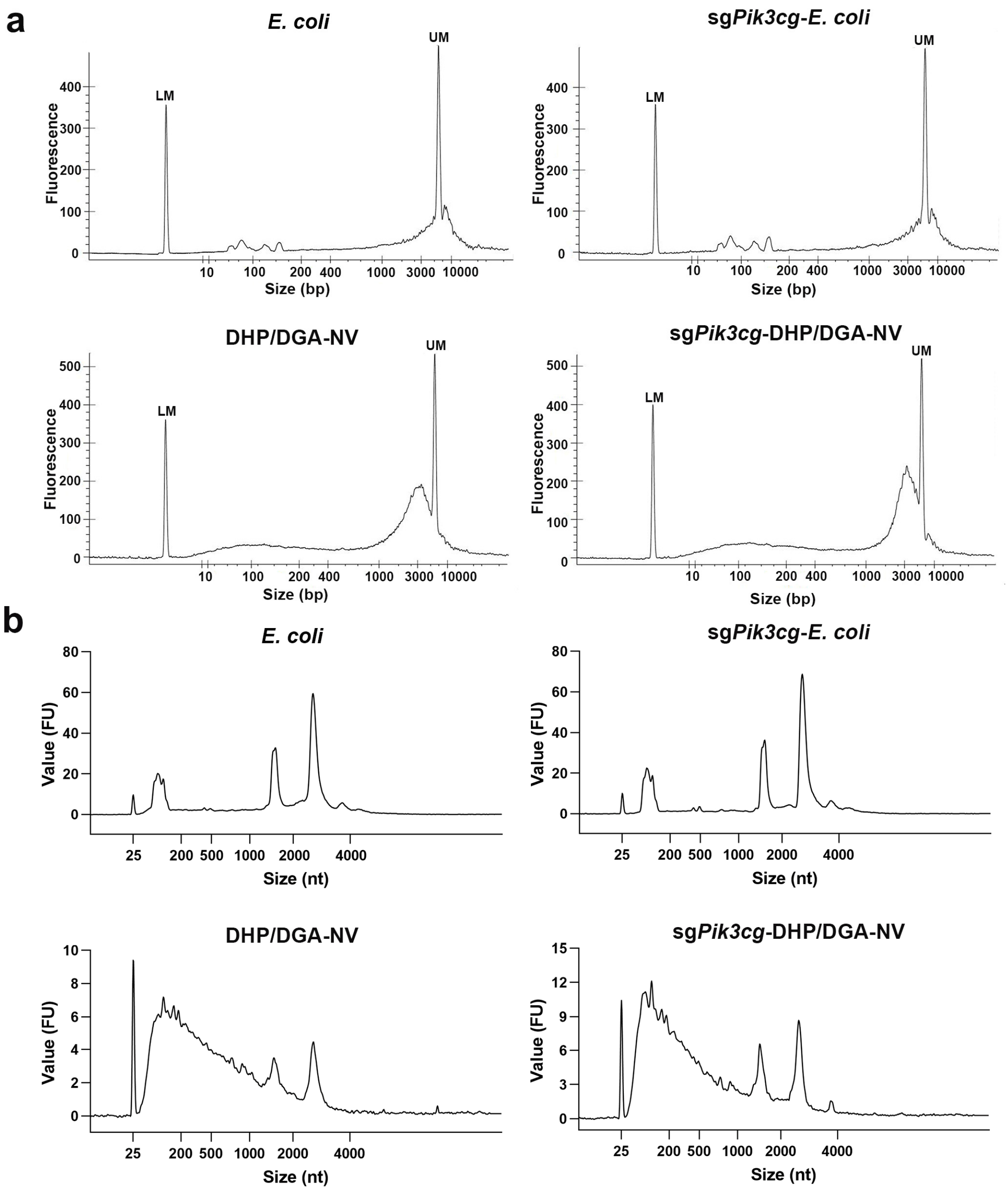

Supplementary Fig. 11 Electropherogram of nucleic acid components in *E. coli* and NVs. a The electropherogram summary of total DNA from *E. coli* and *E. coli* protoplast derived-NVs using LabChip bioanalyzer. **b** The electropherogram summary of total RNA from *E. coli* and NVs using Angilent 2100 bioanalyzer. The experiments for panels (**a**-**b**) were repeated three times independently with similar results.

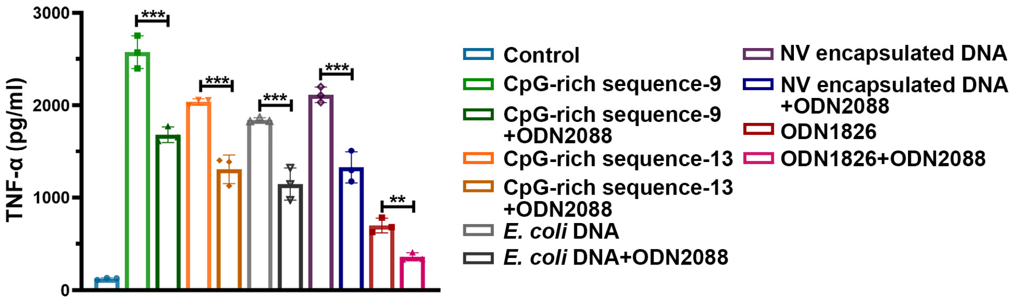

Supplementary Fig. 12 Immunostimulatory effects of CpG-rich genomic DNA sequences from *E. coli*. 1×10^6 BMDMs were treated with 10 μg/mL PCR amplicons of CpG-rich sequences localized in *E. coli* DNA, 500 ng/mL *E. coli* DNA, 500 ng/mL *E. coli* protoplast derived-NVs encapsulated DNA, or 10 μmol/L ODN1826 (TLR9 agonist) for 24 h. Under some circumstances, ODN2088 (TLR9 inhibitor, 10 μmol/L**)** and corresponding scramble control were added 30 min before the addition of above-mentioned nucleic acids and co-incubated for another 24 h. The supernatant was collected to determine TNF-α levels. n = 3 biologically independent samples. Data are represented as mean ± SD. Statistical analyses were performed using one-way ANOVA with Dunnett's multiple comparison test. $^{**}P < 0.01$ and $^{***}P < 0.001$. The exact *P*-value and source data are provided as a Source Data file.

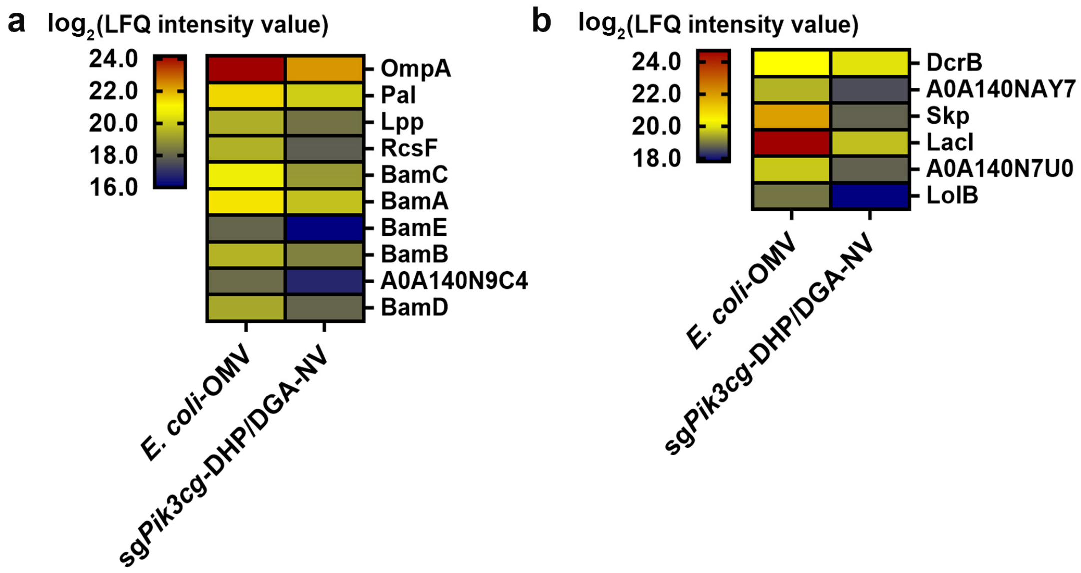

Supplementary Fig. 13 The comparison of outer membrane (a) and periplasm proteins (b) from *E. coli*-OMVs and *E. coli*-protoplast derived NVs. A total of 100 μg of protein from each of *E.coli*-OMVs and *E.coli*-protoplast derived NVs was subjected to LC-MS analysis. The abundance of protein across different samples was determined using the log2-transformed label-free quantification (LFQ) intensities and was represented in a heat map. All experiments for panels (**a**-**b**) were independently repeated three times, yielding consistent results. Source data are provided as a Source Data file.

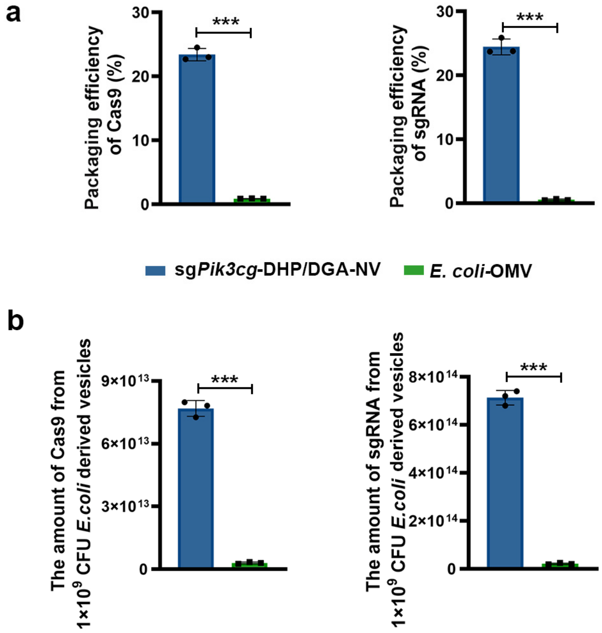

Supplementary Fig. 14 The quantifaication of Cas9 protein and sgRNA in sg*Pik3cg*-DHP/DGA-NVs and *E. coli*-OMVs. a The packaging efficiency of Cas9 and sgRNA in sg*Pik3cg*-DHP/DGA-NVs and OMVs. **b** The total amount of Cas9 and sgRNA in sg*Pik3cg*-DHP/DGA-NVs and OMVs obtained from 1×10^9 CFU *E. coli*. n = 3 biologically independent samples for panels (**a**-**b**). Data are represented as mean ± SD. *$P < 0.05$, **$P < 0.01$ and ***$P < 0.001$. Statistical analyses were performed using unpaired student's t test with Welch's correction (Two-tailed). The exact *P*-value and source data are provided as a Source Data file.

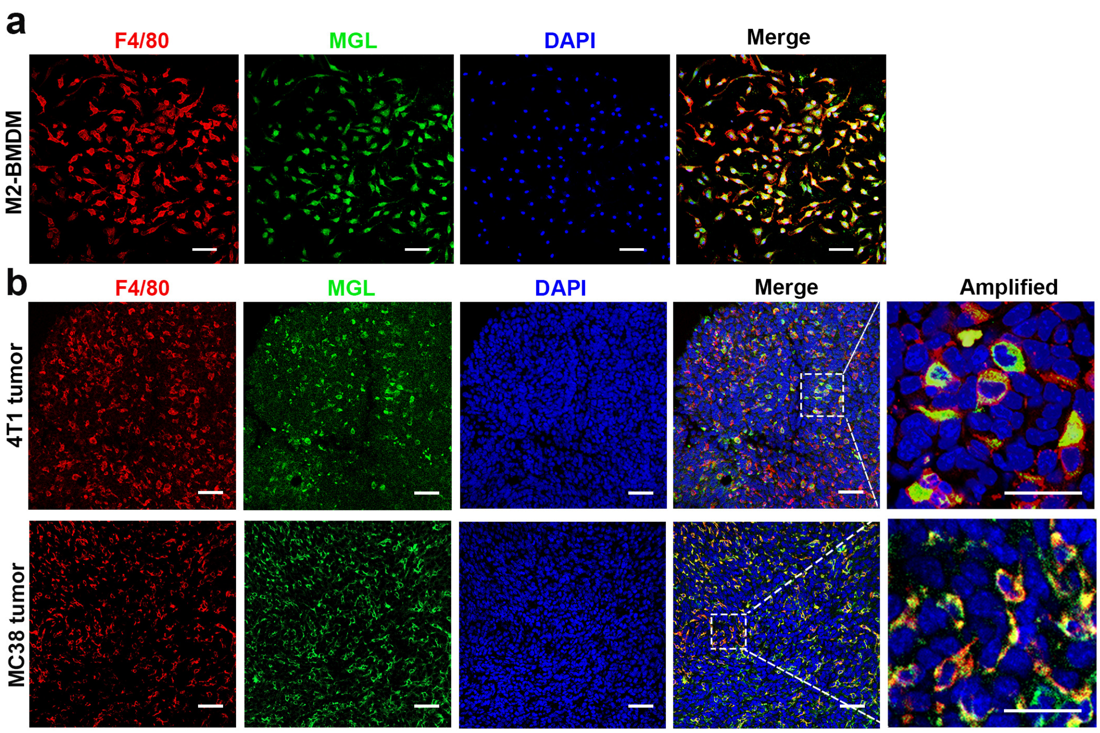

Supplementary Fig. 15 Detection of MGL in M2-BMDMs and TAMs in 4T1/MC38 tumor tissues of mice. a-b A total of 5×10^5 M2-BMDMs seeded in 24-well plates, as well as 4T1/MC38 tumor tissues from mice, were stained with antibodies targeting the macrophage marker F4/80 and macrophage galactose-type C-type lectin (MGL). These samples were then imaged. Red, F4/80; green, MGL; blue, DAPI nuclear staining. Scale bar = 50 μm; scale bar = 10 μm for amplified image. The experiments were repeated three times independently with similar results.

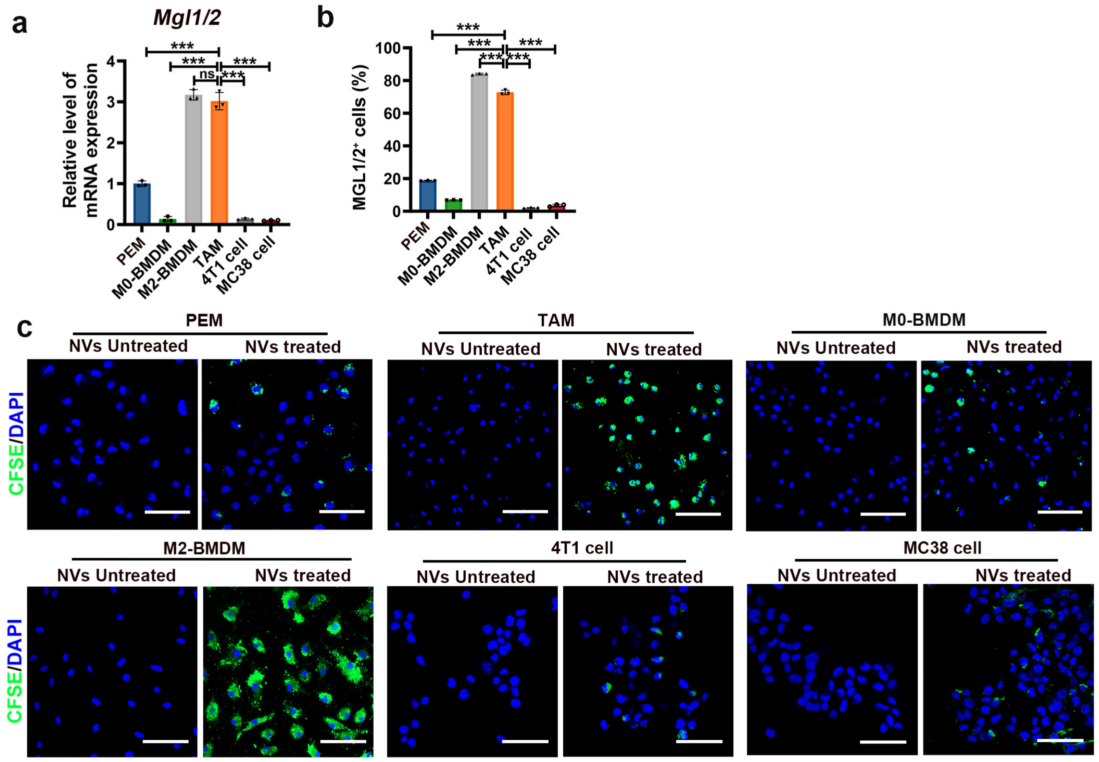

Supplementary Fig. 16 The efficiency evaluation of sg*Pik3cg*-DHP/DGA-NVs entry into cells with different MGL expression levels. **a** Relative *Mgl1/2* mRNA levels in different types of macrophages and tumor cells were determined by RT-qPCR. n = 3 biologically independent samples. **b** Flow cytometry was used to analyze the level of MGL1/2 in different types of macrophages and tumor cells. n = 3 biologically independent samples. **c** 5×10^5 different types of macrophages and tumor cells in 24-well plates were treated with 6×10^8 CFSE-sg*Pik3cg*-DGA-NVs for 3 h and applied for fluorescent microscopy. Green, CFSE labeled NVs; blue, DAPI nuclear staining. Scaled bar = 50 μm. The experiments were repeated three times independently with similar results. Data are represented as mean ± SD. Statistical analyses were performed using one-way ANOVA with Dunnett's multiple comparison test. *$P < 0.05$, **$P < 0.01$ and ***$P < 0.001$. ns, no significant change. The exact *P*-value and source data are provided as a Source Data file.

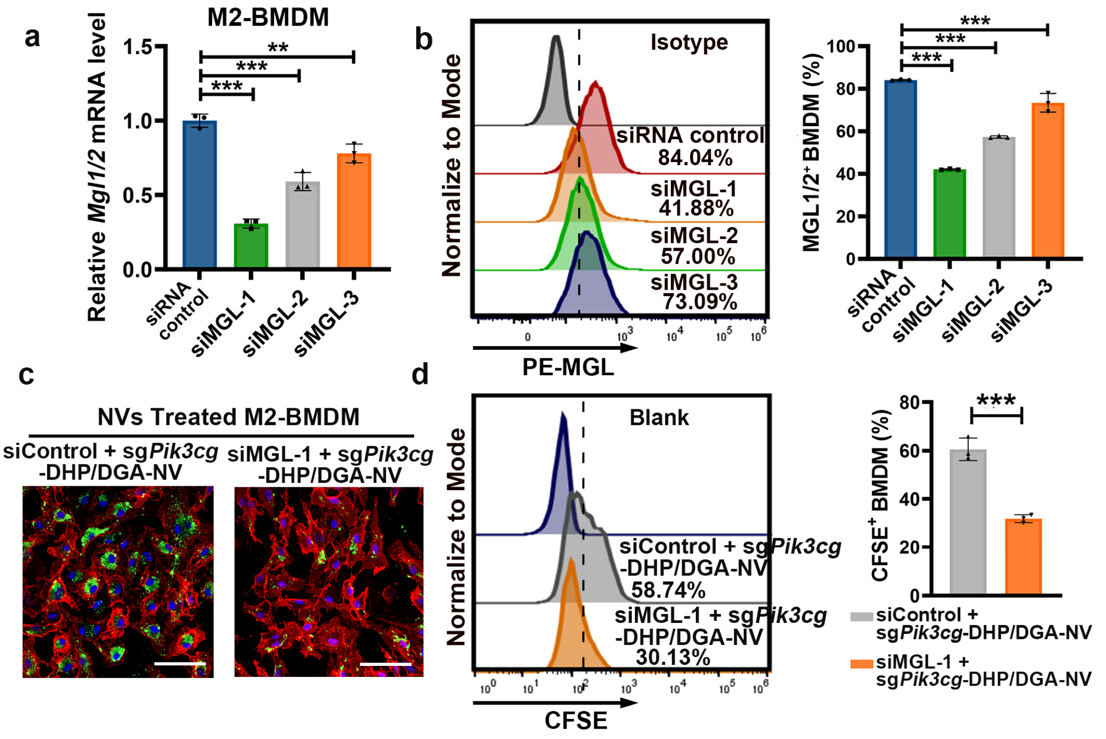

Supplementary Fig. 17 Cellular uptake of NVs by M2-BMDMs was denpendent on MGL-mediated endocytosis. a A total of 5×10^5 BMDMs were transfected with 200 pmol of siRNA targeting *Mgl1/2* for 48 h. The relative mRNA levels of *Mgl1/2* in M2-BMDMs were then examined using RT-qPCR 48 hours post-transfection. n = 3 biologically independent samples. **b** The ratios of MGL1/2$^+$ macrophages were determined by flow cytometry 48 h after the transfection of siRNAs targeting *Mgl*1/2. n = 3 biologically independent samples. **c-d** 5×10^5 M2-BMDMs were transfected with siRNA sequence 1 targeting *Mgl*1/2 (siMGL-1) for 48 h and subsequent treatment with 6×10^8 CFSE-sg*Pik3cg*-DGA-NVs for 3 h and applied for fluorescent microscopy and flow cytometry. Red, F4/80; green, CFSE labeled NVs; blue, DAPI nuclear staining. Scaled bar =50 μm. n = 3 biologically independent samples. Data are represented as mean ± SD. Statistical analyses were performed using one-way ANOVA with Dunnett's multiple comparison test except panel **d** (unpaired student's t-test (two-tailed)). *P < 0.05, **P < 0.01, ***P < 0.001. The exact P-value and source data are provided as a Source Data file.

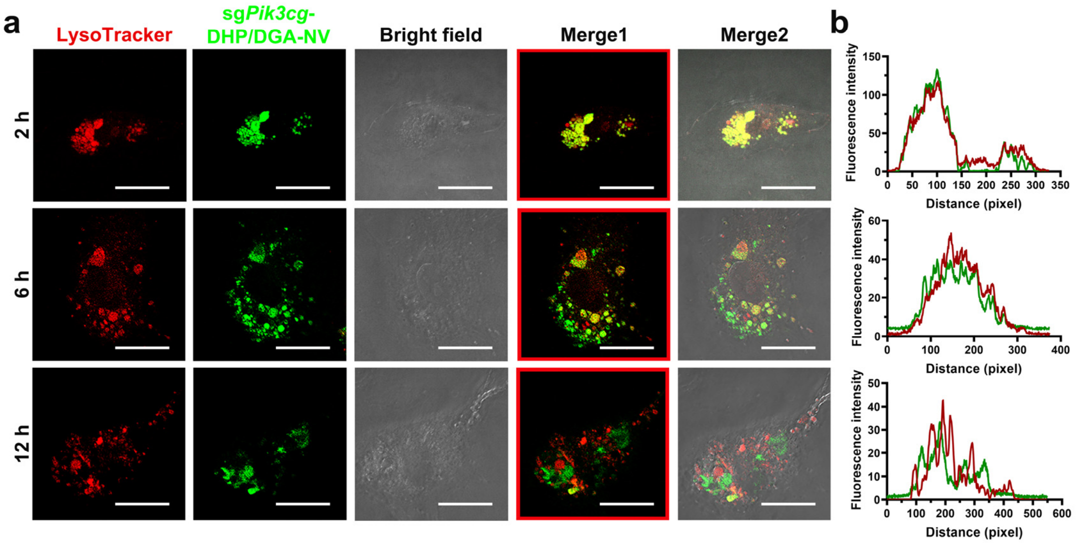

Supplementary Fig. 18 Subcellular localization of sg*Pik3cg*-DHP/DGA-NVs in M2-BMDMs. a A total of 5×10^5 M2-BMDMs were seeded in 24-well plates and treated with 6×10^8 CFSE-sg*Pik3cg*-DGA-NVs for 3 h. Subsequently, the cells were stained with LysoTracker Red at indicated time points and then imaged. **b** The scan profiles of fluorescence intensity in the red rectangle. Red, lysosomal dye; green, NVs. The experiments for panels (**a**-**b**) were repeated three times independently with similar results. Scale bar=25 μm. Source data are provided as a Source Data file.

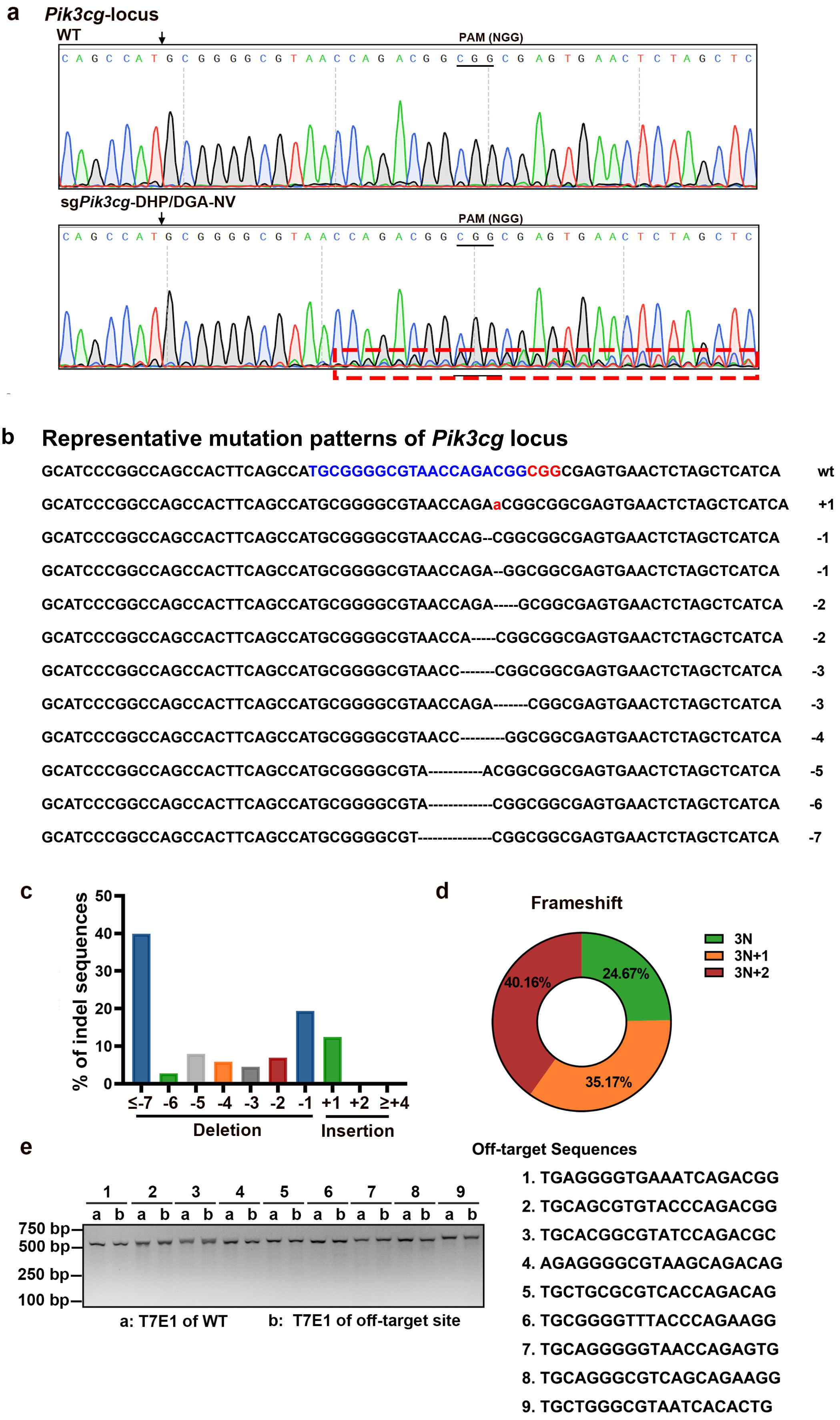
a
Pik3cg-locus
WT
PAM (NGG)
CAGCCATGCGGGGCGTAACCAGACGGCGGCGAGTGAACTCTAGCTC
sgPik3cg-DHP/DGA-NV
PAM (NGG)
CAGCCATGCGGGGCGTAACCAGACGGCGGCGAGTGAACTCTAGCTC
b
Representative mutation patterns of Pik3cg locus
GCATCCCGGCCAGCCACTTCAGCCATGCGGGGCGTAACCAGACGGCGGCGAGTGAACTCTAGCTCATCA wt
GCATCCCGGCCAGCCACTTCAGCCATGCGGGGCGTAACCAGAaCGGCGGCGAGTGAACTCTAGCTCATCA +1
GCATCCCGGCCAGCCACTTCAGCCATGCGGGGCGTAACCAG--CGGCGGCGAGTGAACTCTAGCTCATCA -1
GCATCCCGGCCAGCCACTTCAGCCATGCGGGGCGTAACCAGA--GGCGGCGAGTGAACTCTAGCTCATCA -1
GCATCCCGGCCAGCCACTTCAGCCATGCGGGGCGTAACCAGA-----GCGGCGAGTGAACTCTAGCTCATCA -2
GCATCCCGGCCAGCCACTTCAGCCATGCGGGGCGTAACCA-----CGGCGGCGAGTGAACTCTAGCTCATCA -2
GCATCCCGGCCAGCCACTTCAGCCATGCGGGGCGTAACC-------CGGCGGCGAGTGAACTCTAGCTCATCA -3
GCATCCCGGCCAGCCACTTCAGCCATGCGGGGCGTAACCAGA-------CGGCGAGTGAACTCTAGCTCATCA -3
GCATCCCGGCCAGCCACTTCAGCCATGCGGGGCGTAACC---------GGCGGCGAGTGAACTCTAGCTCATCA -4
GCATCCCGGCCAGCCACTTCAGCCATGCGGGGCGTA-----------ACGGCGGCGAGTGAACTCTAGCTCATCA -5
GCATCCCGGCCAGCCACTTCAGCCATGCGGGGCGTA-------------CGGCGGCGAGTGAACTCTAGCTCATCA -6
GCATCCCGGCCAGCCACTTCAGCCATGCGGGGCGT---------------CGGCGGCGAGTGAACTCTAGCTCATCA -7
c
% of indel sequences
50
40
30
20
10
0
≤-7 -6 -5 -4 -3 -2 -1 +1 +2 ≥+4
Deletion
Insertion
d
Frameshift
3N
3N+1
3N+2
24.67%
35.17%
40.16%
e
1 2 3 4 5 6 7 8 9
a b a b a b a b a b a b a b a b a b
750 bp
500 bp
250 bp
100 bp
a: T7E1 of WT
b: T7E1 of off-target site
Off-target Sequences
1. TGAGGGGTGAAATCAGACGG
2. TGCAGCGTGTACCCAGACGG
3. TGCACGGCGTATCCAGACGC
4. AGAGGGGCGTAAGCAGACAG
5. TGCTGCGCGTCACCAGACAG
6. TGCGGGGTTTACCCAGAAGG
7. TGCAGGGGGTAACCAGAGTG
8. TGCAGGGCGTCAGCAGAAGG
9. TGCTGGGCGTAATCACACTG

Supplementary Fig. 19 Evaluation of genome editing efficiency of *Pik3cg* in M2-BMDM treated with sg*Pik3cg*-DHP/DGA-NVs. A total of 1.5×10^6 M2-BMDMs in 6-well plates were treated with 1.8×10^9 sg*Pik3cg*-DHP/DGA-NVs, which had been pre-treated in pH 6.5 PBS for 6 h. This was followed by the replacement of the medium with fresh culture medium. The macrophages were then harvested 48 h after incubation with the NVs. **a** Sanger sequencing analysis of PCR amplicons in *Pik3cg* locus from M2-BMDMs. **b** Representative mutation patterns of *Pik3cg* locus in M2-BMDMs treated with sg*Pik3cg*-DHP/DGA-NVs were examined by NGS. **c** The proportion of indels in *Pik3cg* locus. **d** The frameshift pattern analysis in *Pik3cg* locus. **e** T7E1 analysis of the potential off-target effect in M2-BMDMs treated with sg*Pik3cg*-DHP/DGA-NVs. The experiments for panels (**a**-**e**) were repeated three times independently with similar results. Source data are provided as a Source Data file.

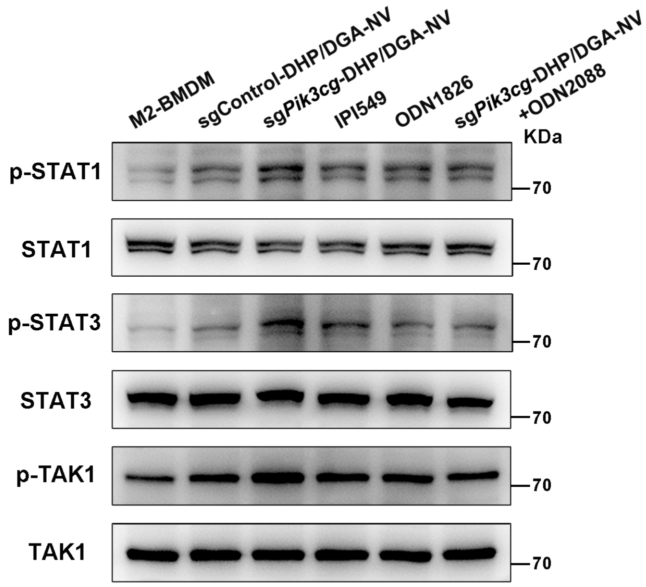

Supplementary Fig. 20 Western blotting of downstream proteins of IFN-γ, IL-6 and TNF-α signaling pathways. 1.5×10^6 M2-BMDMs in 6-well plates treated with NVs (1.8×10^9) for 6 h after which the medium was replaced with fresh medium for another 42 h, ODN1826 (10 μmol/L) or IPI549 (PI3Kγ inhibitor, 1 μmol/L) for 48 h, and then cells were harvested for western blotting to examine the level of p-STAT1, p-STAT3 and p-TAK1. The experiments were repeated three times independently with similar results. Source data are provided as a Source Data file.

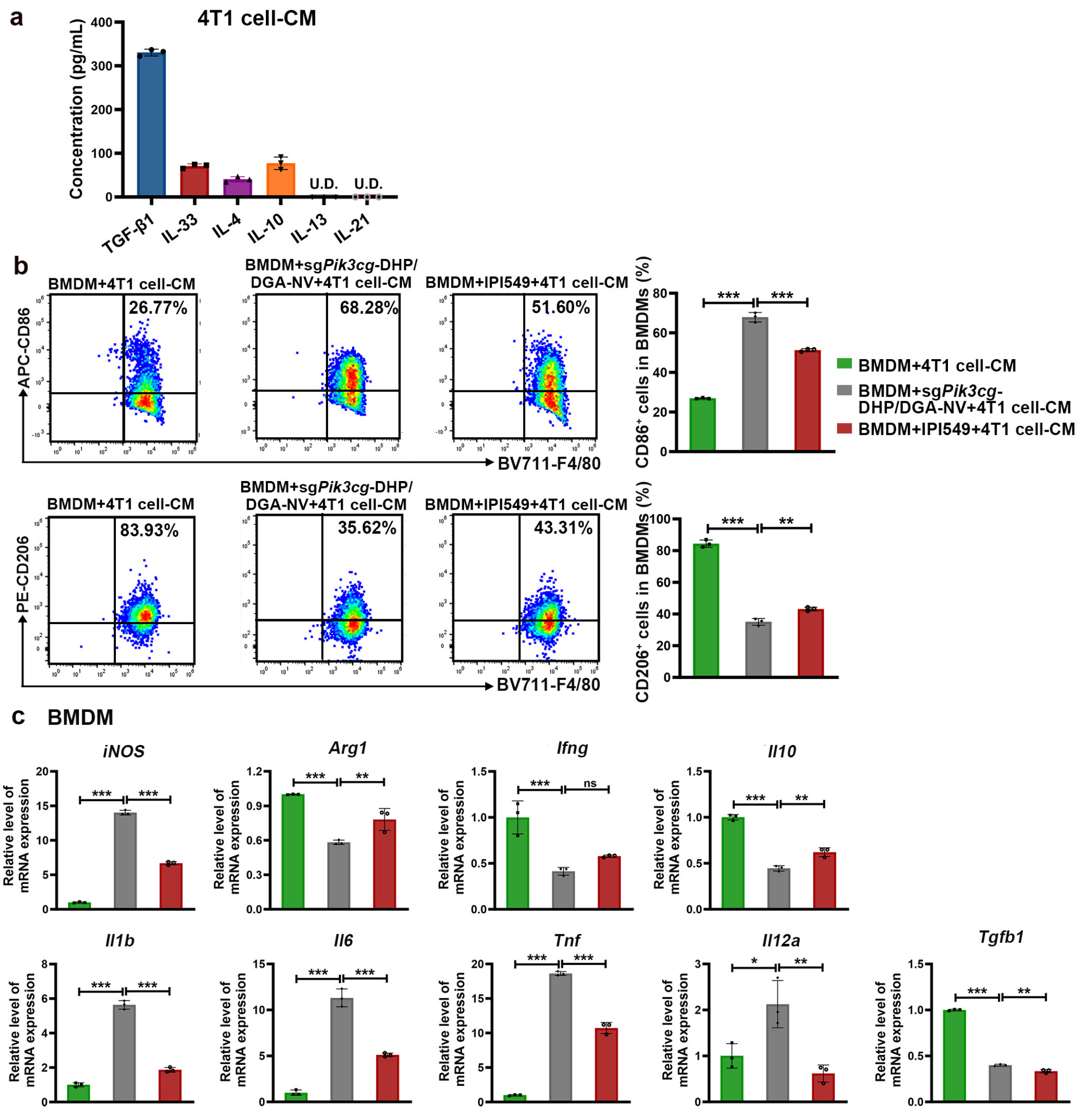

Supplementary Fig. 21 sg*Pik3cg*-DHP/DGA-NVs pre-treated BMDMs persistently maintain the immunostimulatory phenotype against 4T1 tumor cell conditioned medium. a The levels of M2-releated cytokines in the supernatant from 4T1 tumor cells were determined using ELISA. A total of 1×10^6 4T1 tumor cells were seeded in a 6-well plate, and the supernatant was collected 24 h post-seeding. n = 3 biologically independent samples. **b-c** 1.5×10^6 BMDMs in 6-well plates were treated with 1.8×10^9 sg*Pik3cg*-DHP/DGA-NVs for 6 h and further cultured in fresh DMEM medium for 18 h. Then, 4T1 tumor cell conditioned medium (4T1 cell-CM) was added to NVs pre-treated BMDMs for another 24 h incubation. Meanwhile, BMDMs were exposed to 1 μmol/L IPI549 treatment for 24 h, and subsequently, the 4T1 cell-CM containing an equivalent concentration of IPI549 was added for another 24 h. Flow cytometry analysis and qRT-PCR assay were applied to evaluate the influence of tumor cell-derived factors on macrophage polarization. n = 3 biologically independent samples for panels (**b**-**c**). Data are represented as mean ± SD. Statistical analyses were performed using one-way ANOVA with Dunnett's multiple comparison test. *$P < 0.05$, **$P < 0.01$ and ***$P <$ 0.001. U.D., undetected. The exact *P*-value and source data are provided as a Source Data file.

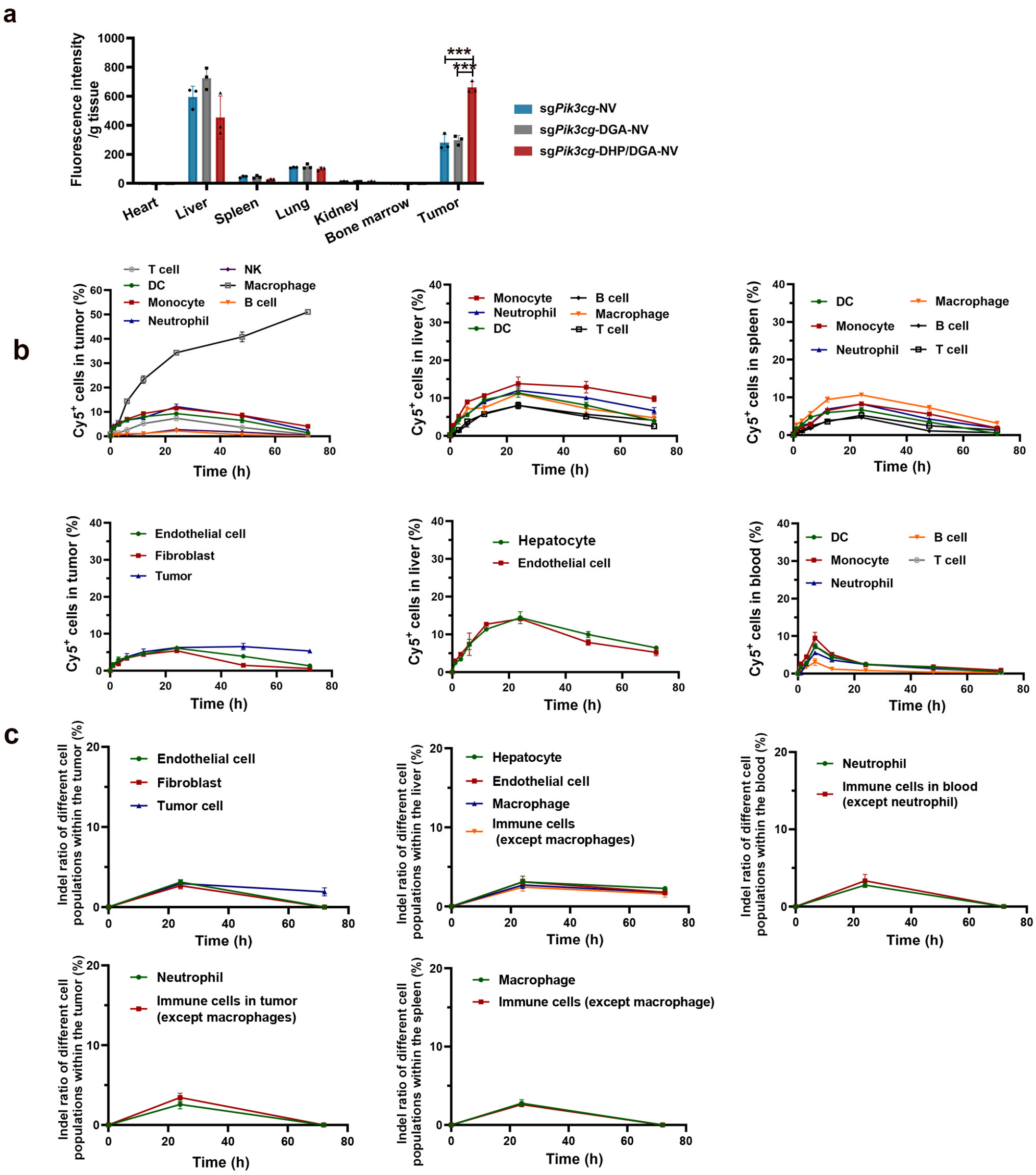

Supplementary Fig. 22 The bio-distribution of NVs and editing efficiency of different cell population from the tissues in 4T1 tumor bearing mice. a The Cy5 fluorescence intensity of different tissues from 4T1 tumor-bearing mice 72 h after in vein injection of different types of Cy5-NVs (dose: 1×10^{10} NVs per mice) (Em: 644 nm; Ex: 665 nm). $n = 3$ mice per group. *$P < 0.05$, **$P < 0.01$. **b** The ratios of Cy5-positive cells among cell populations from different organs of 4T1 tumor-bearing mice in vein injected with 1×10^{10} sg*Pik3cg*-DHP/DGA-NVs were examined by flow cytometry at indicated time points. n = 3 mice per time point. **c** Indel ratio of different cell population from different organs of 4T1 tumor-bearing mice in vein injected with 1×10^{10} sg*Pik3cg*-DHP/DGA-NVs were determined by T7E1 assay at indicated time points. n = 3 mice per time point. Data are represented as mean ± SD. Statistical analyses were performed using one-way ANOVA with Dunnett's multiple comparison test. ***$P < 0.001$. The exact *P*-value and source data are provided as a Source Data file.

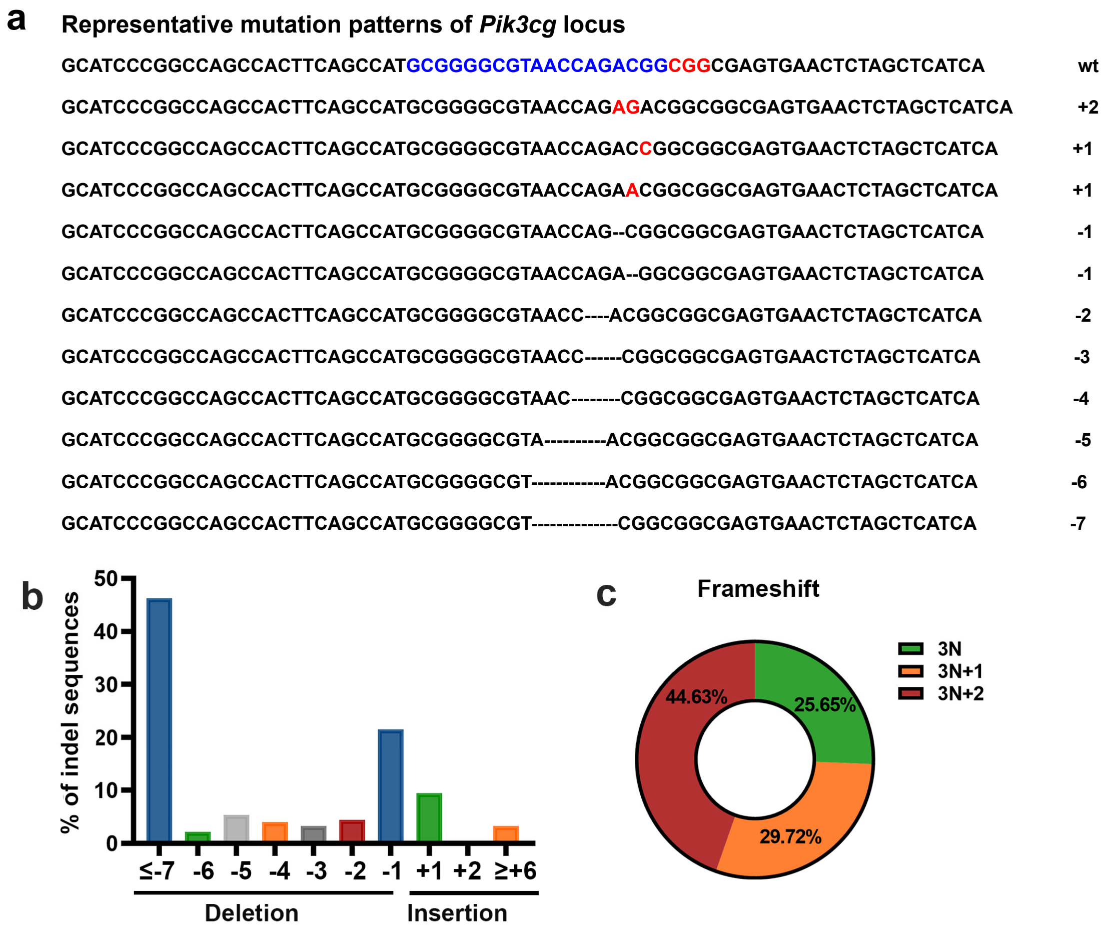

Supplementary Fig. 23 Evaluation of genome editing efficiency for *Pik3cg* in TAMs from 4T1 tumor-bearing mice after in vein injection of sg*Pik3cg*-DHP/DGA-NVs. **a** Representative mutation patterns of the *Pik3cg* locus in TAMs isolated from mice 72 h post to the treatment of 1×10^{10} sg*Pik3cg*-DHP/DGA-NVs were examined by NGS. **b** The proportion of indels in *Pik3cg* locus. **c** The frameshift pattern analysis in *Pik3cg* locus from TAMs. The experiments for panels (**a**-**c**) were repeated three times independently with similar results. Source data are provided as a Source Data file.

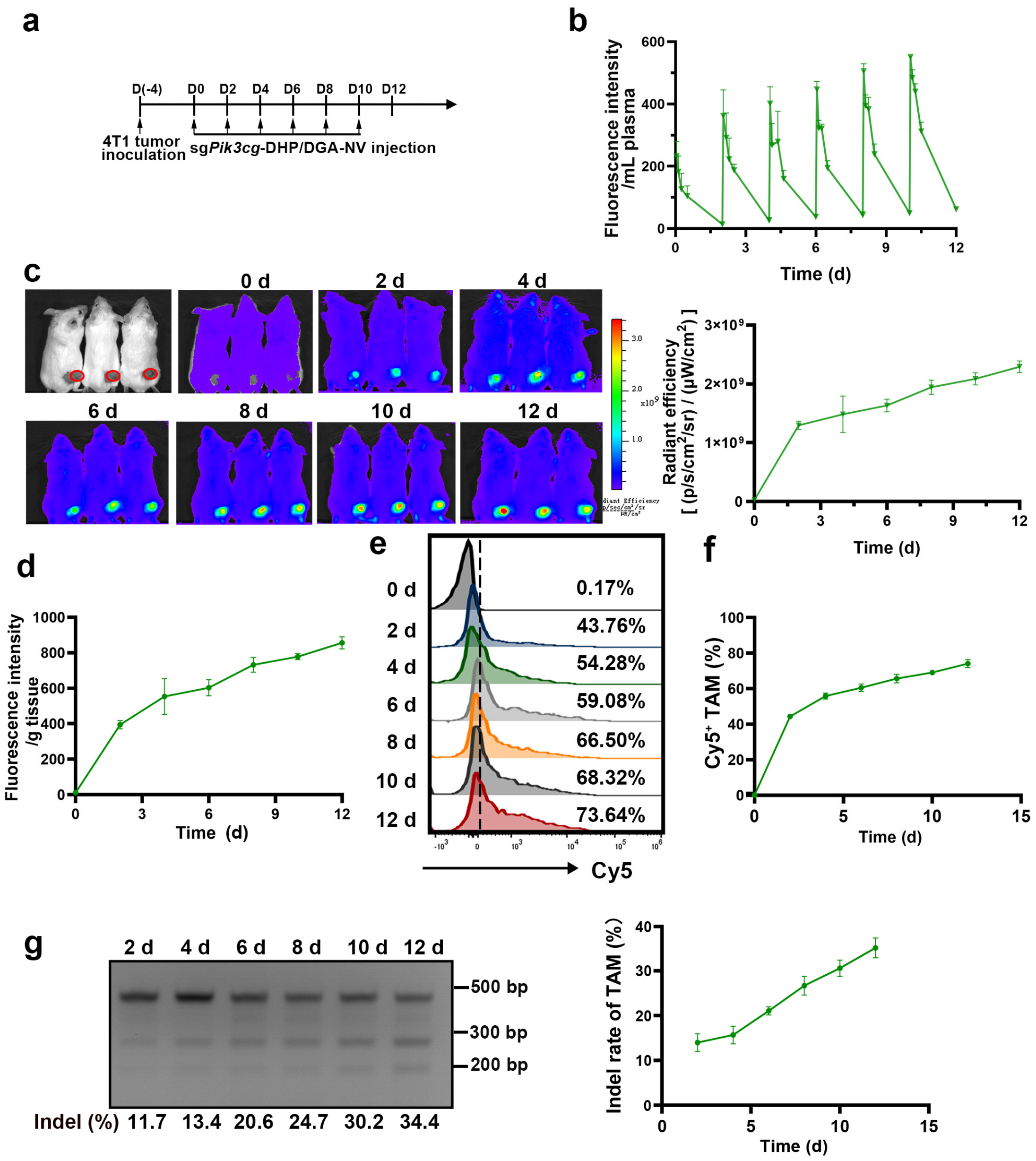

Supplementary Fig. 24 The accumulation of sg*Pik3cg*-DHP/DGA-NVs in tumor tissue and TAMs with the repeated administration. **a** Schematic illustration of intravenous injection of Cy5-labled sg*Pik3cg*-DHP/DGA-NVs (dose: 1×10^{10} NVs per mice, every two days for 6 times) into 4T1 tumor-bearing mice. **b** The Cy5 fluorescence intensity in the plasma from mice with repeated injection of Cy5-NVs was determined by microplate reader (Em: 644 nm; Ex: 665 nm). n = 3 mice per time point. **c** Fluorescence images of the 4T1 tumor-bearing mice and fluorescence quantitative analysis of the tumor site (red circle) at indicated time points after repeated injection of Cy5-NVs. n = 3 mice. **d** The Cy5 fluorescence density of tumor tissues from mice with repeated injection of Cy5-NVs was determined by microplate reader (Em: 644 nm; Ex: 665 nm) n = 3 mice. **e-f** Flow cytometry was used to analyze the ratio of $Cy5^+$ TAMs from mice after NVs injection at indicated time points. n = 3 mice per time point. **g** T7E1 analysis for indel formation in TAMs from mice at different time points after treatment with NVs. A representative image is shown. n = 3 mice per time point. Data are represented as mean ± SD. Source data are provided as a Source Data file.

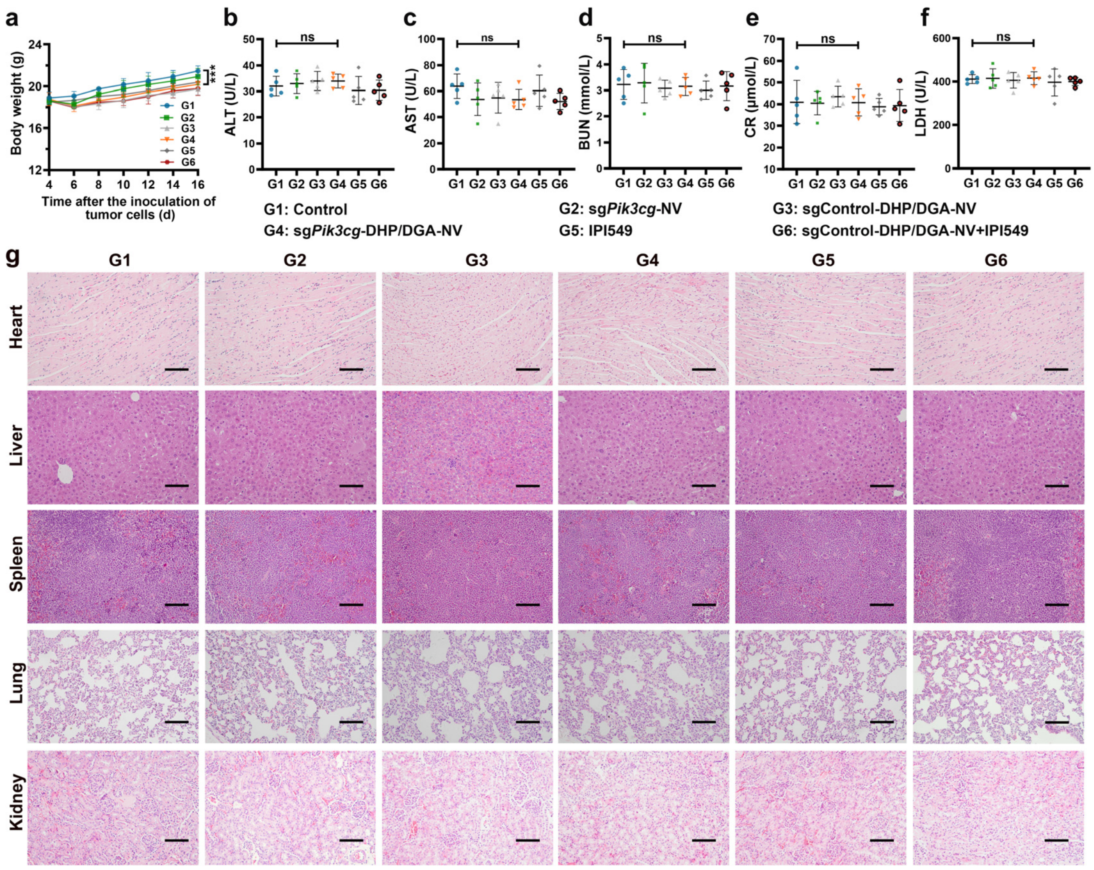

Supplementary Fig. 25 Safety assay in 4T1 tumor-bearing mice after treatment with different NVs. a Body weight curves of 4T1 tumor-bearing mice with an in-vein injection of NVs (1×10^{10} NVs every two days) or intragastric administration of IPI549 (15 mg/kg every day). Statistical analyses were performed using two-way ANOVA with Dunnett's multiple comparison test. **b-f** The examination of blood biochemical indexes from 4T1 tumor-bearing mice with NVs and IPI549 treatment on day 16 post to the inoculation of tumor cells. Statistical analyses were performed using one-way ANOVA with Dunnett's multiple comparison test. **g** Resprentative H&E staining of tissue sections harvested from heart, liver, spleen, lung and kidney of mice, conducted on day 16 following the inoculation of tumor cells, after treatment with different NVs and IPI549. Scale bar=100 μm. n = 5 mice per group for panels (**a-g**). Data are represented as mean ± SD. *$P < 0.05$ and **$P < 0.01$. ns, no significant change. The exact P-value and source data are provided as a Source Data file.

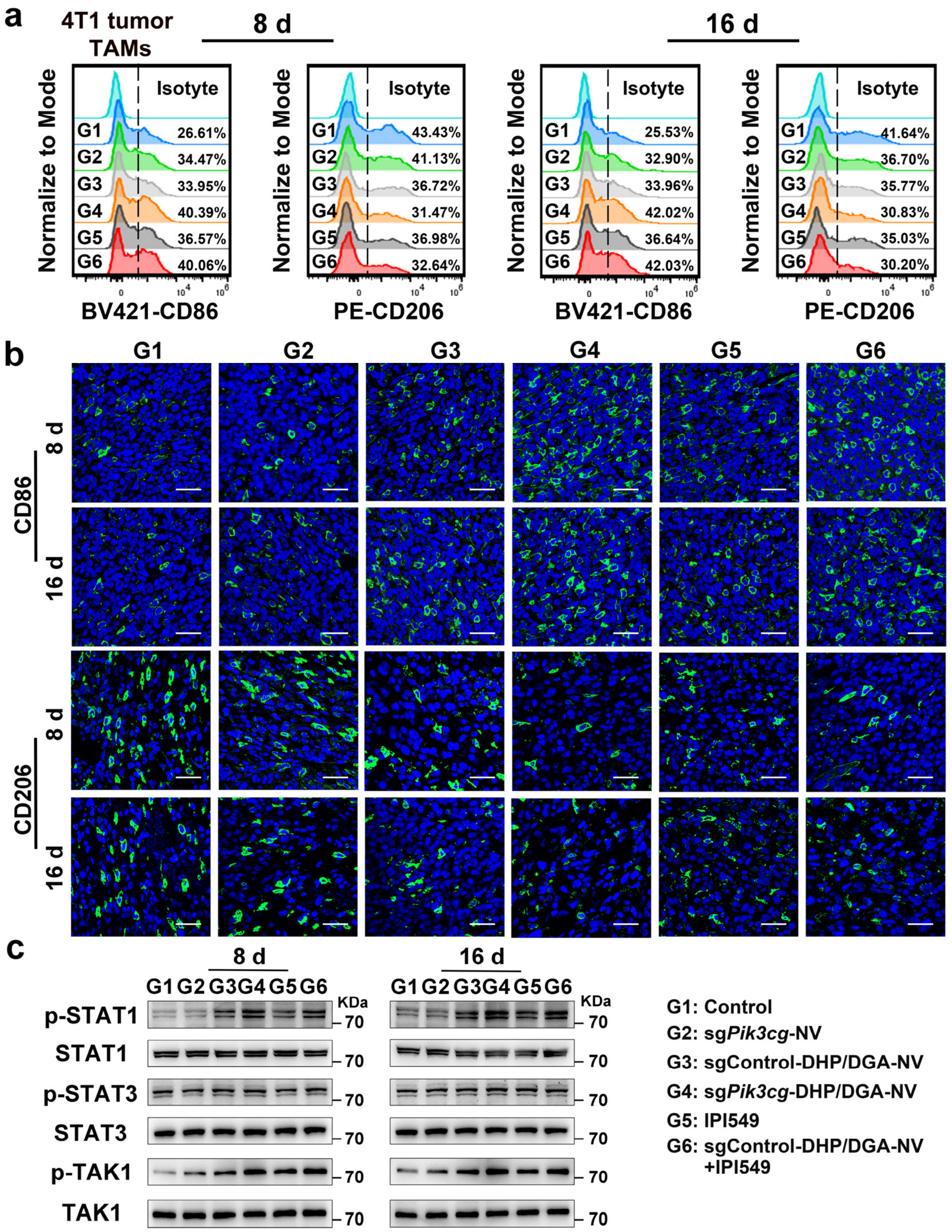

Supplementary Fig. 26 sg*Pik3cg*-DHP/DGA-NVs treatment repolarized TAMs in 4T1 tumor-bearing mice. a-b 4T1 tumor-bearing mice received treatments through intravenous injections of NVs at a dose of 1×10^{10} NVs every two days, or through daily intragastric administration of IPI549 at a dosage of 15 mg/kg. The effects of sg*Pik3cg*-DHP/DGA-NVs on TAMs phenotype (CD86 and CD206) from 4T1 tumor-bearing mice were identified by flow cytometry analysis and immunofluorescence staining at 8 days and 16 days post to the inoculation of tumor cells. Green, CD86/CD206; blue, DAPI nuclear staining. Scaled bar = 50 μm. Representative images are shown. n = 5 mice for each time point. **c** The levels of IFN-γ, IL-6 and TNF-α pathways related molecules (p-STAT1, p-STAT3 and p-TAK1) in TAMs isolated from mice with above mentioned treatments were detected by western blotting. The experiments were

repeated three times independently with similar results. Source data are provided as a Source Data file.

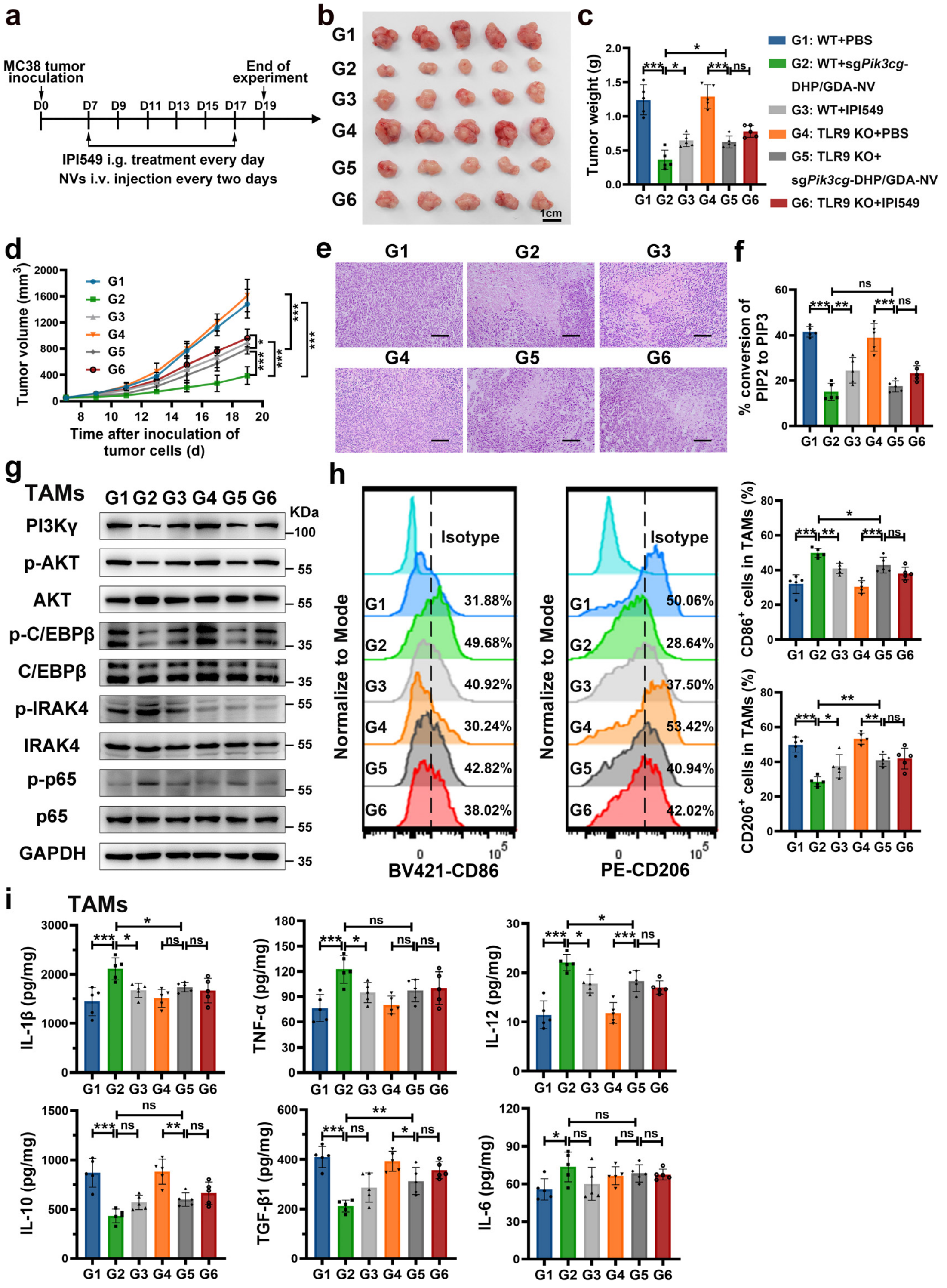

Supplementary Fig.27 TLR9 gene deletion impiared the therapeutic effects of sg*Pik3cg*-DHP/DGA-NVs in MC38 tumor-bearing mice. **a** Schematic diagram of MC38 tumor-bearing wild type (WT) mice and TLR gene knockout (TLR9 KO) mice with an in-vein injection of sg*Pik3cg*-DHP/DGA-NVs (1×10^{10} NVs every two days) or intragastric administration of IPI549 (15 mg/kg every day). **b-c** Images of tumors and mean tumor weights from mice with different NVs or IPI549 treatment at day 19 post to the inoculation of tumor cells. n = 5 mice per group. **d** Tumor volume curves in the different treatment groups of MC38 tumor-bearing mice. n = 5 mice per group. **e**

Representative images of H&E staining of MC38 tumor sections. Scale bar, 100 μm. n = 5 mice per group. **f** Phosphoinositide 3-kinase activity of TAMs, characterized by the conversion ratio of PIP2 to PIP3, was determined by ELISA on day 19 post to model establishment. n = 5 mice per group. **g** The levels of PI3Kγ and TLR9 pathways related molecules (p-C/EBPβ, p-AKT, p-IRAK4 and p-p65) in TAMs isolated from mice with above mentioned treatments were detected by western blotting at 19 days post to the inoculation of tumor cells. The experiments were repeated three times independently with similar results. **h-i** The effects of sg*Pik3cg*-DHP/DGA-NVs on TAMs phenotype and derived cytokines were identified by flow cytometry analysis (CD86 and CD206) and ELISA. n = 5 mice per group. Data are represented as mean ± SD. Two-way ANOVA with Dunnett's multiple comparison test was used in panel **d**. Other statistical analyses were performed using one-way ANOVA with Dunnett's multiple comparison test. *$P < 0.05$, **$P < 0.01$ and ***$P < 0.001$. ns, no significant change. The exact *P*-value and source data are provided as a Source Data file.

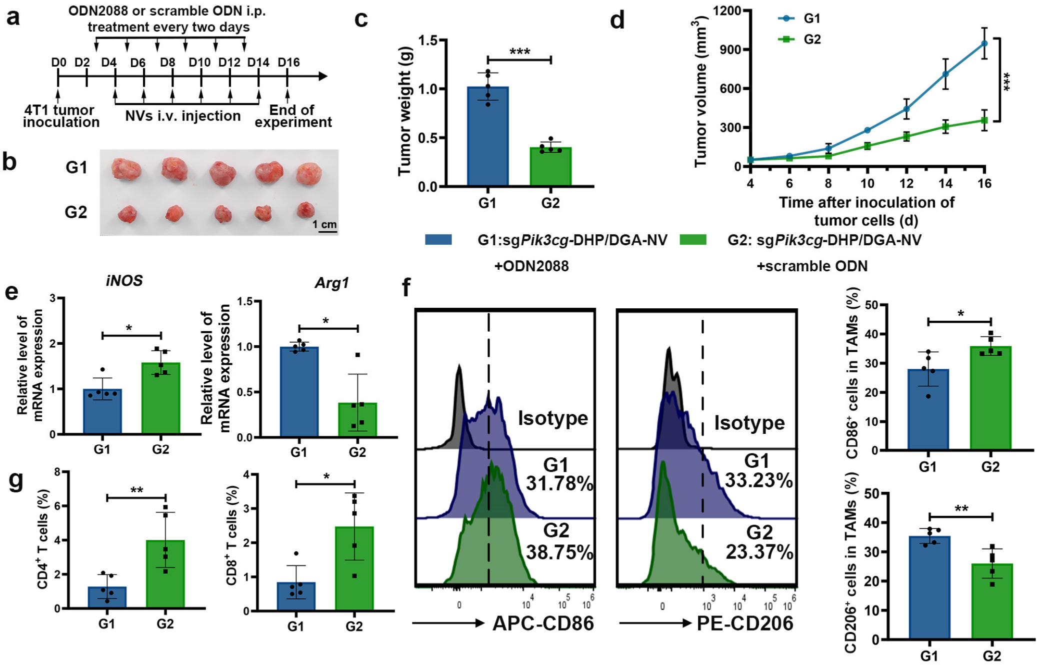

Supplementary Fig.28 TLR9 antagonist OND2088 partially abolished the therapeutic effect of sg*Pik3cg*-DHP/DGA-NVs in 4T1 tumor-bearing mice. a Schematic diagram of 4T1 tumor-bearing mice with an in-vein injection of sg*Pik3cg*-DHP/DGA-NVs (1×10^{10} NVs per mice every two days) and intraperitoneal injection of TLR9 antagonist ODN2088 or scramble ODN (50 μg per mice every two days). **b-c** Images of tumors and mean tumor weights from mice with different NVs and ODN2088 or scramble ODN treatment at day 16 post to the inoculation of tumor cells. **d** Tumor volume curves in the different treatment groups of 4T1 tumor-bearing mice. **e-f** The effects of sg*Pik3cg*-DHP/DGA-NVs on TAMs phenotype were identified by qRT-PCR assay (*iNOS* and *Arg1*) and flow cytometry analysis (CD86 and CD206). **g** The influence of sg*Pik3cg*-DHP/DGA-NVs on intratumor $CD4^+$/$CD8^+$ T cell ratio were identified by flow cytometry analysis. n = 5 mice per group for panels (**b-g**). Data are represented as mean ± SD. Two-way ANOVA with Bonferroni's multiple comparison test was used in panel **d.** Other statistical analyses were performed using unpaired Student's t test (two tailed) except RT-qPCR assay on the relative level of *Arg1* expression (unpaired t test with Welch's correction (two tailed)), *iNOS* expression in **e** and FACS analysis on $CD8^+$ T cell in panel **g** (Mann Whitney test). *$P < 0.05$, **$P < 0.01$ and ***$P < 0.001$. The exact P-value and source data are provided as a Source Data file.

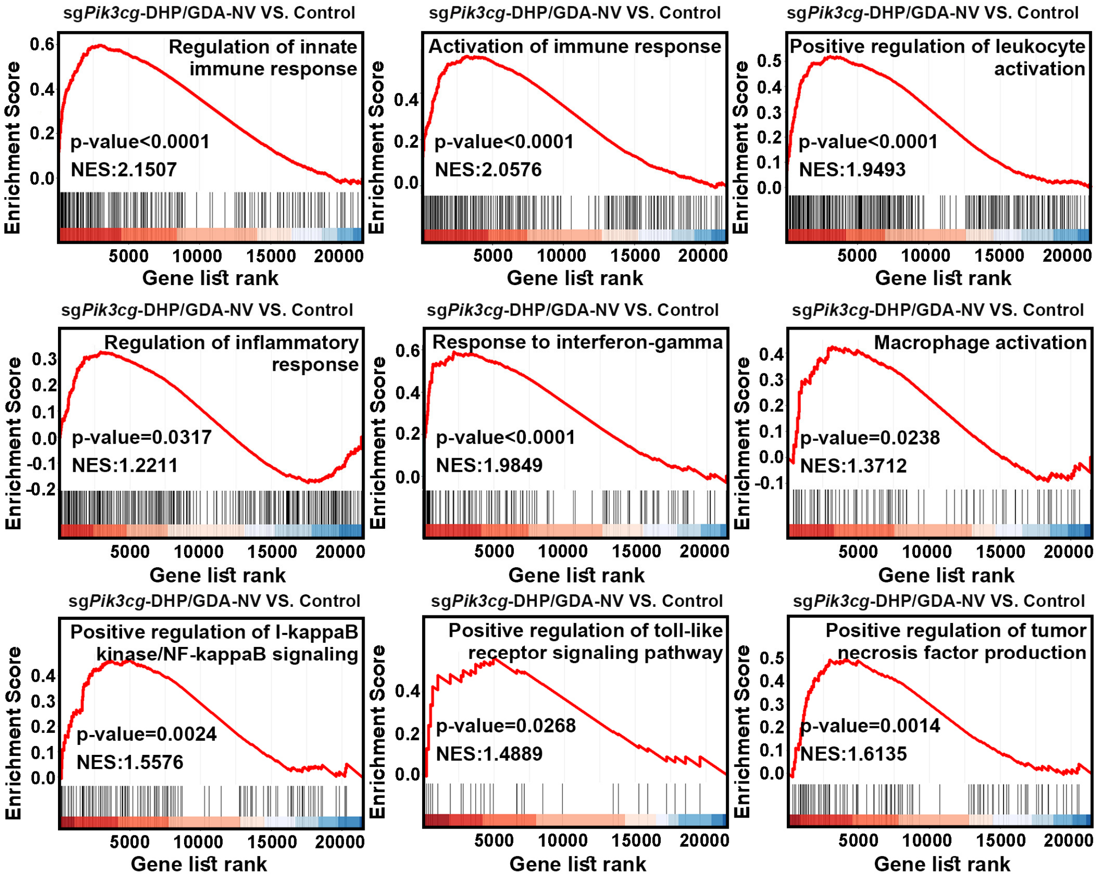

Supplementary Fig. 29 Gene Set Enrichment Analysis (GSEA) comparing the expression of genes involved in immune activation in 4T1 tumor tissue. The comparison is between the sg*Pik3cg*-DHP/DGA-NVs treated group, receiving 1×10^{10} NVs every two days, and the control group, assessed on day 16 after the establishment of the model. n = 3 mice per group.

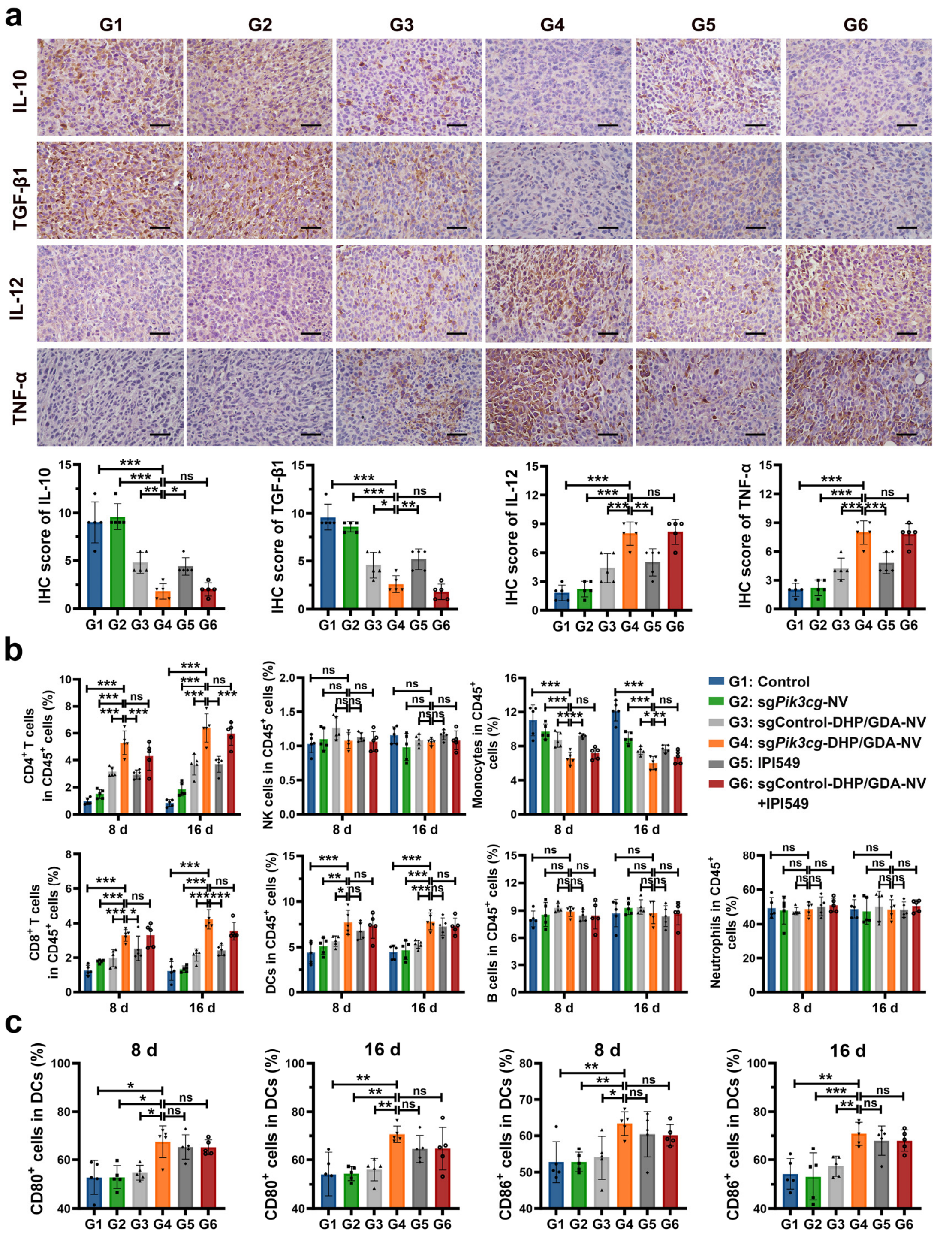

Supplementary Fig. 30 sg*Pik3cg*-DHP/DGA-NVs therapy improved the tumor microenvionment of 4T1 tumor-bearing mice. a Resprentative immunohistochemical staining of immunosuppressive cytokines (IL-10 and TGF-β1) and immunostimulatory cytokines (IL-12 and TNF-α) in 4T1 tumor tissue from mice with an in-vein injection of NVs (1×10^{10} NVs every two days) or intragastric administration of IPI549 (15 mg/kg every day) at 16 days post to 4T1 model establishment. **b-c** The influence of sg*Pik3cg*-DHP/DGA-NVs on the ratio of

intratumoral leukocyte and activated DC ($CD80^+$ and $CD86^+$) was evaluated by flow cytometry analysis at 8 days and 16 days post to the inoculation of tumor cells. n = 5 mice per group for panels (**a**-**c**). Data are represented as mean ± SD. Statistical analyses were performed using one-way ANOVA with Dunnett's multiple comparison test except FACS analysis on NK cell on 16 d, FACS analysis on neutrophil on 8 and 16 d and FACS analysis on $CD80^+$ cell in DC on 8 d (Kruskal-Wallis test with Dunn's multiple comparisons test). *$P < 0.05$, **$P < 0.01$ and ***$P < 0.001$. The exact *P*-value and source data are provided as a Source Data file.

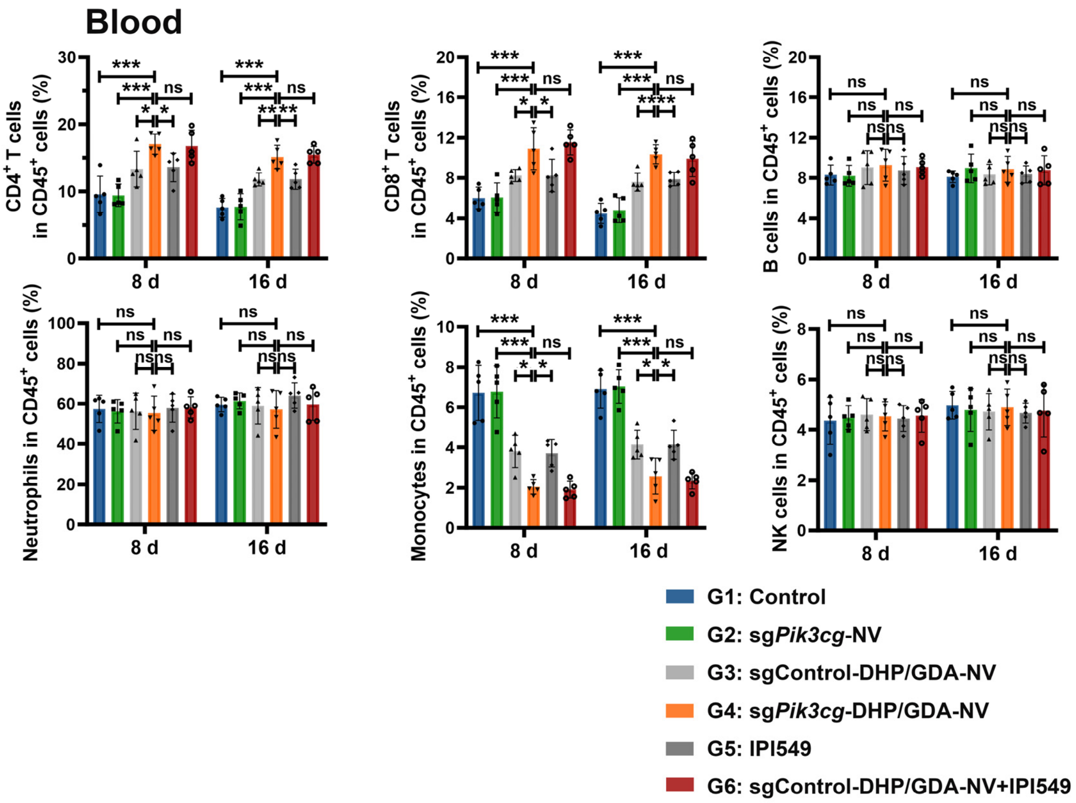

Supplementary Fig. 31 The influence of sg*Pik3cg*-DHP/DGA-NVs and IPI549 treatment on the immune cell ratio of peripheral blood leukocyte from 4T1 tumor bearing mice treated with sg*Pik3cg*-DHP/DGA-NVs or IPI549. 4T1 tumor-bearing mice were treated with intravenous injections of NVs at a dose of 1×10^{10} NVs every two days, or through daily intragastric administration of IPI549 at a dosage of 15 mg/kg. The ratio of immune cells in the peripheral blood leukocyte was evaluated using flow cytometry analysis at 16 days after the inoculation of tumor cells. n = 5 mice for each time point. Data are represented as mean ± SD. Statistical analyses were performed using one-way ANOVA with Dunnett's multiple comparison test. $^{*}P < 0.05$, $^{**}P < 0.01$ and $^{***}P < 0.001$. ns, no significant change. The exact *P*-value and source data are provided as a Source Data file.

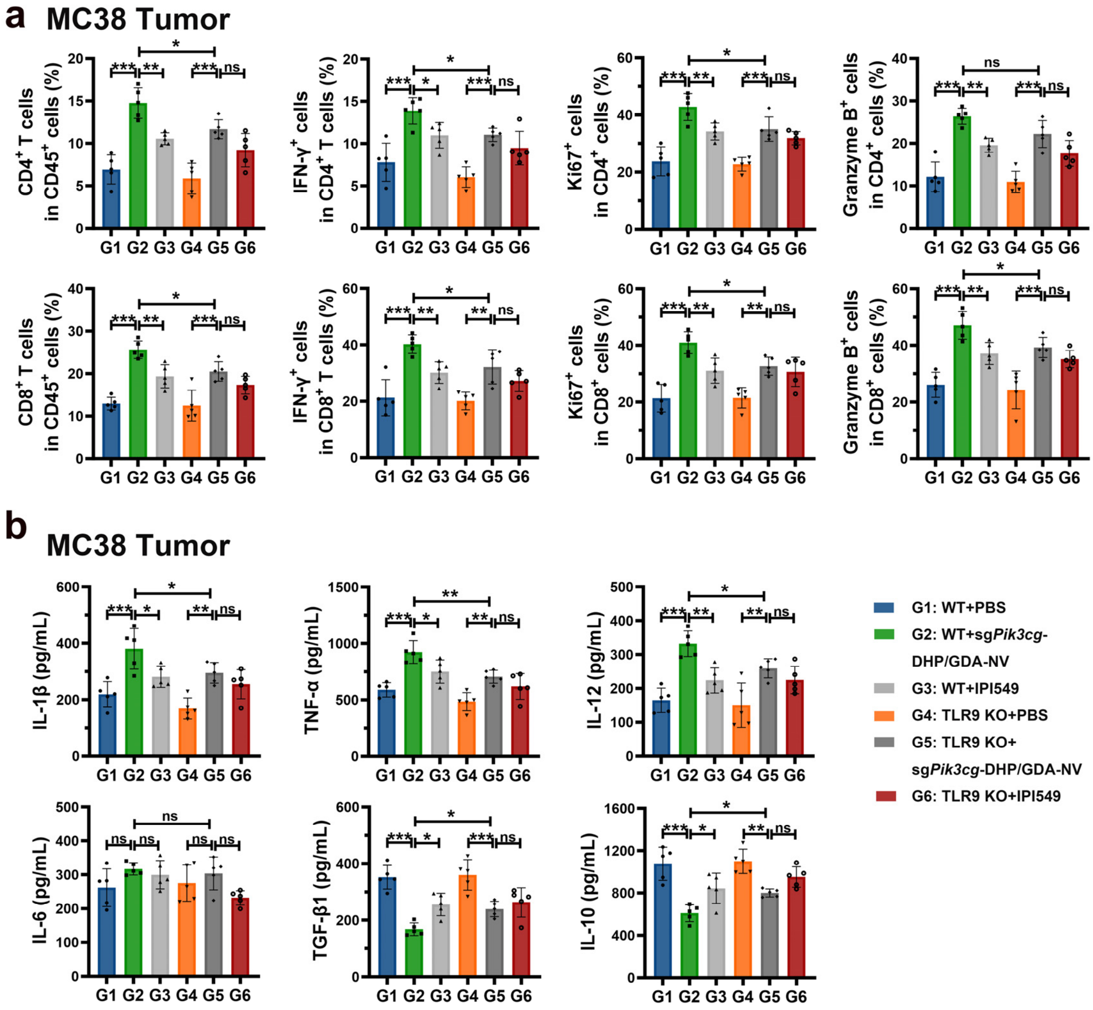

Supplementary Fig. 32 sg*Pik3cg*-DHP/DGA-NVs therapy improved the tumor microenvionment of MC38 tumor-bearing mice. a The influence of sg*Pik3cg*-DHP/DGA-NVs on intratumoral T cell activation and proliferation (IFN-γ^+, ki67$^+$ and Granzyme B$^+$ percentage in CD4$^+$ T cells and CD8$^+$ T cells) was evaluated in MC38 WT and TLR9 KO mice with an in-vein injection of sg*Pik3cg*-DHP/DGA-NVs (1×10^{10} NVs every two days) or intragastric administration of IPI549 (15 mg/kg every day) at day 19 post to the inoculation of tumor cells. **b** The cytokine levels in MC38 tumors harvested from mice with above mentioned treatments were determined by ELISA assay. n = 5 mice per group for panels (**a**-**b**). Data are represented as mean ± SD. Statistical analyses were performed using one-way ANOVA with Dunnett's multiple comparison test. *$P < 0.05$, **$P < 0.01$ and ***$P < 0.001$. ns, no significant change. The exact *P*-value and source data are provided as a Source Data file.

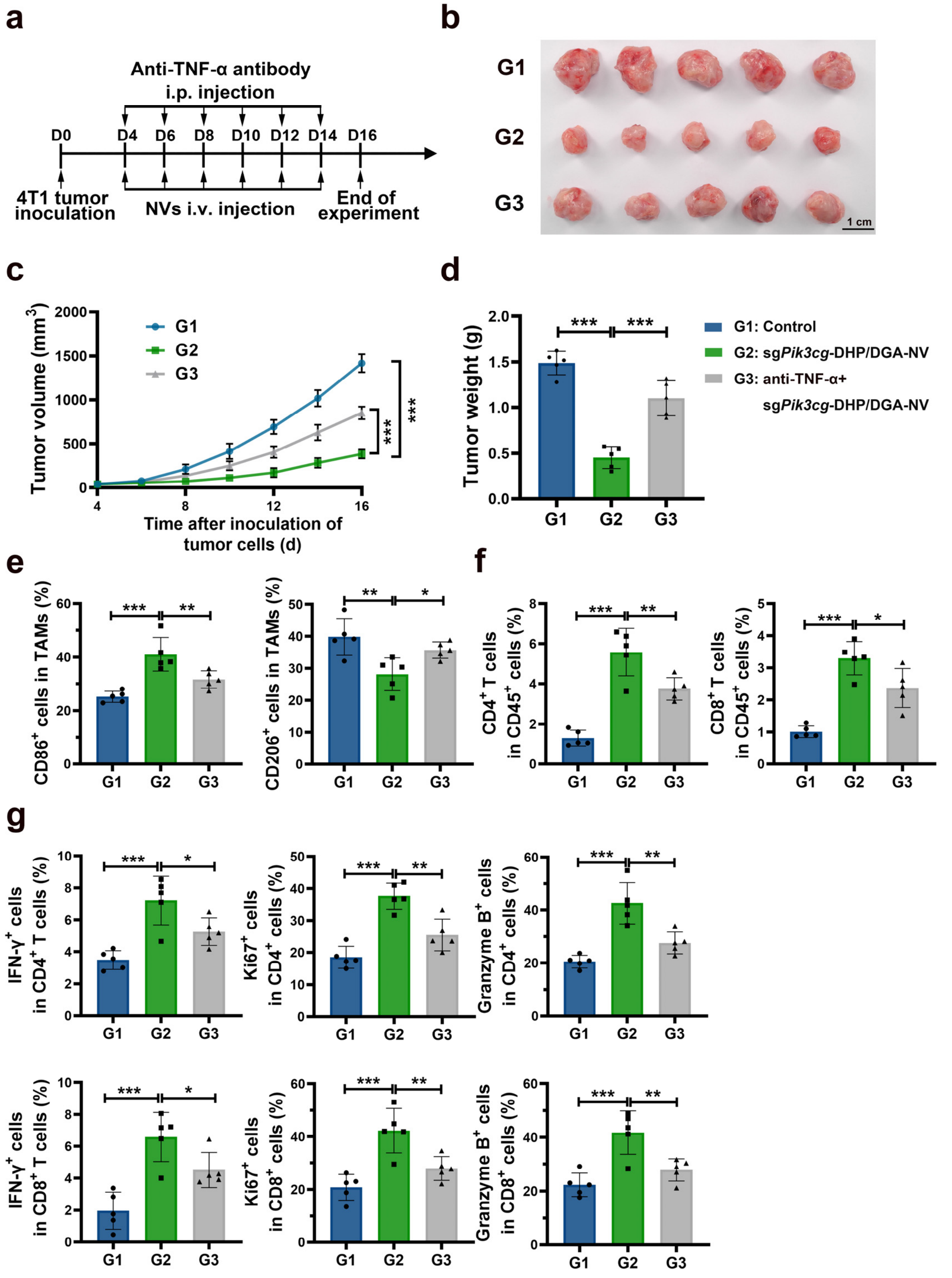

Supplementary Fig. 33 The influence of TNF-α blockade on sg*Pik3cg*-DHP/DGA-NVs tumor immunotherapy in 4T1 tumor-bearing mice. a Schematic diagram of 4T1 tumor-bearing mice with an in-vein injection of NVs (1×10^{10} NVs every two days) and intraperitoneal injection of anti-TNF-α antibody (500 μg per mouse every 2 days). **b-c** Images of tumors and tumor volume curves of mice with above mentioned treatment at day 16 post to the inoculation of tumor cells. **d** Mean tumor weights in the different treatment groups of 4T1 tumor-bearing mice. **e-g** The effects of sg*Pik3cg*-

DHP/DGA-NVs on TAMs phenotype, intratumor $CD4^+$/$CD8^+$ T cell ratio and T cell activation and proliferation (IFN-γ^+, $Ki67^+$ and Granzyme B^+ percentage) were identified by flow cytometry analysis. n = 5 mice per group for panels (**b-g**). Data are represented as mean ± SD. Two-way ANOVA with Dunnett's multiple comparison test was used in panel **c.** Other statistical analyses were performed using one-way ANOVA with Dunnett's multiple comparison test. **P* < 0.05, ***P* < 0.01 and ****P* < 0.001. ns, no significant change. The exact *P*-value and source data are provided as a Source Data file.

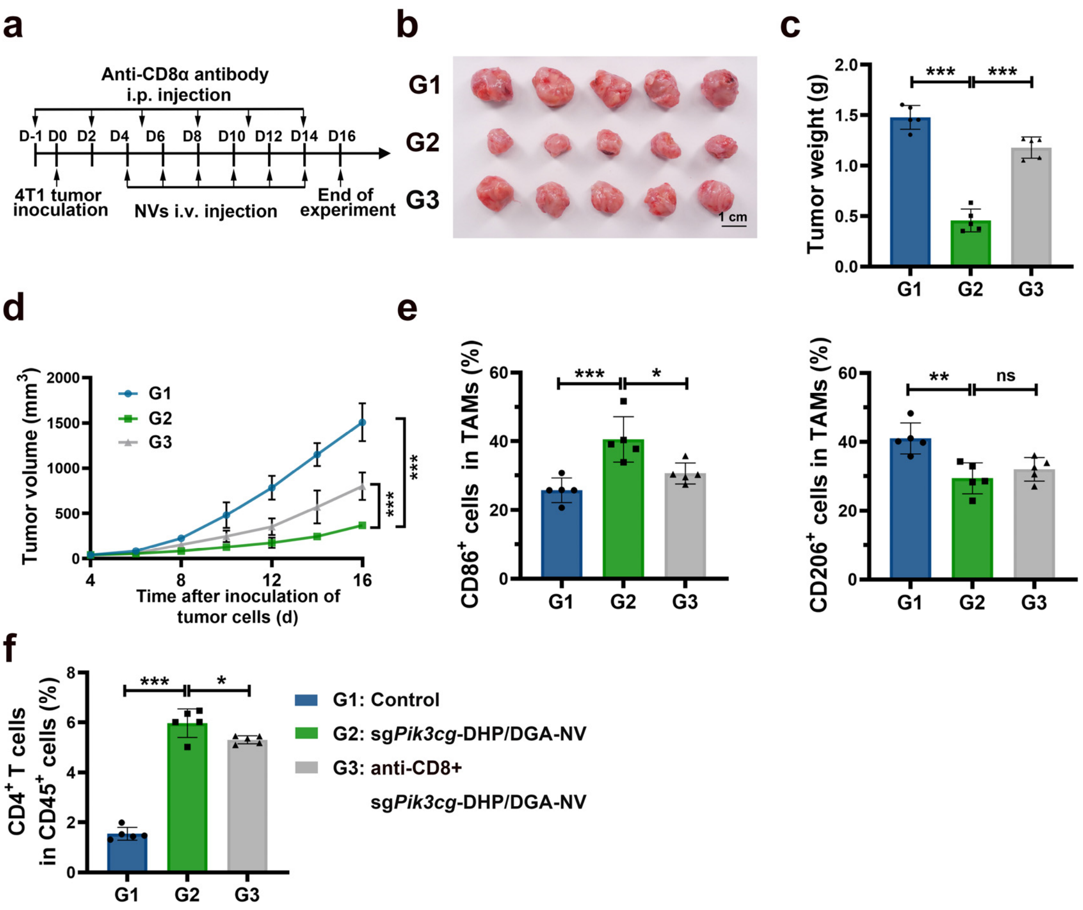

Supplementary Fig. 34 The influence of CD8$^+$ T cells deletion on sg*Pik3cg*-DHP/DGA-NVs tumor immunotherapy in 4T1 tumor-bearing mice. a Schematic diagram of 4T1 tumor-bearing mice with an in-vein injection of NVs (1×10^{10} NVs every two days) and intraperitoneal injection of anti-CD8α antibody (100 μg per mouse every 3 days). **b-c** Images of tumors and mean tumor weights from mice with above mentioned treatment at day 16 post to the inoculation of tumor cells. **d** Tumor volume curves in the different treatment groups of 4T1 tumor-bearing mice. **e-f** The effects of sg*Pik3cg*-DHP/DGA-NVs on TAMs phenotype and intratumor CD4$^+$ T cell ratio were identified by flow cytometry analysis. n = 5 mice per group for panels (**b**-**f**). Data are represented as mean ± SD. Two-way ANOVA with Dunnett's multiple comparison test was used in panel **d.** Other statistical analyses were performed using one-way ANOVA with Dunnett's multiple comparison test. *$P < 0.05$, **$P < 0.01$ and ***$P < 0.001$. The exact *P*-value and source data are provided as a Source Data file.

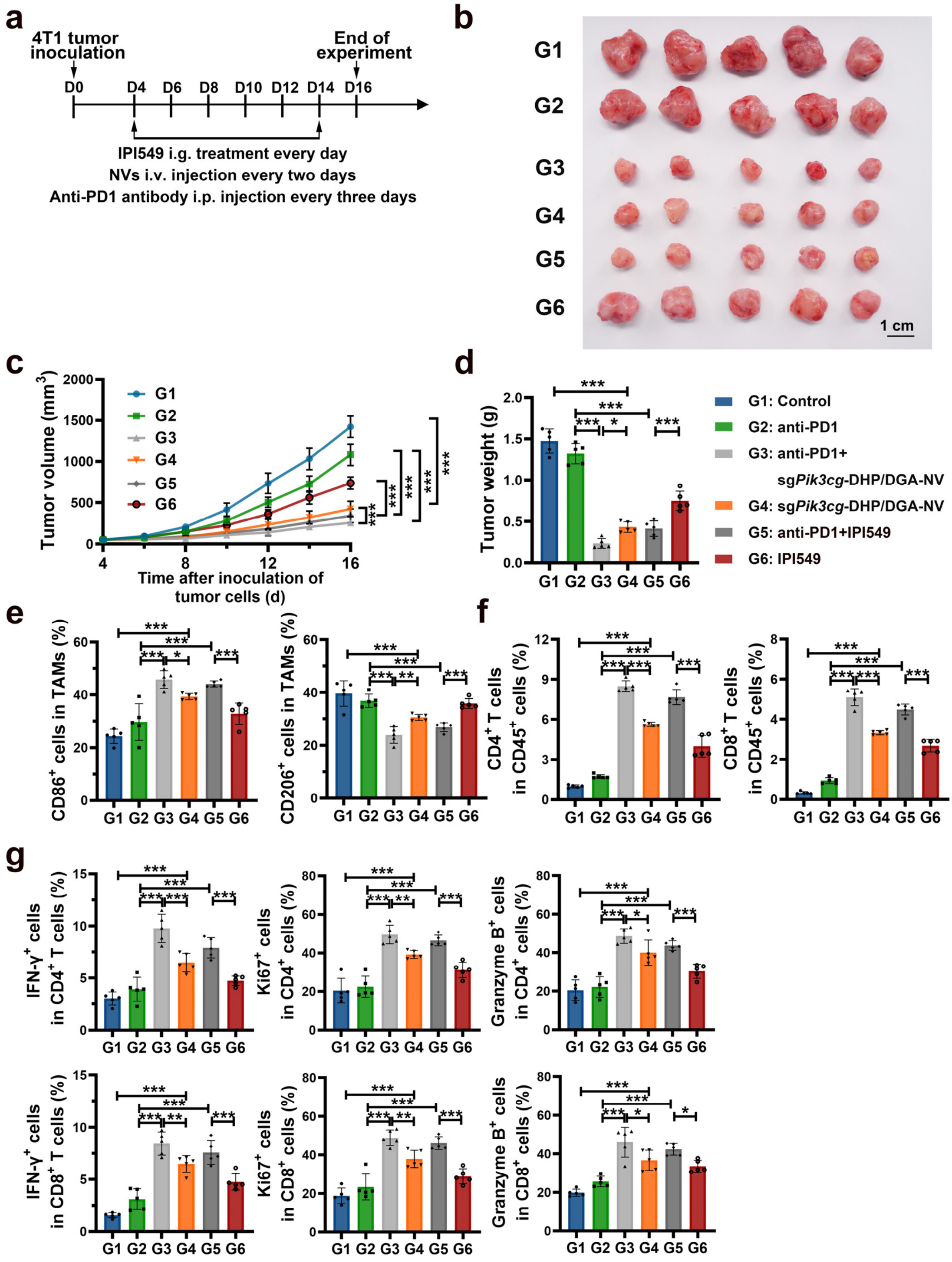

Supplementary Fig. 35 The therapeutic effect of the combined treatment of anti-PD-1 antibody and sg*Pik3cg*-DHP/DGA-NVs in 4T1 tumor-bearing mice. a Schematic diagram of 4T1 tumor-bearing mice with an in-vein injection of NVs (1×10^{10} NVs every two days), intragastric administration of IPI549 (15 mg/kg every day) and intraperitoneal injection of anti-PD-1 antibody (250 μg per mouse every 3 days). **b-c** Representative images of tumors and tumor volume curves of mice with above mentioned treatment at day 16 post to the inoculation of tumor cells. **d** Mean tumor

weights in the different treatment groups of 4T1 tumor-bearing mice. **e-g** The effects of sg*Pik3cg*-DHP/DGA-NVs on TAMs phenotype and intratumor $CD4^+/CD8^+$ T cell ratio and T cell activation and proliferation (IFN-γ^+, $Ki67^+$ and Granzyme B^+ percentage) were identified by flow cytometry analysis. n = 5 mice per group for panels (**b-g**). Data are represented as mean ± SD. Two-way ANOVA with Dunnett's multiple comparison test was used in panel **c.** Other statistical analyses were performed using one-way ANOVA with Dunnett's multiple comparison test. $*P < 0.05$, $**P < 0.01$ and $***P < 0.001$. The exact *P*-value and source data are provided as a Source Data file.

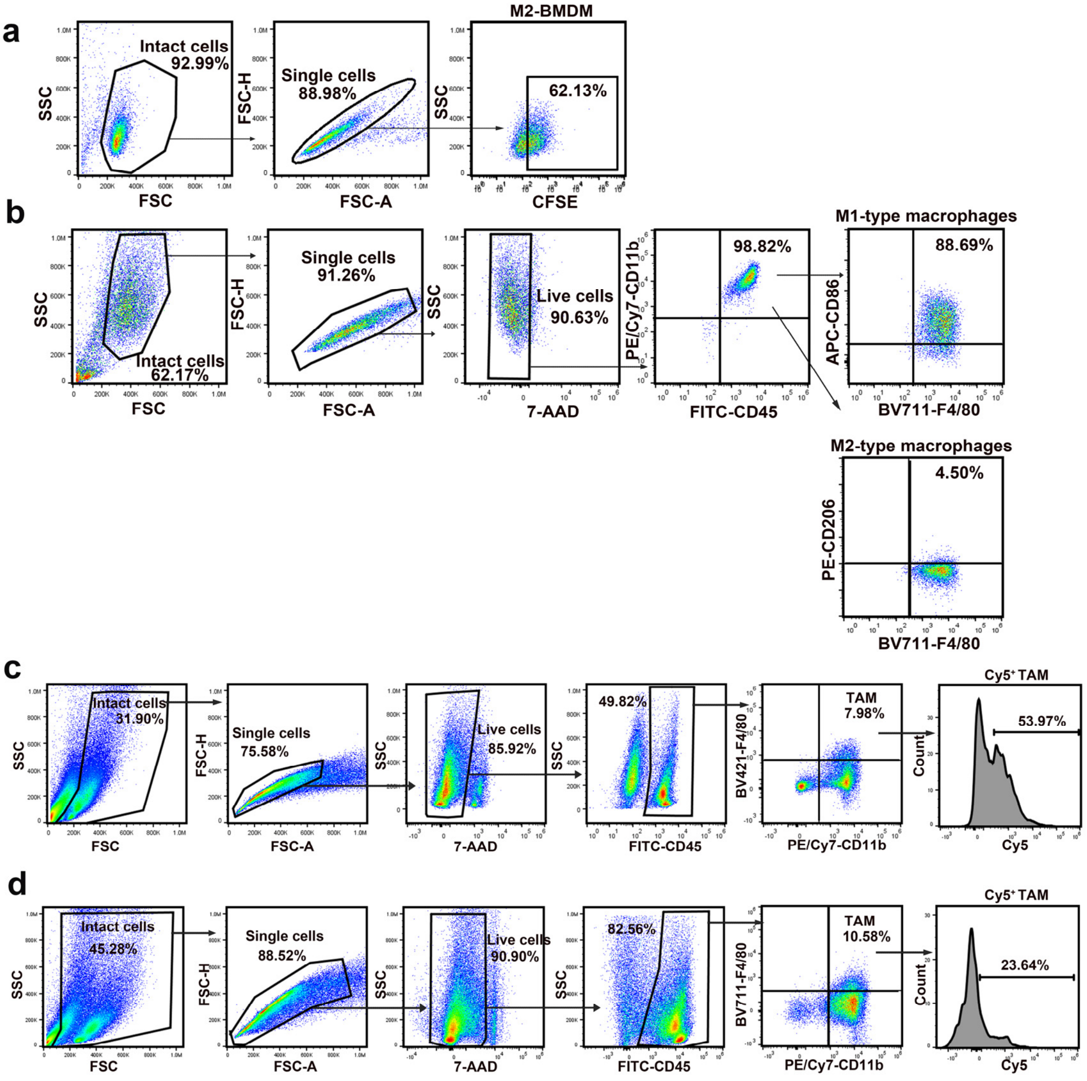

Supplementary Fig. 36 Gating strategies of macrophages used for flow cytometry analysis. **a** Gating strategies of CFSE$^+$ M2-BMDM in Fig. 4a-b,e-f, Supplementary Fig. 21b, Supplementary Fig. 17d. **b** Gating strategies of CD86$^+$ M1-macrophages and CD206$^+$ M2-macrophages in Fig. 5h. **c** Gating strategies of Cy5$^+$ TAM in Fig. 6g. **d** Gating strategies of Cy5$^+$ TAM in Fig. 6i and Supplementary Fig. 24e.

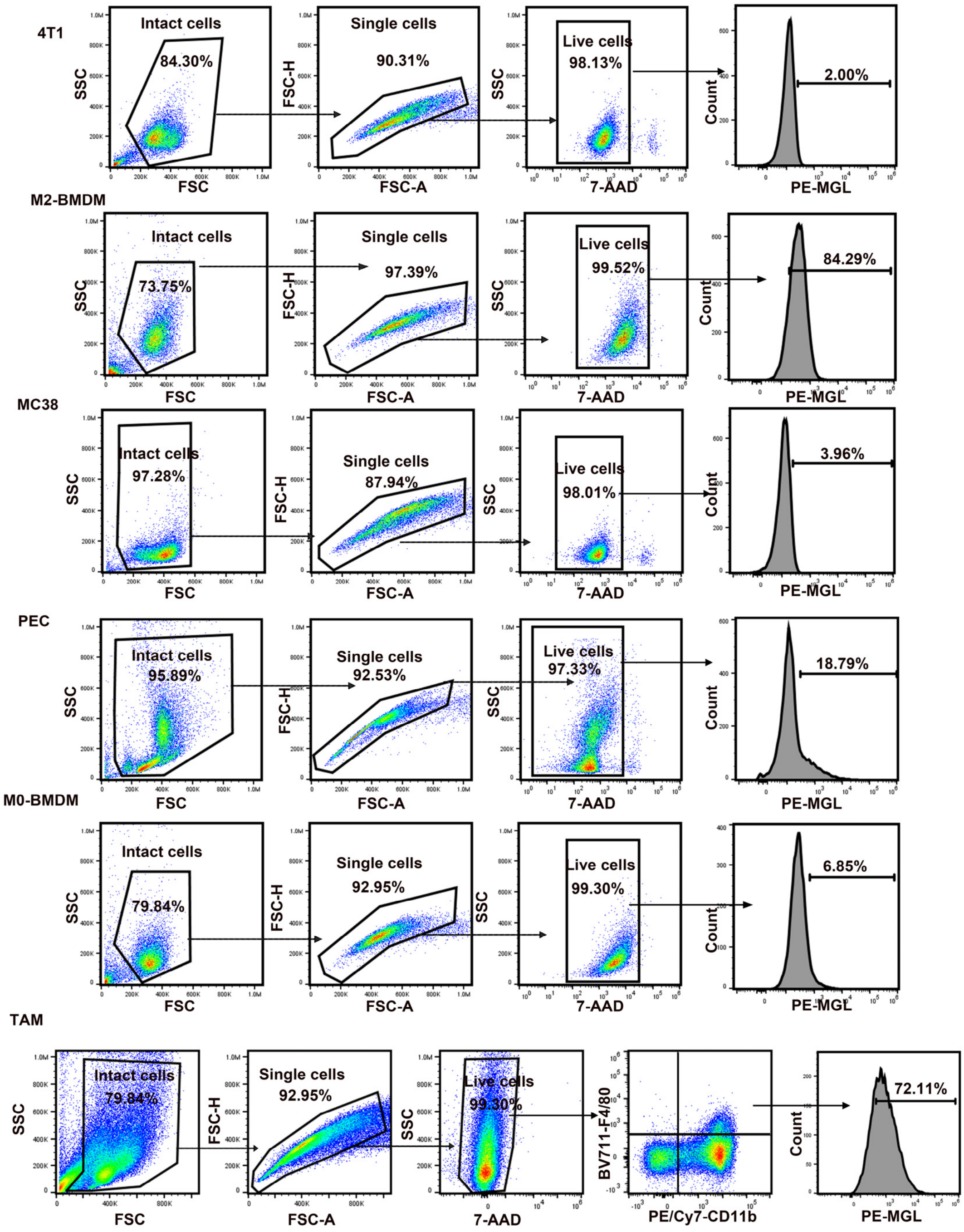

Supplementary Fig. 37 Gating strategies of determing MGL expression in cells used for flow cytometry analysis. This gating strategy corresponded to the FACS data in Supplementary Fig. 16b and Supplementary Fig. 17b.

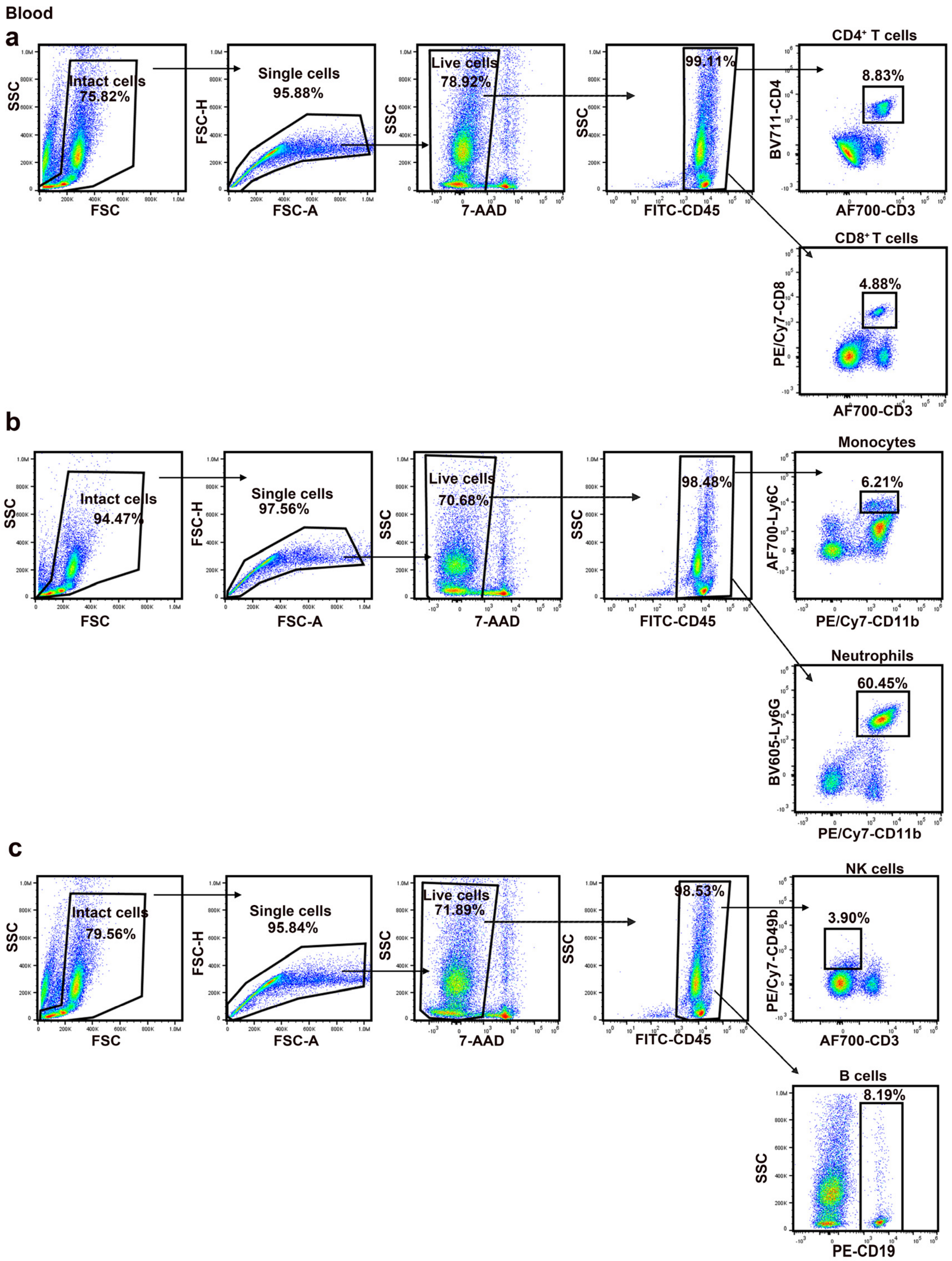

Supplementary Fig. 38 Gating strategies of peripheral blood cells used for flow cytometry analysis. a $CD4^+$ T lymphocyte and $CD8^+$ T lymphocytes of $CD45^+$ leukocytes; **b** Monocytes ($CD11b^+Ly6C^+$) and neutrophils ($CD11b^+Ly6G^+$) from $CD45^+$ leukocytes; **c** B cells ($CD19^+$) and NK cells ($CD3^-CD49b^+$) of $CD45^+$ leukocyte cells in the blood. This gating strategy corresponded to the FACS data in Supplementary Fig. 22b and Supplementary Fig. 31.

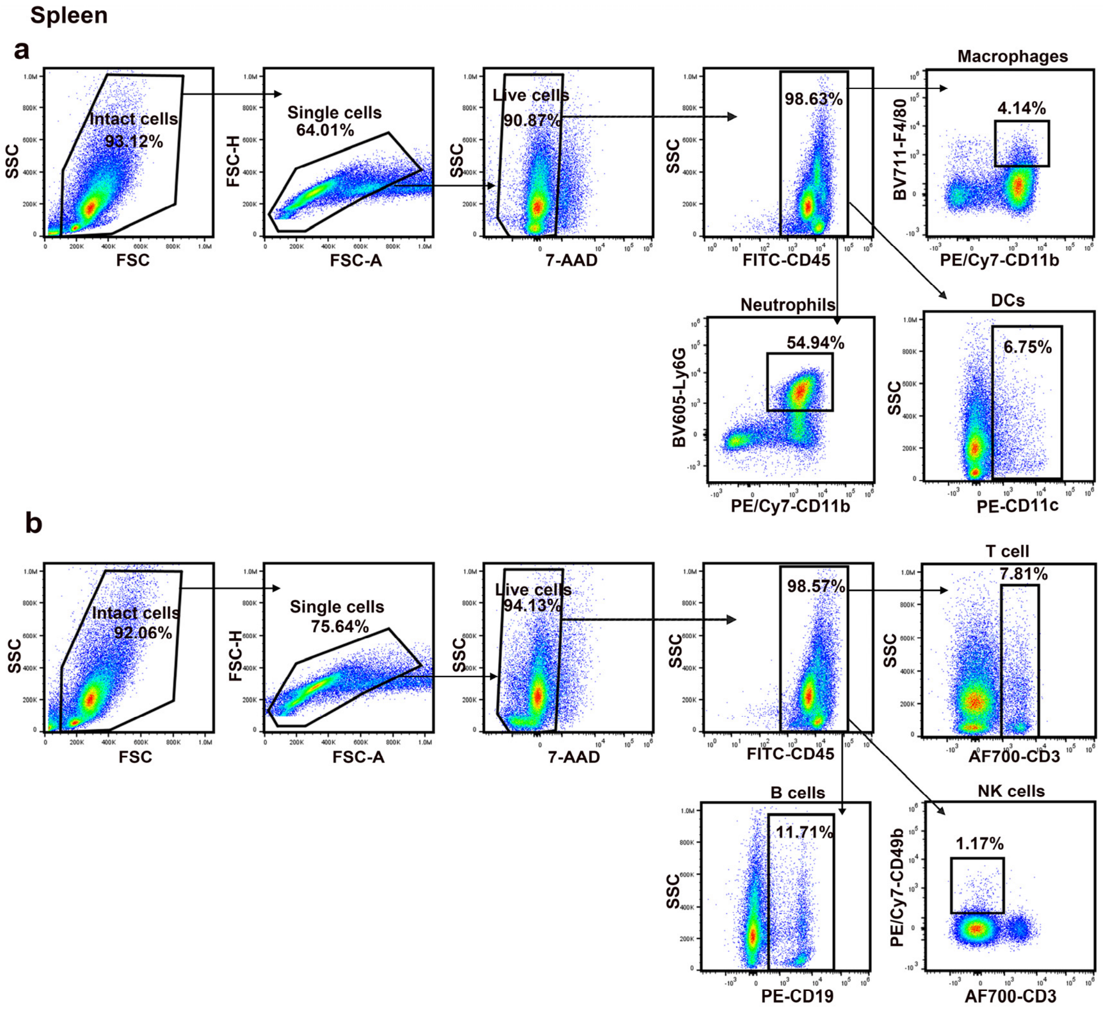

Supplementary Fig. 39 Gating strategies of splenocytes used for flow cytometry analysis. a Neutrophils ($CD11b^+Ly6G^+$), DCs ($CD11c^+$) and macrophages ($CD11b^+F4/80^+$) of $CD45^+$ leukocytes; **b** T lymphocytes ($CD3^+$), B cells ($CD19^+$) and NK cells ($CD3^-CD49b^+$) of $CD45^+$ leukocyte cells in the spleen. This gating strategy corresponded to the FACS data in Supplementary Fig. 10f and Supplementary Fig. 22b.

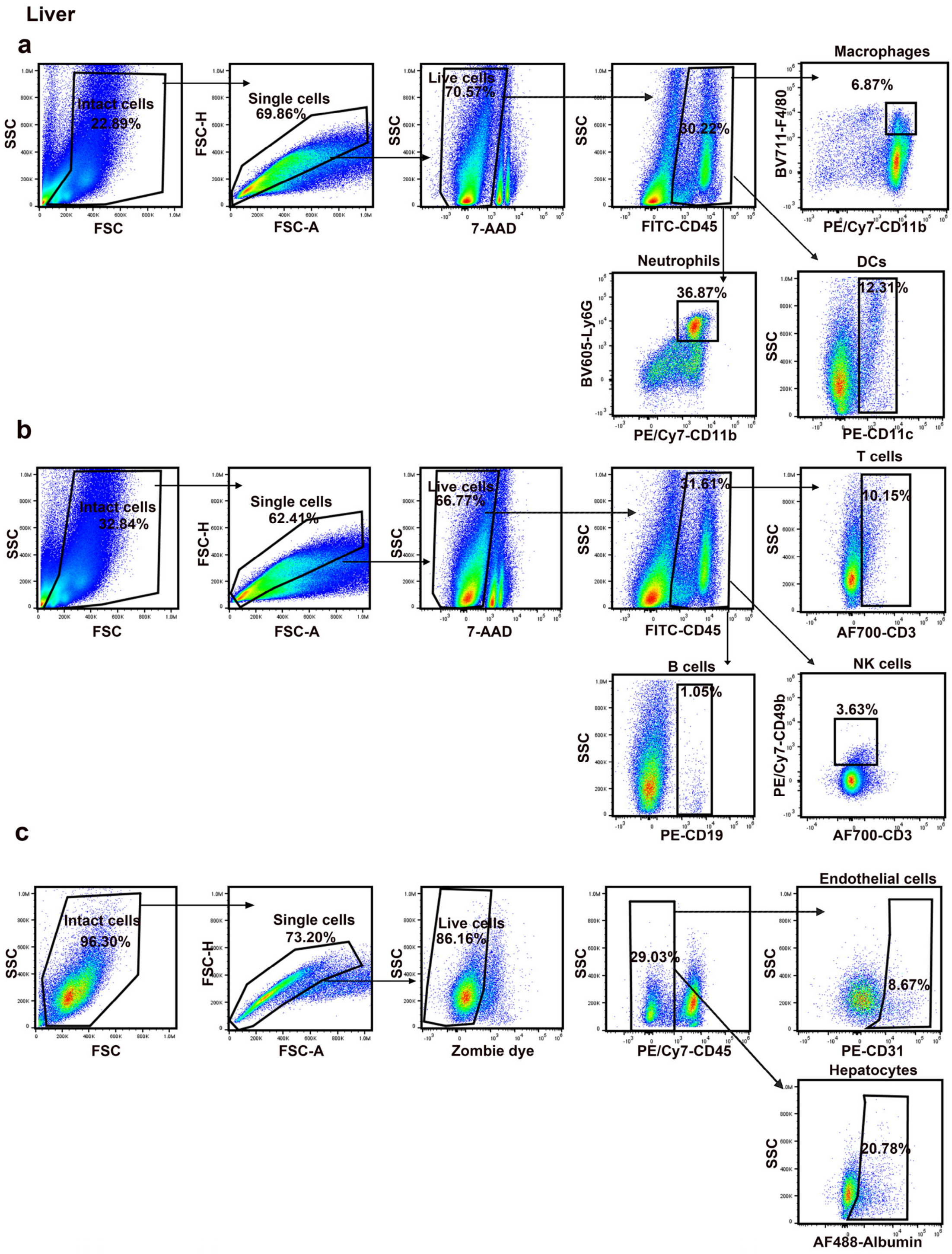

Supplementary Fig. 40 Gating strategies of hepatic cell population used for flow cytometry analysis. a Neutrophils (CD11b$^+$Ly6G$^+$), DCs (CD11c$^+$) and macrophages (CD11b$^+$F4/80$^+$) of CD45$^+$ leukocytes; **b** T lymphocytes (CD3$^+$), B cells (CD19$^+$) and NK cells (CD3$^-$CD49b$^+$) of CD45$^+$ leukocyte cells in the liver. **c** Hepatocytes (CD45$^-$ Albumin$^+$) and endothelial cell (CD45$^-$ CD31$^+$). This gating strategy corresponded to the FACS data in Supplementary Fig. 10g and Supplementary Fig. 22b.

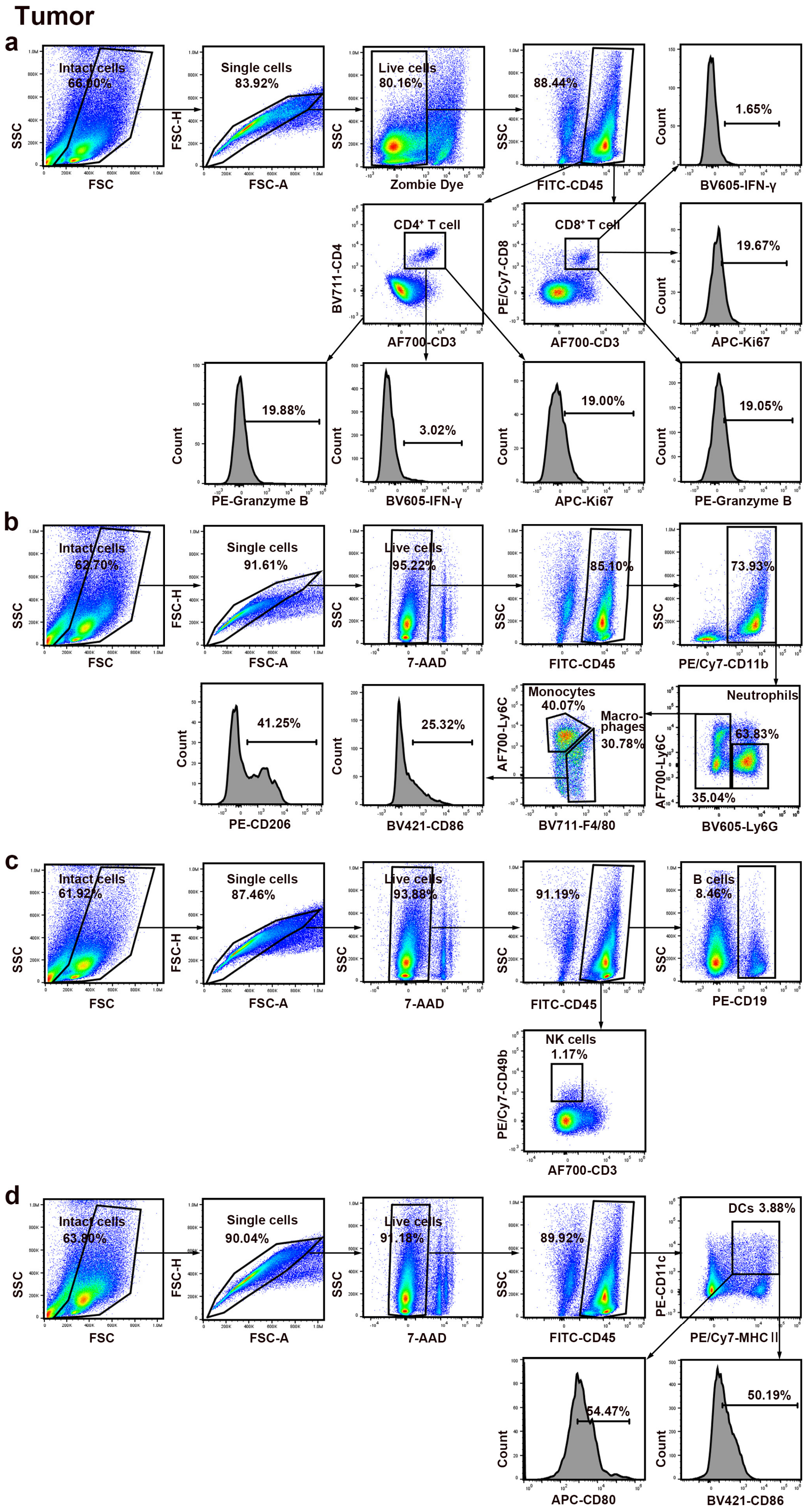
Tumor
a
Intact cells 66.90%
Single cells 83.92%
Live cells 80.16%
88.44%
1.65%
CD4+ T cell
CD8+ T cell
19.67%
19.88%
3.02%
19.00%
19.05%
SSC
FSC
FSC-H
FSC-A
Zombie Dye
FITC-CD45
Count
BV605-IFN-γ
BV711-CD4
AF700-CD3
PE/Cy7-CD8
APC-Ki67
PE-Granzyme B
b
Intact cells 62.70%
Single cells 91.61%
Live cells 95.22%
85.10%
73.93%
7-AAD
PE/Cy7-CD11b
41.25%
25.32%
Monocytes 40.07%
Macro-phages 30.78%
Neutrophils
63.83%
35.04%
PE-CD206
BV421-CD86
AF700-Ly6C
BV711-F4/80
BV605-Ly6G
c
Intact cells 61.92%
Single cells 87.46%
Live cells 93.88%
91.19%
B cells 8.46%
PE-CD19
NK cells 1.17%
PE/Cy7-CD49b
d
Intact cells 63.80%
Single cells 90.04%
Live cells 91.18%
89.92%
DCs 3.88%
PE-CD11c
PE/Cy7-MHC II
54.47%
50.19%
APC-CD80

e

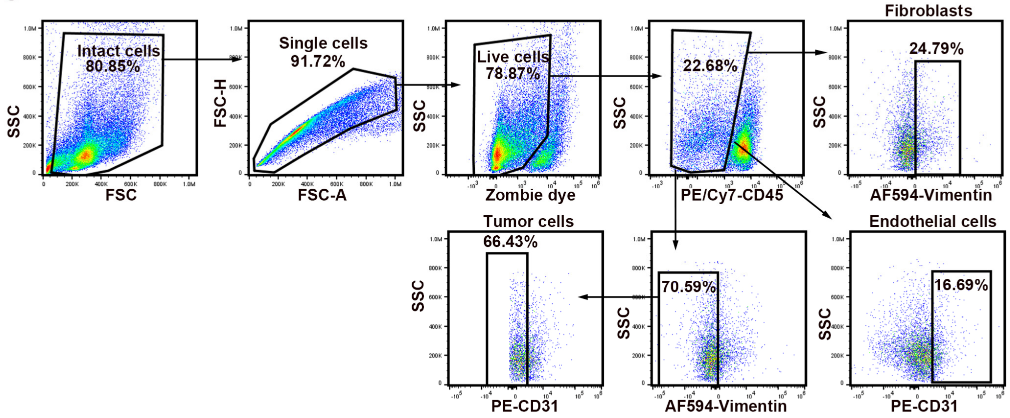

Supplementary Fig. 41 Gating strategies of cell population in tumor used for flow cytometry analysis. a $CD4^+$ and $CD8^+$ T lymphocyte from $CD45^+$ leukocytes in the tumor used for the *in vivo* antitumor assessment. $Ki67^+$cells,Granzyme B^+ cells and IFN-γ^+ cells in $CD4^+$ and $CD8^+$ T lymphocytes in the tumor used in the *in vivo* T lymphocytes activation assessment. **b** Monocyte ($CD11b^+Ly6G^-Ly6C^+$), and neutrophil ($CD11b^+$ $Ly6C^-Ly6G^+$) of $CD45^+$ leukocytes. $CD86^+$ cells and $CD206^+$ cells in $CD11b^+Ly6C^-F4/80^+$ macrophages in the tumor. **c** B cells ($CD19^+$) and NK cells ($CD3^-$ $CD49b^+$) from $CD45^+$ leukocytes in the tumor. **d** DCs ($MHCII^+CD11c^+$) of $CD45^+$ leukocytes in the tumor. $CD80^+$ cells and $CD86^+$ cells in DCs in the tumor. **e** Fibroblasts ($CD45^-Vimentin^+$), endothelial cells ($CD45^-CD31^+$) and tumor cells ($CD45^-Vimentin^-$ $CD31^-$) in tumor tissue. This gating strategy corresponded to the FACS data in Fig. 7h, Fig. 8e and Supplementary Fig. 22b, Supplementary Fig. 26a, Supplementary Fig. 27h, Supplementary Fig. 28f-g, Supplementary Fig. 30b-c, Supplementary Fig. 32a-b, Supplementary Fig. 33e-g, Supplementary Fig. 34e-f, Supplementary Fig. 35e-g.

Supplementary Tables

Supplementary Table 1 Characterization of free NVs and sg*Pik3cg*-DHP/DGA-NVs measuring by Malvern instrument. Data are presented as the means ± SD. n = 3 biologically independent samples.

	Mean value of particle size (nm)	Mode value of particle size (nm)	Zeta (mV)
Free NVs	148.8±5.34	124.2±4.40	-47.1667±0.32
sg*Pik3cg*-DHP/DGA-NVs	149.1±3.49	126.5±8.75	-45.7667±1.33

Supplementary Table 2 Characterization of free NVs and sg*Pik3cg*-DHP/DGA-NVs measuring by NanoBrook 90Plus. Data are presented as the means ± SD. n = 4 biologically independent samples.

	Mean value of particle size (nm)	PDI
Free NVs	152.20±2.93	0.243±0.020
sg*Pik3cg*-DHP/DGA-NVs	157.92±4.29	0.228±0.009

Supplementary Table 3 The levels of genome-editing system in sg*Pik3cg-E. coli*, sg*Pik3cg*-DHP/DGA-NV and OMV. Data are presented as the means ± SD. n = 3 biologically independent samples.

	Cas9 (molecules/cell or NV)	sgRNA (copies/cell or NV)
sg*Pik3cg*-*E. coli*	$(3.40\pm0.64)\times10^5$	$(3.05\pm0.26)\times10^6$
sg*Pik3cg*-DHP/DGA-NV	$(6.26\pm0.20)\times10^3$	$(5.97\pm 1.17)\times10^4$
OMV	$(5.94\pm0.83)\times10^2$	$(4.27\pm 0.64)\times10^3$

Supplementary References

1. Zhao C., *et al.* "Sheddable" PEG-lipid to balance the contradiction of PEGylation between long circulation and poor uptake. *Nanoscale* **8**, 10832-10842 (2016).
2. Pham V. H., Phan T. P. D., Phan D. C.&Vu B. D. Synthesis and Bioactivity of Hydrazide-Hydrazones with the 1-Adamantyl-Carbonyl Moiety. *Molecules* **24**, (2019).
3. Kim H.-K., *et al.* Effective targeted gene delivery to dendritic cells via synergetic interaction of mannosylated lipid with DOPE and BCAT. *Biomacromolecules* **13**, 636-644 (2012).
4. Huang Z., *et al.* Targeted delivery of let-7b to reprogramme tumor-associated macrophages and tumor infiltrating dendritic cells for tumor rejection. *Biomaterials* **90**, 72-84 (2016).
5. Kalfeist L., *et al.* Simultaneous isolation of CD45 tumor-infiltrating lymphocytes, tumor cells, and associated fibroblasts from murine breast tumor model by MACS. *STAR Protoc* **4**, 101951 (2023).
6. Liu J., *et al.* Advanced Method for Isolation of Mouse Hepatocytes, Liver Sinusoidal Endothelial Cells, and Kupffer Cells. *Methods Mol Biol* **1540**, 249-258 (2017).
7. Schwanhäusser B., *et al.* Global quantification of mammalian gene expression control. *Nature* **473**, 337-342 (2011).
8. Geiger T., *et al.* Comparative proteomic analysis of eleven common cell lines reveals ubiquitous but varying expression of most proteins. *Mol Cell Proteomics* **11**, M111.014050 (2012).
9. Cox J. r., *et al.* Accurate proteome-wide label-free quantification by delayed normalization and maximal peptide ratio extraction, termed MaxLFQ. *Mol Cell Proteomics* **13**, 2513-2526 (2014).
10. Föcking M., *et al.* Proteomic analysis of the postsynaptic density implicates synaptic function and energy pathways in bipolar disorder. *Transl Psychiatry* **6**, e959 (2016).
11. Ménard O., *et al.* Characterization of immunostimulatory CpG-rich sequences from different Bifidobacterium species. *Appl Environ Microbiol* **76**, 2846-2855 (2010).
