# Supplementary material for: Bacterial protoplast-derived nanovesicles carrying CRISPR-Cas9 tools re-educate tumor-associated macrophages for enhanced cancer immunotherapy
Source: Nat Commun. 2024 Jan 31;15:950. doi: 10.1038/s41467-024-44941-9 (PMC10830495; doi:10.1038/s41467-024-44941-9)
Supplement: Supplementary file 3 — Description of Additional Supplementary Files [file 41467_2024_44941_MOESM3_ESM.pdf]

Description of Additional Supplementary Files

File Name: Supplementary Data 1

Description: Information of CpG-rich DNA sequences in *E. coli* genomes

File Name: Supplementary Data 2

Description: The top 200 proteins identified from *E. coli*

File Name: Supplementary Data 3

Description: The top 200 proteins identified from *E. coli* protoplast

File Name: Supplementary Data 4

Description: The top 200 proteins identified from *E. coli*-OMV

File Name: Supplementary Data 5

Description: The top 200 proteins identified from sg*Pik3cg*-DHP/DGA-NV

File Name: Supplementary Data 6

Description: The DEGs (differentially expressed genes) list in RNA-seq of 4T1 tumor tissue from sg*Pik3cg*-DHP/DGA-NVs and control mice

File Name: Supplementary Data 7

Description: Gene ontology (GO) enrichment analysis of DEGs of 4T1 tumor tissue from sg*Pik3cg*-DHP/DGA-NVs and control mice

File Name: Supplementary Data 8

Description: KEGG (Kyoto Encyclopedia of Genes and Genomes) enrichment analysis of DEGs of 4T1 tumor tissue from sg*Pik3cg*-DHP/DGA-NVs and control mice

File Name: Supplementary Data 9

Description: GSEA enrichment analysis of genes from 4T1 tumor tissue of sg*Pik3cg*-DHP/DGA-NVs treated group compared with control group

File Name: Supplementary Data 10

Description: The primer sequences used for PCR and q-PCR assay, sgRNA targeting *Pik3cg* and siRNA targeting *Mgl1/2*

File Name: Supplementary Data 11

Description: The antibodies used for flow cytometry (FC), western blotting (WB), immunofluorescence (IF) and IHC
