# Supplementary material for: Bacterial protoplast-derived nanovesicles carrying CRISPR-Cas9 tools re-educate tumor-associated macrophages for enhanced cancer immunotherapy
Source: Nat Commun. 2024 Jan 31;15:950. doi: 10.1038/s41467-024-44941-9 (PMC10830495; doi:10.1038/s41467-024-44941-9)
Supplement: Supplementary file 15 — Reporting Summary [file 41467_2024_44941_MOESM15_ESM.pdf]

nature portfolio

Corresponding author(s): Junfeng Zhang

Last updated by author(s): Dec 8, 2023

Reporting Summary

Nature Portfolio wishes to improve the reproducibility of the work that we publish. This form provides structure for consistency and transparency in reporting. For further information on Nature Portfolio policies, see our Editorial Policies and the Editorial Policy Checklist.

Statistics

For all statistical analyses, confirm that the following items are present in the figure legend, table legend, main text, or Methods section.

n/a	Confirmed	
[ ]	[x]	The exact sample size (*n*) for each experimental group/condition, given as a discrete number and unit of measurement
[ ]	[x]	A statement on whether measurements were taken from distinct samples or whether the same sample was measured repeatedly
[ ]	[x]	The statistical test(s) used AND whether they are one- or two-sided<br>*Only common tests should be described solely by name; describe more complex techniques in the Methods section.*
[x]	[ ]	A description of all covariates tested
[ ]	[x]	A description of any assumptions or corrections, such as tests of normality and adjustment for multiple comparisons
[ ]	[x]	A full description of the statistical parameters including central tendency (e.g. means) or other basic estimates (e.g. regression coefficient) AND variation (e.g. standard deviation) or associated estimates of uncertainty (e.g. confidence intervals)
[ ]	[x]	For null hypothesis testing, the test statistic (e.g. *F*, *t*, *r*) with confidence intervals, effect sizes, degrees of freedom and *P* value noted<br>*Give P values as exact values whenever suitable.*
[x]	[ ]	For Bayesian analysis, information on the choice of priors and Markov chain Monte Carlo settings
[x]	[ ]	For hierarchical and complex designs, identification of the appropriate level for tests and full reporting of outcomes
[x]	[ ]	Estimates of effect sizes (e.g. Cohen's *d*, Pearson's *r*), indicating how they were calculated

Our web collection on statistics for biologists contains articles on many of the points above.

Software and code

Policy information about availability of computer code

Data collection	1.TEM images were captured using transmission electron microscopy JEOL JEM-2100.<br>2.Flow cytometry data were acquired using Thermo Fisher Attune NxT.<br>3.Haematoxylin and eosin (H&E) staining were captured using Nikon DS-Ri2 confocal microscope.<br>4.All immunostaining pictures were captured using ZEISS LSM 980 confocal microscope and DS-Ri2 confocal microscope (Nikon).<br>5.WB images were collected by Tanon-4200SF.<br>6.Quantitative real-time PCR data were collected using qRT-PCR assay by StepOne™ Real-Time PCR System (Thermo Fisher).<br>7.Gel imaging were captured by Tanon-GIS-2500.<br>8. Particle size, zeta potential and PDI were collected using Nanosight NS300 (Malvern, United Kingdom), Nano-Z (Malvern) and NanoBrook 90Plus Zeta (Brookhaven, Holtsville, NY, USA).<br>9. In vivo imaging were captured by IVIS Spectrum, PerkinElmer Inc.<br>10.LC-MS analysis on a Shimadzu UFLC 20ADXR HPLC system in-line with an AB Sciex 5600 Triple TOF mass spectrometer (AB SCIEX, Framingham, Massachusetts State, USA).

Data analysis	1.Flow cytometry data were analyzed using FlowJo V10.<br>2.Protein subcellular localization were analyzed sing Psortdb (https://db.psort.org/). COG analysis was used to determine the function categories of protein (http://eggnog-mapper.embl.de/).<br>3.Statistical analysis was performed using Graphpad Prism 8.<br>4.The mutation frequencies, staining intensity measurement of immunostaining pictures and IHC score were analyzed using Image J.<br>5.The protein LC/MS data were processed using MaxQuant (v. 1.6.7.0).

For manuscripts utilizing custom algorithms or software that are central to the research but not yet described in published literature, software must be made available to editors and reviewers. We strongly encourage code deposition in a community repository (e.g. GitHub). See the Nature Portfolio guidelines for submitting code & software for further information.

Data

Policy information about availability of data

All manuscripts must include a data availability statement. This statement should provide the following information, where applicable:

- Accession codes, unique identifiers, or web links for publicly available datasets
- A description of any restrictions on data availability
- For clinical datasets or third party data, please ensure that the statement adheres to our policy

The next-generation sequencing data generated in this study have been deposited in the Sequence Read Archive (SRA) repository under accession code PRJNA1018426 (https://www.ncbi.nlm.nih.gov/sra/?term=PRJNA1018426). RNA sequencing data are available in the Gene Expression Omnibus (GEO)/NCBI public database under accession code GSE243428(https://www.ncbi.nlm.nih.gov/geo/query/acc.cgi?acc=GSE243428). The mass spectrometry proteomics data have been deposited to the ProteomeXchange Consortium (http://proteomecentral.proteomexchange.org) via the iProX partner repository with the dataset identifier PXD045507(https://www.iprox.cn/page/project.html?id=IPX0007165000). The obtained data of mass spectrometry proteomics was processed and then searched using the integrated Andromeda search engine against the UniProt database for E. coli BL21 (DE3)(https://www.uniprot.org/uniprotkb?facets=reviewed%3Afalse&query=BL21%28DE3%29&view=cards). The remaining data are available within the Article, Supplementary Information or Source Data file. Source data are provided in this paper.

Research involving human participants, their data, or biological material

Policy information about studies with human participants or human data. See also policy information about sex, gender (identity/presentation), and sexual orientation and race, ethnicity and racism.

Reporting on sex and gender	The study did not involve human research participants.
Reporting on race, ethnicity, or other socially relevant groupings	n/a
Population characteristics	n/a
Recruitment	n/a
Ethics oversight	n/a

Note that full information on the approval of the study protocol must also be provided in the manuscript.

Field-specific reporting

Please select the one below that is the best fit for your research. If you are not sure, read the appropriate sections before making your selection.

[x] Life sciences [ ] Behavioural & social sciences [ ] Ecological, evolutionary & environmental sciences

For a reference copy of the document with all sections, see nature.com/documents/nr-reporting-summary-flat.pdf

Life sciences study design

All studies must disclose on these points even when the disclosure is negative.

Sample size	Sample sizes were indicated in the legend of each Figure and Supplementary Figure. No statistical tests were performed to pre-determine sample size. Sample sizes of all animal and cell studies were determined based on the basis of previous experiments in the lab and previous publications using similar methodologies (Yanxian Feng et al. Nature communications, 2020, PMID: 31118418). The sample sizes were sufficient for statistical analysis.
Data exclusions	No data were excluded.
Replication	Experiments were independently repeated as indicated and experimental findings were reproducible. The number of independent experiments and biological replicates is indicated in the figure legends.
Randomization	Mice were assigned randomly into experimental groups and processed in an arbitrary order. Age were matched between experimental groups.

Blinding | We needed to investigate the difference between different groups, and such difference had not been known. Thus we did not use blinding in the study.

Reporting for specific materials, systems and methods

We require information from authors about some types of materials, experimental systems and methods used in many studies. Here, indicate whether each material, system or method listed is relevant to your study. If you are not sure if a list item applies to your research, read the appropriate section before selecting a response.

Materials & experimental systems

n/a	Involved in the study
☐	☒ Antibodies
☐	☒ Eukaryotic cell lines
☒	☐ Palaeontology and archaeology
☐	☒ Animals and other organisms
☒	☐ Clinical data
☒	☐ Dual use research of concern
☒	☐ Plants

Methods

n/a	Involved in the study
☒	☐ ChIP-seq
☐	☒ Flow cytometry
☒	☐ MRI-based neuroimaging

Antibodies

Antibodies used

1.BV421 anti-mouse F4/80, BioLegend,cat #:123137,Clone:BM8
2.APC anti-mouse CD86, BioLegend,cat #:105012,Clone:GL-1
3. PE/Cy7 anti-mouse CD45, Biolegend, cat # :103114, Clone: 30-F11
4. PE anti-mouse CD31,Biolegend, cat #:102408,Clone:390
5. AF594-Vimentin, abcam, cat #:ab154207,Clone: EPR3776
6. Goat-anti rabbit IgG (AF488), abcam, cat #: ab150077
7. Anti-albumin, abcam, cat #: ab207327, Clone: EPR20195
8.FITC anti-mouse CD45,Biolegend, cat #:103108,Clone: 30-F11
9.AF700 anti-mouse CD3,Biolegend,cat #:100216,Clone:17A2
10. BV711 anti-mouse CD4,Biolegend,cat #:100550,Clone: RM4-5
11.PE/Cy7 anti-mouse CD8a,Biolegend,cat #:100722,Clone:53-6.7
12.APC anti-mouse Ki67,Biolegend,cat #:652406,Clone:16A8
13.PE anti-mouse GranzymeB,Biolegend,cat #:372208,Clone:QA16A02
14.PE/Cy7 anti-mouse CD11b,Biolegend,cat #:101216,Clone: M1/70
15.BV711 anti-mouse F4/80,Biolegend,cat #:123147,Clone:BM8
16.BV421 anti-mouse CD86,Biolegend,cat #:105032 ,Clone:GL-1
17.PE anti-mouse CD206, BioLegend,cat #:141706,Clone:C068C2
18.BV605 anti-mouse Ly6G, BioLegend,cat #:127639,Clone:1A8
19.AF700 anti-mouse Ly6C,BioLegend,cat #:128024,Clone:HK1.4
20.BV605 anti-mouse IFN-γ,BioLegend,cat #:505840,Clone: XMG1.2
21.PE anti-mouse CD19,BioLegend,cat #:115508 ,Clone:6D5
22.PE/Cy7 anti-mouse CD49b, BioLegend,cat #:108922,Clone:DX5
23.PE/Cy7 anti-mouse MHC Ⅱ ,BioLegend,cat #:107630,Clone:M5/114.15.2
24.PE anti-mouse CD11c,BioLegend,cat #:117308,Clone: N418
25.APC anti-mouse CD80,BioLegend,cat #:104714,Clone:16-10A1
26.anti-Cas9, Abcam, cat # ab189380:,Clone: EPR18991
27.anti-RecA, MBL,cat #:MD-01-3,Clone: ARM193
28.anti-PI3Kγ,Cell Signaling Technology,cat #:5405,Clone: D55D5
29.HRP-conjugated goat anti-rabbit IgG,Jackson ImmunoResearch,cat #:111-035-003
30.anti-Histone,H3 BOSTER,cat #:A12477-2
31.HRP conjugated anti-GAPDH,Proteintech,cat #:HRP-60004,Clone: 1E6D9
32.Anti-p-Stat1,Cell Signaling Technology,cat #:9167 ,Clone: 58D6
33.Anti-Stat1,Cell Signaling Technology,cat #:9172
34.Anti-p-TAK1,Cell Signaling Technology,cat #:9339
35.Anti-TAK1,Cell Signaling Technology,cat #:4505
36.Anti- p-IRAK4,Abcam,cat #:ab216513
37.Anti-IRAK4,Cell Signaling Technology,cat #:4363
38.Anti-p-Stat3,Cell Signaling Technology,cat #:9145 Clone: D3A7
39.Anti-Stat3,Cell Signaling Technology,cat #:4904 Clone: 79D7
40.Anti-p-C/EBPβ,Cell Signaling Technology,cat #:3084
41.Anti-C/EBPβ,Cell Signaling Technology,cat #:3087
42.Anti-p-p65,Cell Signaling Technology,cat #:3033 ,Clone: 93H1
43.Anti- p65,Cell Signaling Technology,cat #:8242 ,Clone: D14E12

44.Anti-AKT,Cell Signaling Technology,cat #:4685 ,Clone: 11E7
45.Anti-p-AKT,Cell Signaling Technology,cat #:9271
46.anti-F4/80,Cell Signaling Technology,cat #:30325,Clone: D4C8V
47.anti-MGL,Biolegend,cat #:145702,Clone: LOM-14
48.Alexa Fluor 546 donkey anti-mouse IgG,Invitrogen,cat #:A10036
49.Alexa Fluor 488 donkey anti-rabbit IgG,Invitrogen,cat #:A21206
50.anti-CD86,Abcam,cat #:ab220188,Clone: C86/1146
51.anti-CD206,Abcam,cat #:ab64693
52.anti-IL-10,BOSTER,cat #:M00021
53.anti-IL-12,BOSTER,cat #:A00918
54.anti-TGF-β1,abcam,cat #:ab179695: Clone:EPR18163
55.anti-TNF-α,BOSTER,cat #:BA0131
56.anti-IFN-γ,Biolegend,cat #:505801,Clone:XMG1.2,Clone: XMG1.2
57.Anti-PD-1,BioXcell,cat #:BE0146 ,Clone: RMP1-14
58.Anti-CD8,BioXcell, cat #:BE0117 ,Clone: YTS169.4
59.Anti-TNF-α,BioXcell,cat #:BE0058 ,Clone:XT3.11
60.PE anti-mouse MGL, Biolegend, cat #:145704, Clone: LOM-14

Validation

All antibodies were verified by the manufacturers and all validation statements can be found on the respective antibody website:
1. BV421 anti-mouse F4/80 https://www.biolegend.com/en-us/products/brilliant-violet-421-anti-mouse-f4-80-antibody-7199
2. APC anti-mouse CD86 https://www.biolegend.com/en-us/products/apc-anti-mouse-cd86-antibody-2896
3. PE/Cy7 anti-mouse CD45, https://www.biolegend.com/en-us/products/pe-cyanine7-anti-mouse-cd45-antibody-1903
4. PE anti-mouse CD31, https://www.biolegend.com/en-us/products/pe-anti-mouse-cd31-antibody-122
5. AF594-Vimentin, https://www.abcam.cn/products/primary-antibodies/alexa-fluor-594-vimentin-antibody-epr3776-cytoskeleton-marker-ab154207.html
6. Goat-anti rabbit IgG (AF488): https://www.abcam.cn/products/secondary-antibodies/goat-rabbit-igg-hl-alexa-fluor-488-ab150077.html
7. Anti-albumin: https://www.abcam.cn/products/primary-antibodies/albumin-antibody-epr20195-ab207327.html
8. FITC anti-mouse CD45 https://www.biolegend.com/en-us/products/fitc-anti-mouse-cd45-antibody-99
9. AF700 anti-mouse CD3 https://www.biolegend.com/en-us/products/alexa-fluor-700-anti-mouse-cd3-antibody-3375
10. BV711 anti-mouse CD4 https://www.biolegend.com/en-us/products/brilliant-violet-711-anti-mouse-cd4-antibody-7925
11. PE/Cy7 anti-mouse CD8a https://www.biolegend.com/en-us/products/pe-cyanine7-anti-mouse-cd8a-antibody-1906
12. APC anti-mouse Ki67 https://www.biolegend.com/en-us/products/apc-anti-mouse-ki-67-antibody-8447
13. PE anti-mouse GranzymeB https://www.biolegend.com/en-us/products/pe-anti-human-mouse-granzyme-b-recombinant-antibody-14431
14. PE/Cy7 anti-mouse CD11b https://www.biolegend.com/en-us/products/pe-cyanine7-anti-mouse-human-cd11b-antibody-1921
15. BV711 anti-mouse F4/80 https://www.biolegend.com/en-us/products/brilliant-violet-711-anti-mouse-f4-80-antibody-10705
16. BV421 anti-mouse CD86 https://www.biolegend.com/en-us/products/brilliant-violet-421-anti-mouse-cd86-antibody-7282
17. PE anti-mouse CD206 https://www.biolegend.com/en-us/products/pe-anti-mouse-cd206-mmr-antibody-7424
18. BV605 anti-mouse Ly6G https://www.biolegend.com/en-us/products/brilliant-violet-605-anti-mouse-ly-6g-antibody-12244
19. AF700 anti-mouse Ly6C, https://www.biolegend.com/en-us/products/alexa-fluor-700-anti-mouse-ly-6c-antibody-6757
20. BV605 anti-mouse IFN-γ https://www.biolegend.com/en-us/products/brilliant-violet-605-anti-mouse-ifn-gamma-antibody-8114
21 PE anti-mouse CD19 https://www.biolegend.com/en-us/products/pe-anti-mouse-cd19-antibody-1530
22. PE/Cy7 anti-mouse CD49b https://www.biolegend.com/en-us/products/pe-cyanine7-anti-mouse-cd49b-pan-nk-cells-antibody-8058
23. PE/Cy7 anti-mouse MHC Ⅱ https://www.biolegend.com/en-us/products/pe-cyanine7-anti-mouse-i-a-i-e-antibody-6136
24. PE anti-mouse CD11c https://www.biolegend.com/en-us/products/pe-anti-mouse-cd11c-antibody-1816
25. APC anti-mouse CD80 https://www.biolegend.com/en-us/products/apc-anti-mouse-cd80-antibody-2340
26. anti-Cas9 https://www.abcam.cn/products/primary-antibodies/crispr-cas9-antibody-epr18991-ab189380.html
27.anti-RecA https://www.mblintl.com/products/md-01-3/
28.anti-PI3Kγ https://www.cellsignal.cn/products/primary-antibodies/pi3-kinase-p110g-d55d5-rabbit-mab/5405
29.HRP-conjugated goat anti-rabbit IgG https://www.jacksonimmuno.com/catalog/products/111-035-003
30.anti-Histone, H3 https://www.boster.com.cn/index/products/productsDetail?goods_sn=A12477-2
31.HRP conjugated anti-GAPDH https://www.ptgcn.com/products/GAPDH-Antibody-HRP-60004.htm
32.Anti-p-Stat1 https://www.cellsignal.cn/products/primary-antibodies/phospho-stat1-tyr701-58d6-rabbit-mab/9167
33.Anti-Stat1 https://www.cellsignal.cn/products/primary-antibodies/stat1-antibody/9172
34.Anti-p-TAK1 https://www.cellsignal.cn/products/primary-antibodies/phospho-tak1-ser412-antibody/9339
35.Anti-TAK1 https://www.cellsignal.cn/products/primary-antibodies/tak1-antibody/4505
36.Anti- p-IRAK4 https://www.abcam.cn/products/primary-antibodies/irak4-phospho-t345-antibody-ab216513.html
37.Anti-IRAK4 https://www.cellsignal.cn/products/primary-antibodies/irak4-antibody/4363
38.Anti-p-Stat3 https://www.cellsignal.cn/products/primary-antibodies/phospho-stat3-tyr705-d3a7-xp-rabbit-mab/9145
39.Anti-Stat3 https://www.cellsignal.cn/products/primary-antibodies/stat3-79d7-rabbit-mab/4904
40.Anti-p-C/EBPβ https://www.cellsignal.cn/products/primary-antibodies/phospho-c-ebpb-thr235-antibody/3084
41.Anti-C/EBPβ https://www.cellsignal.cn/products/primary-antibodies/c-ebpb-lap-antibody/3087
42.Anti-p-p65 https://www.cellsignal.cn/products/primary-antibodies/phospho-nf-kb-p65-ser536-93h1-rabbit-mab/3033
43.Anti- p65 https://www.cellsignal.cn/products/primary-antibodies/nf-kb-p65-d14e12-xp-rabbit-mab/8242
44.Anti-AKT https://www.cellsignal.cn/products/primary-antibodies/akt-pan-11e7-rabbit-mab/4685
45.Anti-p-AKT https://www.cellsignal.cn/products/primary-antibodies/phospho-akt-ser473-antibody/9271

46.anti-F4/80 https://www.cellsignal.cn/products/primary-antibodies/f4-80-d4c8v-xp-rabbit-mab/30325
47.anti-MGL https://www.biolegend.com/en-us/products/purified-anti-mouse-cd301-mgl1-mgl2-antibody-8460
48.Alexa Fluor 546 donkey anti-mouse IgG https://www.thermofisher.cn/cn/zh/antibody/product/Donkey-anti-Mouse-IgG-H-L-Highly-Cross-Adsorbed-Secondary-Antibody-Polyclonal/A10036
49.Alexa Fluor 488 donkey anti-rabbit IgG https://www.thermofisher.cn/cn/zh/antibody/product/Goat-anti-Rabbit-IgG-H-L-Cross-Adsorbed-Secondary-Antibody-Polyclonal/A-11008
50.anti-CD86 https://www.abcam.cn/products/primary-antibodies/cd86-antibody-c861146-ab220188.html
51.anti-CD206 https://www.abcam.cn/products/primary-antibodies/mannose-receptor-antibody-ab64693.html
52.anti-IL-10 https://www.boster.com.cn/index/products/productsDetail?goods_sn=M00021-1
53.anti-IL-12 https://www.boster.com.cn/index/products/productsDetail?goods_sn=A00918-1
54.anti-TGF-β1 https://www.abcam.cn/products/primary-antibodies/tgf-beta-1-antibody-epr18163-ab179695.html
55.anti-TNF-α https://www.boster.com.cn/index/products/productsDetail?goods_sn=BA0131
56.anti-IFN-γ https://www.biolegend.com/en-us/products/purified-anti-mouse-ifn-gamma-antibody-998
57.Anti-PD-1 https://www.bioxcell.com.cn/in-vivo-antibodies/invivomab-anti-mouse-pd-1-cd279-be0146.html
58.Anti-CD8 https://www.bioxcell.com.cn/product/catalogsearch.html?q=BE0117%20
59.Anti-TNF-α https://www.bioxcell.com.cn/in-vivo-antibodies/invivomab-anti-mouse-tnf%CE%B1-be0058.html
60. PE anti-mouse MGL https://www.biolegend.com/en-us/products/pe-anti-mouse-cd301-mgl1-mgl2-antibody-8461

Eukaryotic cell lines

Policy information about cell lines and Sex and Gender in Research

Cell line source(s)	4T1 cells (catalog number (cat. no.): SCSP-5056), Raw 264.7 cells (cat. no. SCSP-5036), 293T cells (cat. no. SCSP-502) were obtained by Cell Bank, Chinese Academy of Sciences (Shanghai, China). MC38 cells (cat. no.: 1101MOU-PUMC000523) was obtained from the Cell Resource Center, Peking Union Medical College (Beijing, China).
Authentication	These cell lines were authenticated by the supplier using STR analysis
Mycoplasma contamination	All cell lines are negative for mycoplasma contamination.
Commonly misidentified lines (See ICLAC register)	No commonly misidentified cell lines were used.

Animals and other research organisms

Policy information about studies involving animals; ARRIVE guidelines recommended for reporting animal research, and Sex and Gender in Research

Laboratory animals	Female BALB/c mice and C57BL/6J mice (6 weeks old) were procured from Beijing Vital River Laboratory Animal Technology Co. Ltd (Beijing, China). C57BL/6Smoc-Tlr9em1Smoc (TLR9 KO) mice were obtained from Shanghai Model Organisms Center Inc. (Shanghai, China) and 6-week-old female TLR9 KO mice were used in this study. All animals were housed in a specific pathogen-free (SPF) environment with 21±2 °C and a relative humidity of 55±10%, with a 12 h light/12 h dark cycle and free access to standard food and water.
Wild animals	No wild animals were used in the study.
Reporting on sex	All animal data was collected from female mice. The experiment was designed without considering the sex of the mice, and female mice were selected to ensure gender uniformity.
Field-collected samples	No field collected samples were used in the study.
Ethics oversight	Animal protocols were reviewed and approved by the Animal Care and Use Committee of Nanjing University, and conformed to the Guidelines for the Care and Use of Laboratory Animals published by the National Institutes of Health.

Note that full information on the approval of the study protocol must also be provided in the manuscript.

Flow Cytometry

Plots

Confirm that:

- [x] The axis labels state the marker and fluorochrome used (e.g. CD4-FITC).
- [x] The axis scales are clearly visible. Include numbers along axes only for bottom left plot of group (a 'group' is an analysis of identical markers).
- [x] All plots are contour plots with outliers or pseudocolor plots.
- [x] A numerical value for number of cells or percentage (with statistics) is provided.

Methodology

Sample preparation	Cell suspensions from the blood, spleen, liver, tumor tissues or cell lines were filtered through Nylon cell strainers (70 μM, Falcon, USA), and red blood cells were lysed for primary cells suspensions. The cells were rinsed with PBS and further blocked with Fc-antibody diluting in PBS containing 1% BSA on ice for 10 min. For cell surface molecules staining, 1×10^6 cells were incubated with corresponding fluorescence-labeled antibodies for 30 min on ice and then washed with 1% BSA. 7-AAD staining was used for distinguishing live and dead cells. For intracellular staining, tumor leukocytes were first blocked with Fc-antibody, stained with antibodies against with cell surface molecules, fixated and permeabilized with BD Cytofix/Cytoperm solution (BD Bioscience, San Jose, NJ, USA), and then stained with antibodies against IFN-γ, ki67 and Granzyme B. For IFN-γ staining, tumor leukocytes were firstly treated with Cell Activation Cocktail (with Brefeldin A) (Biolegend, San Diego, CA, USA) for 4 h before surface molecules staining. The Zombie Violet™ Fixable Viability Kit (Biolegend) was applied to distinguish live cells from dead cells. Flow cytometry was performed on an Attune NxT device (Thermo Fisher), and the data were analyzed with FlowJo v10.0 software (BD Biosciences, Ashland, OR, USA).
Instrument	Thermo Fisher Attune NxT
Software	FlowJo V10
Cell population abundance	Data on the abundance of relevant cell populations are provided in the paper.
Gating strategy	Representative gating strategies are shown in supplementary Fig.36-41.

☒ Tick this box to confirm that a figure exemplifying the gating strategy is provided in the Supplementary Information.
